# Integrative taxonomy resolves the cryptic and pseudo-cryptic *Radula buccinifera* complex (Porellales, Jungermanniopsida), including two reinstated and five new species

**DOI:** 10.3897/phytokeys.27.5523

**Published:** 2013-10-30

**Authors:** Matt A.M. Renner, Nicolas Devos, Jairo Patiño, Elizabeth A. Brown, Andrew Orme, Michael Elgey, Trevor C. Wilson, Lindsey J. Gray, Matt J. von Konrat

**Affiliations:** 1 Royal Botanic Gardens and Domain Trust, Mrs Macquaries Road, Sydney, NSW 2000, Australia; 2 Department of Biology, Duke University, Box 90388, Durham NC 27708, U.S.A.; 3 Institute of Botany, University of Liège, Liège, Belgium; 4 School of Biological Sciences, The University of Sydney, NSW 2006, Australia; 5 The Field Museum of Natural History, 1400 South Lake Shore Drive, Chicago, Illinois, USA

**Keywords:** Radulaceae, *Radula* subg. *Metaradula*, *Radula anisotoma*, *Radula australiana*, *Radula buccinifera*, *Radula demissa*, *Radula imposita*, *Radula mittenii*, *Radula notabilis*, *Radula pugioniformis*, *Radula strangulata*, sp. nov., morphology, DNA sequence data, Australia, New Zealand, Flora, liverwort, dispersal, biogeography, cryptic species

## Abstract

Molecular data from three chloroplast markers resolve individuals attributable to *Radula buccinifera* in six lineages belonging to two subgenera, indicating the species is polyphyletic as currently circumscribed. All lineages are morphologically diagnosable, but one pair exhibits such morphological overlap that they can be considered cryptic. Molecular and morphological data justify the re-instatement of a broadly circumscribed ecologically variable *R. strangulata*, of *R. mittenii*, and the description of five new species. Two species *Radula mittenii* Steph. and *R. notabilis* **sp. nov.** are endemic to the Wet Tropics Bioregion of north-east Queensland, suggesting high diversity and high endemism might characterise the bryoflora of this relatively isolated wet-tropical region. *Radula demissa* **sp. nov.** is endemic to southern temperate Australasia, and like *R. strangulata* occurs on both sides of the Tasman Sea. *Radula imposita* **sp. nov.** is a twig and leaf epiphyte found in association with waterways in New South Wales and Queensland. Another species, *R. pugioniformis* **sp. nov.**, has been confused with *Radula buccinifera* but was not included in the molecular phylogeny. Morphological data suggest it may belong to subg. *Odontoradula*. *Radula buccinifera* is endemic to Australia including Western Australia and Tasmania, and to date is known from south of the Clarence River on the north coast of New South Wales. Nested within *R. buccinifera* is a morphologically distinct plant from Norfolk Island described as *R. anisotoma* **sp. nov.** *Radula australiana* is resolved as monophyletic, sister to a species occurring in east coast Australian rainforests, and nesting among the *R. buccinifera* lineages with strong support. The molecular phylogeny suggests several long-distance dispersal events may have occurred. These include two east-west dispersal events from New Zealand to Tasmania and south-east Australia in *R. strangulata*, one east-west dispersal event from Tasmania to Western Australia in *R. buccinifera*, and at least one west-east dispersal from Australia to New Zealand in *R. australiana*. Another west-east dispersal event from Australia to Norfolk Island may have led to the budding speciation of *R. anisotoma*. In contrast, *Radula demissa* is phylogeographically subdivided into strongly supported clades either side of the Tasman Sea, suggesting long distance dispersal is infrequent in this species.

## Introduction

Crypsis is thought to be a widespread phenomenon in bryophytes (Bischler and Boisselier-DuBayle 1997; Shaw 2001). Molecular phylogenetic investigations of bryophytes often resolve populations of morphological species in widely separated clades suggesting some taxa are comprised of morphologically convergent yet relatively unrelated populations (Shaw and Allen 2000; Stech and Wagner 2005; Fernandez et al. 2006; Karlin et al. 2008; Hutsemékers et al. 2012; Carter 2012). Morphologically cryptic and semi-cryptic lineages have been demonstrated in more than 200 studies of bryophyte species (Heinrichs et al. 2006), and nearly all major liverwort lineages including Porellales (Ramaiya et al. 2010; Heinrichs et al. 2009a, 2010; Hentschel et al. 2007; Renner et al. 2011); Jungermanniales (Feldberg et al. 2007); Metzgeriales (Wachowiak et al. 2007; Preussing et al. 2010; Fiedorow et al. 2001; Fuselier et al. 2009); and the complex thalloid genera *Conocephalum* (Odrzykoski and Szweykowski 1991; Szweykowski et al. 2005), *Reboulia* (Boisselier-Dubayle et al. 1998) and *Dumortiera* (Forrest et al. 2011). Cryptic species represent more than a trivial debate over the application of species concepts and the implications of cryptic diversity for studies of biodiversity, biogeography, and evolutionary ecology are widely recognised. Cryptic lineages may conceal the malleability of taxonomically important character systems, and so bias our reconstruction of the history of character evolution (Vanderpoorten et al. 2001, 2002; Ho et al. 2012; Yu et al. 2010; Heinrichs et al. 2011; Pons et al. 2011).

**Figure 1.**
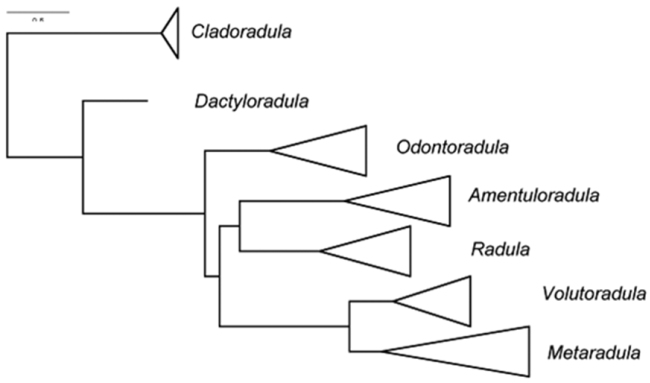
Relationships between seven subgenera identified within *Radula* by Devos et al. (2011a, b).

The genus *Radula* is named for a morphologically distinctive lineage belonging to the Porellales (Davis 2004; Forrest et al. 2006) which comprises the groups Porellinae and Radulinae *sensu* Schuster (1980). Morphological features characterising Porellinae include incubous leaf insertion, conduplicately-bilobed leaves, lack of ventral branching, and rhizoids in fascicles. The Porellales has a global distribution with peak diversity in tropical regions where high rates of endemism are reached (Schuster 1980; Vanderpoorten et al. 2010). Morphologically, *Radula* is highly divergent within Porellales (see Schuster 1980) and is characterised by several potentially synapomorphic characters including loss of the ventral merophyte row and production of rhizoids from the dorsal surface of the leaf at the apex of the lobule carinal region. So unusual are these features that Schuster (1958) attributed *Radula* to its own suborder. *Radula* has been resolved as a monophyletic genus and there is currently little doubt regarding its integrity (Devos et al. 2011a, b). This contrasts with the infrageneric classification, where all four subgenera proved poly- or paraphyletic (Devos et al. 2011b), and species were resolved on seven fully supported main lineages (Fig. 1). Identification of the genus in the field is straightforward, the incubously inserted, conduplicately-bilobed leaves, the lack of underleaves, the production of rhizoids in clusters from the surface of the ventral leaf lobe, and the dorso-ventrally compressed perianths (from which the generic name is derived), combined with the above mentioned synapomorphies make the genus distinctive. However, due to morphological plasticity and variation in character states (So 2005), the circumscription of many species is debated (e.g. So 2005, 2006; Renner 2005, 2006), and estimates of global diversity range from around 200 (Gradstein and Costa 2003) to 350 (Yamada 1979). Reported variation in species estimates for both geographical regions and lineages reflects a contemporary debate about the utility of typological species concepts within bryophytes (Heinrichs et al. 2009a) that has significant consequences for estimates of global species diversity (von Konrat et al. 2010a).

**Figure 2.**
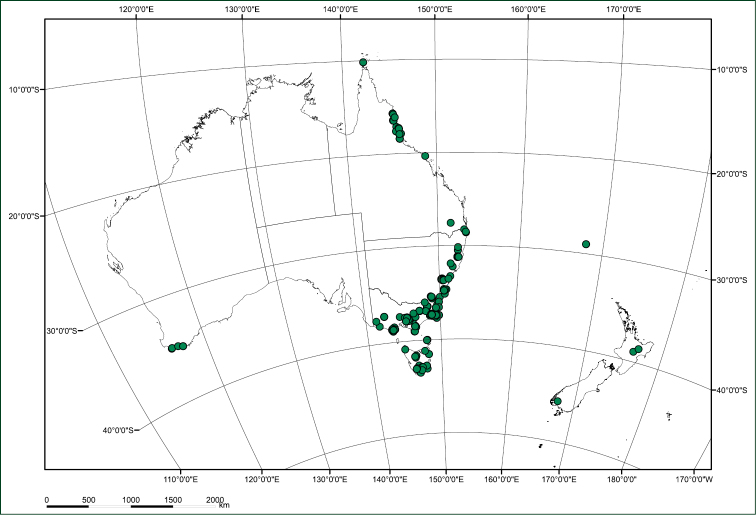
Distribution of *Radula buccinifera* according to data presented online by Australia’ s Virtual Herbarium. Data courtesy of The Council of Heads of Australasian Herbaria 2013, Australia’s Virtual Herbarium. http://avh.chah.org.au [Accessed 25 April 2013].

**Figure 3.**
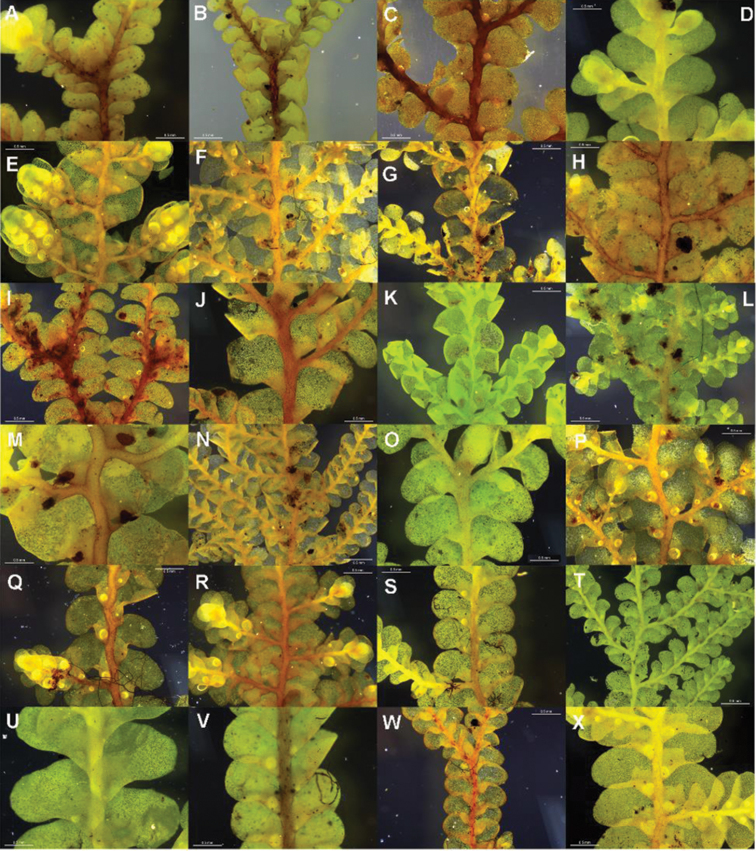
Morphological variation expressed by individuals included in this study, showing variation in shoot size, branching pattern, and lobule shape. All species represented by the individuals shown belong to the *Radula buccinifera* species complex **A** *Radula strangulata* de Lange 10167 **B** *Radula demissa* NSW895246 **C** *Radula strangulata* NSW875811 **D** *Radula mittenii* NSW875805 **E** *Radula australiana* NSW909252 **F** *Radula demissa* NSW895267 **G** *Radula demissa* NSW970835 **H** *Radula strangulata* NSW970841 **I** *Radula anisotoma* CANB650458 **J** *Radula buccinifera* NSW895271 **K** *Radula demissa* NSW895272 **L** *Radula demissa* NSW909292 **M** *Radula strangulata* NSW909416 **N** *Radula demissa* NSW909424 **O** *Radula buccinifera* NSW909436 **P** *Radula demissa* NSW895397 **Q** *Radula demissa* NSW895439 **R** *Radula demissa* NSW896177 **S** *Radula notabilis* NSW896419 **T** *Radula imposita* NSW896812 **U** *Radula mittenii* NSW897206 **V** *Radula notabilis* NSW909500 **W** *Radula demissa* NSW909482 **X** *Radula mittenii* NSW896672. Scale bars 0.5 mm in all images.

Thirty one and seventeen species of *Radula* are currently accepted in Australia and New Zealand respectively following studies on Queensland, Tasmanian and New Zealand species (Yamada 1984; So 2005; Renner 2006; Renner et al. 2010a). The most common and widespread Australasian species is *Radula buccinifera* (Hook.f. & Taylor) Taylor ex Gottsche, Lindenb. & Nees, which is distributed from south-west Western Australia through Victoria, Tasmania, eastern New South Wales, and across the Tasman to New Zealand and the Chatham Islands 900 km east of Christchurch (Fig. 2). *Radula buccinifera* has a latitudinal range from the subantarctic Campbell and Auckland islands in the south (52°30'S), to Moa Island in Torres Strait at the tip of Cape York Peninsula in the tropics (10°10'S). Across this geographic range, *Radula buccinifera* can be found in a wide variety of habitat types, including tropical and subtropical lowland forest, tropical montane rainforest, warm and cool temperate rainforest including those dominated by *Nothofagus*, wet sclerophyll forest, sub-alpine shrubland, and even alpine grassland and herbfield. Within this remarkable diversity of habitats *Radula buccinifera* can be found on an equally remarkable variety of substrates, including on leaves, twigs, branches, naked bark and bryophyte mats on tree trunks, rotting logs, clay soil banks and on rocks including those almost permanently submerged beneath running water. Coupled with the exceptional geographical and ecological variation exhibited by *Radula buccinifera* is considerable morphological diversity (Fig. 3). *Radula buccinifera* was last revised in 2005, in a study that expanded the number of synonyms from four to six, and circuitously concluded that ‘*Radula buccinifera* has been described under several different names, indicating the variability exhibited by the various forms’ (So 2005). Within Australasia *Radula buccinifera* is therefore a typical example of a widespread variable liverwort species.

Morphologically circumscribed bryophyte species tend to have broader geographical distributions than angiosperm species (Shaw 2001; Vanderpoorten et al. 2010), and within an Australasian context *Radula buccinifera* does not appear to be an exception. This may be a real pattern attributable to spore-based reproduction and the relative ease with which spores are transported and new populations established (Muñoz et al. 2004; Pohjamo et al. 2006, Cassie et al. 2008; Hutsemékers et al. 2008), or an artefact of failing to detect cryptic or semi-cryptic species (Heinrichs et al. 2009b; Medina et al. 2012). In the Australasian region 290 species and 200 genera of seed plants (c. 9% of New Zealand’s flora) are indigenous to both Australia and New Zealand (Wilton and Breitwieser 2000; McGlone et al. 2001; de Lange et al. 2007), 90 of New Zealand’s c. 200 fern species are shared with Australia (Brownsey 2001), and 125 of 500 moss and 284 of New Zealand’s 595 liverwort species also occur in Australia (Engel and Glenny 2008a; Perrie et al. 2010) suggesting widespread exchange of all floristic components via long-distance dispersal (LDD). Indeed, LDD in bryophytes has frequently been identified by molecular phylogenetic studies (e.g. McDaniel and Shaw 2003; Shaw 2001; Rycroft et al. 2002; Stech and Dohrmann 2004; Heinrichs et al. 2006, 2009a; Feldberg et al. 2007; Shaw et al. 2008; Hentschel et al. 2009; Dong et al. 2012; Patiño et al. 2013a), and trans-Tasman (McDaniel and Shaw 2003; Perrie et al. 2010) and trans-Antarctic (Pokorny et al. 2011) dispersal events have been inferred.

Although cryptic haplotype diversity was detected within *Radula lindenbergiana* (Laenen et al. 2011), no cryptic species complexes have yet been identified within *Radula*. The only molecular phylogenetic study at species level, also conducted on the Macaronesian flora, found agreement between morphological and molecular species circumscription (Stech et al. 2010). However, a recent global phylogeny for the genus included three representatives of *Radula buccinifera* that formed a clade within which *Radula australiana*, a morphologically distinctive species, was nested (Devos et al. 2011a, b). In this study we assess evidence for or against a broad circumscription (i.e. a widespread, ecotypically and morphologically variable species) of *Radula buccinifera* and whether *Radula australiana* should be subsumed within *Radula buccinifera*. We sequence multiple accessions of *Radula australiana* and *Radula buccinifera* encompassing the geographical, ecological and morphological variation exhibited by the species as currently circumscribed. Our results provide the first demonstration of cryptic species in *Radula*, and one of the most extreme cases yet documented in liverworts in terms of neglected (i.e. not previously recognised at any level) diversity uncovered by phylogenetic data. Though subtle morphological differences between most entities mean the complex is dominated by semi-cryptic species, one pair of species can, by virtue of overlapping morphological variation, be considered genuinely cryptic. This study demonstrates the generality but also the potential severity of the phenomenon of cryptic and semi-cryptic species in liverworts.

## Materials and methods

### Taxon sampling and molecular protocols.

Sampling for DNA was based on material collected haphazardly throughout the geographical range reported for *Radula australiana* and *Radula buccinifera* and including New Zealand, Norfolk Island, Tasmania, Victoria, New South Wales, Queensland and Western Australia. At each site one to ten collections representing the morphological and ecological diversity exhibited by *Radula australiana* and *Radula buccinifera* were made. The objective of sampling was to include multiple individuals of each morphological type from many sites across both species distribution. Unpublished sequences from individuals attributable to *Radula australiana* and *Radula buccinifera* were included in the dataset (N.Devos unpublished data). Individuals of species belonging to subg. *Metaradula* were included to increase the severity of our test of the relationship between individuals of *Radula buccinifera*, particularly the test of monophyly. The 93 accessions included in the global phylogeny published by Devos et al. (2011a, b) were included in this analysis, to establish the degree to which *Radula buccinifera* is polyphyletic, if at all. Table 1 provides all information on the studied plant material including collector, locality, voucher number and GenBank accession numbers for the sequenced markers.

Clean shoot tips comprising the meristem, immature leaves, and one or two nearly mature leaves were excised from each specimen until approximately 25–50 mm^2^ of cleaned material was obtained depending on plant size. Study specimens were either stored on silica gel or rapidly air dried from wild collected material to ensure plant material remained green and free of fungus.

**Table 1.** Voucher information and GenBank accession numbers for specimens included in the molecular dataset. All ‘MR’ isolates were newly generated for this study.

**Species**	**Voucher information**		***atp*B-*rbc*L**	***trn*G**	***trn*L-F**
*Radula acuminata*	T. Pocs 02102/AA EGR	ND_227	-	HM992384	HM992463
*Radula anisotoma*	Australia, Norfolk Island H Streimann 32084A CANB650459	MR_75	-	-	KF440465
*Radula ankefinensis*	S. & T. Pocs 04011/G EGR	ND_222	-	HM992382	HM992461
*Radula antilleana*	S.R. Gradstein 9448 GOET	ND_088	-	HM992343	HM992429
*Radula apiculata*	T. Yamaguchi 1731 BR	ND_339	-	-	HM992478
*Radula appressa*	T. Pocs 90113/AH EGR	ND_229	-	HM992386	HM992465
*Radula aquilegia*	A. chafer-Verwimp & Verwimp 26039 Herb. Schafer-verwimp	ND_078	-	HM992341	HM992427
*Radula australiana*	Australia, Victoria M.A.M. Renner 5114 NSW893115	MR_1	KF432229	KF432315	KF440396
*Radula australiana*	Australia, Victoria M.A.M. Renner 5138 NSW875865	MR_10	KF432237	KF432323	KF440405
*Radula australiana*	Australia, Victoria M.A.M. Renner 5139 NSW875866	MR_11	KF432238	KF432324	KF440406
*Radula australiana*	Australia, Victoria M.A.M. Renner 5142 NSW875928	MR_12	KF432239	KF432325	-
*Radula australiana*	Australia, Victoria M.A.M. Renner 5143 NSW875940	MR_13	KF432240	KF432326	KF440407
*Radula australiana*	Australia, Victoria M.A.M. Renner 5144 NSW875941	MR_14	KF432241	KF432327	KF440408
*Radula australiana*	Australia, Victoria M.A.M. Renner 5145 NSW875942	MR_15	KF432242	KF432328	KF440409
*Radula australiana*	Australia, Victoria M.A.M. Renner 5150 NSW875947	MR_16	KF432243	KF432329	KF440410
*Radula australiana*	Australia, Victoria M.A.M. Renner 5162 NSW875951	MR_17	KF432244	-	KF440411
*Radula australiana*	Australia, Victoria M.A.M. Renner 5164 NSW875953	MR_18	KF432245	KF432330	KF440412
*Radula australiana*	Australia, Victoria M.A.M. Renner 5115 NSW893116	MR_2	KF432230	KF432316	KF440397
*Radula australiana*	Australia, Victoria M.A.M. Renner 5205 NSW893128	MR_22	KF432249	KF432334	KF440416
*Radula australiana*	Australia, Victoria M.A.M. Renner 5118 NSW893119	MR_3	KF432231	KF432317	KF440398
*Radula australiana*	Australia, Victoria M.A.M. Renner 5127 NSW909241	MR_4	KF432232	KF432318	KF440399
*Radula australiana*	Australia, Victoria M.A.M. Renner 5129 NSW909251	MR_5	KF432233	KF432319	KF440400
*Radula australiana*	New Zealand, South Island M.A.M. Renner 6142 NSW895444	MR_50	KF432276	KF432362	KF440441
*Radula australiana*	New Zealand, South Island M.A.M. Renner 6148 NSW895456	MR_51	KF432277	KF432363	KF440442
*Radula australiana*	New Zealand, South Island M.A.M. Renner 6168 NSW895494	MR_52	KF432278	KF432364	KF440443
*Radula australiana*	New Zealand, South Island M.A.M. Renner 6230 NSW895690	MR_57	KF432283	KF432369	KF440447
*Radula australiana*	New Zealand, South Island M.A.M. Renner 6239 NSW896176	MR_58	KF432284	KF432370	KF440448
*Radula australiana*	New Zealand, South Island M.A.M. Renner 6241 NSW896177	MR_59	KF432285	KF432371	KF440449
*Radula australiana*	Australia, Victoria M.A.M. Renner 5130 NSW909252	MR_6	KF432234	KF432320	KF440401
*Radula australiana*	Australia, Victoria M.A.M. Renner 5131 NSW909254	MR_7	KF432235	KF432321	KF440402
*Radula australiana*	Australia, Victoria M.A.M. Renner 5133 NSW875860	MR_8	KF432236	KF432322	KF440403
*Radula australiana*	Australia, Victoria M.A.M. Renner 5135 NSW875862	MR_9	-	-	KF440404
*Radula australiana*	AK280485 AK	ND_096	-	KF186997	-
*Radula australiana*	J.A. Curnow 5635 CBG	ND_119	-	HM992356	HM992442
*Radula australiana*	H. Streimann 53505 CBG	ND_121	-	KF187009	KF187195
*Radula australiana*	J.A. Curnow 5638 CBG	ND_124	-	KF187012	KF187196
*Radula australiana*	D. Glenny CHR559976 CHR	ND_210	-	HM992377	HM992456
*Radula australis*	B. Shaw 6089 DUKE	ND_299	-	HM992399	HM992477
*Radula bipinata*	T. Pocs NY8016 NY	ND_161	-	HM992372	-
*Radula boryana*	T. Pocs 88110/AR E	ND_178	-	HM992375	-
*Radula brunnea*	N. Ohnishi H3196644 H	ND_001	-	HM992315	HM992403
*Radula buccinifera*	Australia, Victoria M.A.M. Renner 5176 NSW875959	MR_19	KF432246	KF432331	KF440413
*Radula buccinifera*	Australia, Victoria M.A.M. Renner 5177 NSW875960	MR_20	KF432247	KF432332	KF440414
*Radula buccinifera*	Australia, Victoria M.A.M. Renner 5204 NSW893126	MR_21	KF432248	KF432333	KF440415
*Radula buccinifera*	Australia, New South Wales M.A.M. Renner 5246 NSW875783	MR_23	KF432250	KF432335	KF440417
*Radula buccinifera*	Australia, New South Wales M.A.M. Renner 5257 NSW875805	MR_24	KF432251	KF432336	KF440418
*Radula buccinifera*	Australia, New South Wales M.A.M. Renner 5288 NSW875835	MR_27	KF432253	KF432339	KF440420
*Radula buccinifera*	Australia, New South Wales M.A.M. Renner 5303 NSW877190	MR_28	KF432254	KF432340	KF440421
*Radula buccinifera*	Australia, New South Wales M.A.M. Renner 5868 NSW898654	MR_29	KF432255	KF432341	KF440422
*Radula buccinifera*	Australia, Tasmania M.A.M. Renner 5939 NSW895271	MR_33	KF432259	KF432345	KF440425
*Radula buccinifera*	Australia, Tasmania M.A.M. Renner 6016 NSW909416	MR_37	KF432263	KF432349	KF440429
*Radula buccinifera*	Australia, Tasmania M.A.M. Renner 6025 NSW909425	MR_40	KF432266	KF432352	KF440432
*Radula buccinifera*	Australia, Tasmania M.A.M. Renner 6027 NSW909430	MR_41	KF432267	KF432353	KF440433
*Radula buccinifera*	Australia, Tasmania M.A.M. Renner 6032 NSW909436	MR_42	KF432268	KF432354	KF440434
*Radula buccinifera*	Australia, Western Australia E.D. Cooper 09/067 NSW970847	MR_72	KF432297	KF432382	KF440462
*Radula buccinifera*	Australia, Western Australia E.D. Cooper 09/068 NSW970854	MR_73	-	KF432383	KF440463
*Radula buccinifera*	Australia, Western Australia E.D. Cooper 09/142 NSW970856	MR_74	-	-	KF440464
*Radula buccinifera*	Australia, New South Wales EA Brown 89/35 NSW436068	MR_76	KF432298	KF432384	KF440466
*Radula buccinifera*	A. Schafer-Verwimp & Verwimp 14336 Herb. Schafer-verwimp	ND_053	-	HM992332	HM992417
*Radula buccinifera*	H. Streimann 54341 CBG	ND_127	-	HM992359	HM992444
*Radula buccinifera*	B. Shaw 6511 DUKE	ND_293	-	KF187111	KF187266
*Radula buccinifera*	B. Shaw DUKE	ND_294	-	KF187112	KF187267
*Radula buccinifera*	B. Shaw 6619 DUKE	ND_295	-	KF187113	KF187268
*Radula buccinifera*	B. Shaw DUKE	ND_297	-	KF187115	KF187270
*Radula buccinifera*	B. Shaw 6209 DUKE	ND_298	-	KF187116	KF187271
*Radula buccinifera*	B. Shaw DUKE	ND_300	-	KF187117	KF187272
*Radula buccinifera*	B. Shaw DUKE	ND_301	-	KF187118	KF187273
*Radula campanigera*	N. Ohnishi HIRO225 GOET	ND_042	-	HM992330	-
*Radula carringtonii*	A. Schafer-Verwimp & Verwimp 25734 Herb. Schafer-verwimp	ND_018	-	HM992323	HM992409
*Radula comorensis*	A. Schafer-Verwimp & Verwimp 23835 Herb. Schafer-verwimp	ND_045	-	HM992331	HM992416
*Radula compacta*	J.A. Curnow 4525 CBG	ND_126	-	HM992358	-
*Radula complanata*	B. Shaw F915 DUKE	ND_311	-	HM992393	-
*Radula constricta*	T. Koponen H3187494 H	ND_004	-	HM992317	-
*Radula cubensis*	A. Schafer-Verwimp & M. Preussing 23532 Herb. Schafer-verwimp	ND_068	-	HM992337	HM992422
*Radula decora*	I. Holz & Franzaring CH0060 GOET	ND_026	-	HM992327	HM992413
*Radula demissa*	Australia, Tasmania M.A.M. Renner 5916 NSW909267_1	MR_30	KF432256	KF432342	-
*Radula demissa*	Australia, Tasmania M.A.M. Renner 5916 NSW909267_2	MR_31	KF432257	KF432343	KF440423
*Radula demissa*	Australia, Tasmania M.A.M. Renner 5923 NSW895246	MR_32	KF432258	KF432344	KF440424
*Radula demissa*	Australia, Tasmania M.A.M. Renner 5940 NSW895272	MR_34	KF432260	KF432346	KF440426
*Radula demissa*	Australia, Tasmania M.A.M. Renner 5989 NSW909286	MR_35	KF432261	KF432347	KF440427
*Radula demissa*	Australia, Tasmania M.A.M. Renner 5998 NSW909293	MR_36	KF432262	KF432348	KF440428
*Radula demissa*	Australia, Tasmania M.A.M. Renner 6023 NSW909423	MR_38	KF432264	KF432350	KF440430
*Radula demissa*	Australia, Tasmania M.A.M. Renner 6024 NSW909424	MR_39	KF432265	KF432351	KF440431
*Radula demissa*	Australia, Tasmania M.A.M. Renner 6036 NSW909452	MR_43	KF432269	KF432355	
*Radula demissa*	Australia, Tasmania M.A.M. Renner 6048 NSW909482	MR_44	KF432270	KF432356	KF440435
*Radula demissa*	New Zealand, South Island M.A.M. Renner 6076 NSW895351	MR_45	KF432271	KF432357	KF440436
*Radula demissa*	New Zealand, South Island M.A.M. Renner 6127 NSW895397	MR_48	KF432274	KF432360	KF440439
*Radula demissa*	New Zealand, South Island M.A.M. Renner 6137 NSW895439	MR_49	KF432275	KF432361	KF440440
*Radula demissa*	New Zealand, South Island M.A.M. Renner 6180 NSW895508	MR_53	KF432279	KF432365	KF440444
*Radula demissa*	New Zealand, South Island M.A.M. Renner 6183 NSW895511	MR_54	KF432280	KF432366	KF440445
*Radula demissa*	New Zealand, South Island M.A.M. Renner 6227 NSW895686	MR_56	KF432282	KF432368	KF440446
*Radula demissa*	New Zealand, South Island M.A.M. Renner 6244 NSW896179	MR_60	KF432286	KF432372	KF440450
*Radula demissa*	New Zealand, North Island P.J. de Lange NC16 NSW970835	MR_79	KF432301	KF432387	KF440469
*Radula demissa*	Australia, Tasmania M.A.M. Renner 5936 NSW895267	MR_90	KF432312	KF432398	KF440480
*Radula demissa*	AK254565 AK	ND_107	-	KF187003	-
*Radula demissa*	AK280339 AK	ND_110	-	KF187005	KF187191
*Radula dentifolia*	M.A.M. Renner AK280588 AK	ND_111	-	HM992353	HM992439
*Radula eggersii*	A. Schafer-Verwimp & M. Preussing 23330/A Herb. Schafer-verwimp	ND_058	-	HM992334	HM992420
*Radula episcia*	S. Churchill, M. Serrano et al. MO23708 MO	ND_148	-	HM992366	HM992449
*Radula evelynae*	T. Pocs, R.E. Magill & A. Rupf 9288/R EGR	ND_234	-	HM992389	HM992468
*Radula fendleri*	A. Schafer-Verwimp & M. Preussing 23250/A Herb. Schafer-verwimp	ND_074	-	HM992339	HM992424
*Radula flaccida*	A. Schafer-Verwimp, J. Heinrichs, R.A. Wilson & S.O. Yandun 24422 GOET	ND_072	-	HM992338	HM992423
*Radula floridana*	B. Shaw 6209 DUKE	ND_323	-	HM992396	HM992474
*Radula formosa*	T. Pocs s.n. EGR	ND_240	-	HM992392	HM992471
*Radula frondescens*	I. Holz CR000493 GOET	ND_091	-	HM992345	HM992431
*Radula fruticosa*	U. Drehwald NY970175 NY	ND_154	-	HM992368	HM992451
*Radula fulvifolia*	T. Pocs s.n. EGR	ND_215	-	HM992379	HM992458
*Radula gottscheana*	S. Ingram & K. Ferrell-Ingram Ingram1765	ND_060	-	HM992335	-
*Radula grandis*	D. Glenny CHR571846 CHR	ND_212	-	-	HM992457
*Radula hastata*	S.R. Gradstein 9443 GOET	ND_090	-	HM992344	HM992430
*Radula helix*	M.A.M. Renner AK282969 AK	ND_098	-	HM992347	HM992433
*Radula hicksiae*	J.A. Curnow & H. Streimann 3689 CBG	ND_120	-	HM992357	HM992443
*Radula holstiana*	Hodgetts M2668a E	ND_185	-	HM992376	HM992455
*Radula holtii*	N. Devos & A. Vanderpoorten DV003 DUKE	ND_281	-	HM992398	HM992476
*Radula husnoti*	M.J. Lyon DB12895 MO	ND_015	-	HM992322	HM992408
*Radula imposita*	Australia, New South Wales M.A.M. Renner 5275 NSW875821_1	MR_26	KF432252	KF432338	KF440419
*Radula imposita*	Australia, New South Wales M.A.M. Renner 5275 NSW875821_2	MR_85	KF432307	KF432393	KF440475
*Radula imposita*	Australia, Queensland M.A.M. Renner 6356 NSW896812	MR_89	KF432311	KF432397	KF440479
*Radula iwatsukii*	A. Schafer-Verwimp & Verwimp 18757/A Herb. Schafer-verwimp	ND_076	-	-	HM992426
*Radula japonica*	M. Higuchi 1198 BR	ND_353	-	HM992402	HM992481
*Radula javanica*	S. Churchill, M. Decker & F. Morgo MO22187 MO	ND_142	-	HM992365	HM992448
*Radula jonesii*	N. Devos s.n. DUKE	ND_267	-	HM992397	HM992475
*Radula kegelii*	N. Salazar DB3609 GOET	ND_012	-	HM992320	HM992406
*Kojana*	M. Mizutani 14255 DUKE	ND_137	-	HM992364	HM992447
*Radula lindenbergiana*	A. Schafer-Verwimp & Verwimp 25732/A Herb. Schafer-verwimp	ND_063	-	HM992336	HM992421
*Radula macroloba*	T. Pocs s.n. EGR	ND_238	-	HM992391	HM992470
*Radula macrostachya*	S.R Gradstein & G. Dauphin DB12894 GOET	ND_007	-	HM992318	HM992404
*Radula madagascariensis*	A. Szabo 9614/DV EGR	ND_232	-	HM992387	HM992466
*Radula majorezica*	T. Pocs 90103/AE EGR	ND_233	-	HM992388	HM992467
*Radula mazarunensis*	A. Schafer-Verwimp & Verwimp 17767 Herb. Schafer-verwimp	ND_081	-	HM992342	HM992428
*Radula mexicana*	A. Schafer-Verwimp & M. Preussing 23204 Herb. Schafer-verwimp	ND_036	-	HM992329	HM992415
*Radula mittenii*	Australia, Queensland M.A.M. Renner 6489 NSW897206	MR_80	KF432302	KF432388	KF440470
*Radula mittenii*	Australia, Queensland M.A.M. Renner 6288 NSW896672	MR_81	KF432303	KF432389	KF440471
*Radula mittenii*	Australia, Queensland M.A.M. Renner 6296 NSW896685	MR_82	KF432304	KF432390	KF440472
*Radula mittenii*	Australia, Queensland M.A.M. Renner 6486 NSW897201	MR_83	KF432305	KF432391	KF440473
*Radula mittenii*	Australia, Queensland M.A.M. Renner 6497 NSW909664	MR_84	KF432306	KF432392	KF440474
*Radula mittenii*	Australia, Queensland M.A.M. Renner 6282 NSW896665	MR_86	KF432308	KF432394	KF440476
*Radula multiamentula*	M.A.M. Renner AK280299 AK	ND_108	-	HM992352	HM992438
*Radula multiflora*	K.R. Wood NY9604 NY	ND_166	-	HM992373	HM992453
*Radula neotropica*	B. Allen NY11935 NY	ND_160	-	HM992371	HM992452
*Radula notabilis*	Australia, Queensland M.A.M. Renner 6275 NSW896419	MR_64	KF432289	KF432375	KF440454
*Radula notabilis*	Australia, Queensland M.A.M. Renner 6276 NSW896657	MR_65	KF432290	-	KF440455
*Radula notabilis*	Australia, Queensland M.A.M. Renner 6487 NSW897204	MR_66	KF432291	KF432376	KF440456
*Radula notabilis*	Australia, Queensland M.A.M. Renner 6504 NSW909497	MR_67	KF432292	KF432377	KF440457
*Radula notabilis*	Australia, Queensland M.A.M. Renner 6505 NSW909500	MR_68	KF432293	KF432378	KF440458
*Radula notabilis*	Australia, Queensland M.A.M. Renner 6506 NSW909501	MR_69	KF432294	KF432379	KF440459
*Radula notabilis*	Australia, Queensland M.A.M. Renner 6507 NSW909502	MR_70	KF432295	KF432380	KF440460
*Radula nudicaulis*	A. Schafer-Verwimp & M. Preussing 23447 Herb. Schafer-verwimp	ND_020	-	HM992325	HM992411
*Radula nymanii*	Australia, Queensland M.A.M. Renner 2277 NSW909661	MR_87	KF432309	KF432395	KF440477
*Radula nymanii*	Australia, Queensland M.A.M. Renner 6510 NSW898712	MR_88	KF432310	KF432396	KF440478
*Radula obconica*	B. Shaw 4874 DUKE	ND_135	-	HM992363	HM992446
*Radula obtusiloba*	W.B. Schofield 115550 DUKE	ND_133	-	HM992362	-
*Radula ocellata*	J.A. Curnow 3664 CBG	ND_116	-	HM992354	HM992440
*Radula perrottetii*	M. Mizutani NY15272 NY	ND_158	-	HM992369	-
*Radula physoloba*	M.A.M. Renner CHR555962 CHR	ND_211	-	HM992378	-
*Radula plicata*	M.A.M. Renner AK280391 AK	ND_103	-	HM992351	HM992437
*Radula plumosa*	J. Hyvonen DB3600 GOET	ND_011	-	HM992319	HM992405
*Radula pocsii*	S. Churchill, M. Serrano et al. MO23444 MO	ND_150	-	HM992367	HM992450
*Radula polyclada*	B. Shaw F956 DUKE	ND_315	-	HM992394	HM992472
*Radula prolifera*	W.B. Schofield 115792 DUKE	ND_131	-	HM992361	HM992445
*Radula pulchella*	H. Streimann 63817 EGR	ND_219	-	HM992380	HM992459
*Radula quadrata*	T. Pocs, E.M. Kungu & A. Szabo 9230/S EGR	ND_225	-	HM992383	HM992462
*Radula queenslandica*	J.A. Curnow 3846 CBG	ND_118	-	HM992355	HM992441
*Radula ratkowskiana*	Australia, Tasmania M.A.M. Renner 5933 NSW895261	MR_77	KF432299	KF432385	KF440467
*Radula ratkowskiana*	M.A.M. Renner AK280205 AK	ND_102	-	HM992350	HM992436
*Radula recubans*	M. Burghardt DB21422 GOET	ND_092	-	HM992346	HM992432
*Radula reflexa*	T. Pocs s.n. EGR	ND_220	-	HM992381	HM992460
*Radula retroflexa*	S. & T. Pocs 03281/C EGR	ND_228	-	HM992385	HM992464
*Radula robinsonii*	Australia, Queensland M.A.M. Renner 2271 NSW885024	MR_91	KF432313	KF432399	KF440481
*Radula saccatiloba*	A. Schafer-Verwimp & Verwimp 18053 Herb. Schafer-verwimp	ND_075	-	HM992340	HM992425
*Radula schaefer-verwimpii*	A. Schafer-Verwimp & M. Preussing 23443/A Herb. Schafer-verwimp	ND_019	-	HM992324	HM992410
*Radula stenocalyx*	T. Pocs s.n. EGR	ND_235	-	HM992390	HM992469
*Radula stipatiflora*	T. Arts R…U52/24 BR	ND_346	-	HM992400	HM992479
*Radula strangulata*	Australia, New South Wales M.A.M. Renner 5265 NSW875811	MR_25	-	KF432337	-
*Radula strangulata*	New Zealand, South Island M.A.M. Renner 6082 NSW895357	MR_46	KF432272	KF432358	KF440437
*Radula strangulata*	New Zealand, South Island M.A.M. Renner 6092 NSW895367	MR_47	KF432273	KF432359	KF440438
*Radula strangulata*	New Zealand, South Island M.A.M. Renner 6222 NSW895673	MR_55	KF432281	KF432367	-
*Radula strangulata*	New Zealand, South Island M.A.M. Renner 6259 NSW896393	MR_61	KF432287	KF432373	KF440451
*Radula strangulata*	New Zealand, North Island M.A.M. Renner 6265 NSW896405	MR_62	KF432288	KF432374	KF440452
*Radula strangulata*	New Zealand, North Island M.A.M. Renner 6266 NSW896409	MR_63	-	-	KF440453
*Radula strangulata*	New Zealand, North Island P.J. de Lange 10167 AK327986	MR_71	KF432296	KF432381	KF440461
*Radula strangulata*	New Zealand, North Island P.J. de Lange NC14 NSW970841	MR_78	KF432300	KF432386	KF440468
*Radula strangulata*	M.A.M. Renner AK280392 AK	ND_099	-	HM992348	HM992434
*Radula strangulata*	AK286375 AK	ND_100	-	KF186999	KF187188
*Radula strangulata*	CHR525056 CHR	ND_204	-	KF187062	KF187218
*Radula striata*	U. Drehwald 970175 BR	ND_352	-	HM992401	HM992480
*Radula subinflata*	I. Holz & Schafer-Verwimp DB13093 GOET	ND_030	-	HM992328	HM992414
*Radula sulivantii*	B. Shaw 6189 DUKE	ND_321	-	HM992395	HM992473
*Radula tasmanica*	M.A.M. Renner AK280184 AK	ND_101	-	HM992349	HM992435
*Radula tenax*	P.G. Davison & M.L. Hicks 2946 DUKE	ND_129	-	HM992360	-
*Radula tenera*	A. Schafer-Verwimp, J. Heinrichs, R.A. Wilson & S.O. Yandun 24230 Herb. Schafer-verwimp	ND_022	-	HM992326	HM992412
*Radula tjibodensis*	Vanuatu, E.A. Brown s.n. NSW971057	MR_92	KF432314	KF432400	KF440482
*Radula tjibodensis*	A.L. Ilkiu-Borges, S.R. Gradstein, K.T. Yong & M. Ponniah DB16663 GOET	ND_055	-	-	HM992418
*Radula tokiensis*	T. Koponen H3187760 H	ND_003	-	HM992316	-
*Radula varilobula*	S.R. Hill NY21274 NY	ND_167	-	HM992374	HM992454
*Radula voluta*	A. Vanderpoorten AVW857 LG	ND_014	-	HM992321	HM992407
*Radula wichurae*	A. Schafer-Verwimp & Verwimp 26018 Herb. Schafer-verwimp	ND_057	-	HM992333	HM992419

Total genomic DNA was extracted using the DNeasy Plant Minikit (QIAGEN Pty Ltd, Sydney Australia). Three chloroplast markers were sequenced, (1) the *atp*B-*rbc*L spacer, (2) the plastid *trn*L-F region including the *trn*LUAA group1 intron and the *trn*L-F intergenic spacer, hereafter *trn*L-F, and (3) the *trn*G G2 intron. Primer details are presented in Table 2. Polymerase chain reaction (PCR) was carried out using the following protocols. For *trn*L-F each 15 µl reaction contained 1.5 µl 10× PCR Buffer, 1.5 µl 20 mM MgCl_2_, 0.9 µl of each primer at 10 µM concentration, 0.12 µl of 1% BSA, and 0.12 µl of Immolase Taq. For the *atp*B-*rbc*L and *trn*G each 15 µl reaction contained 1.5 µl 10× PCR Buffer, 0.75 µl 20 mM MgCl_2_, 0.9 µl of each primer at 10 µM concentration, 0.12 µl of 1% BSA, and 0.08 µl of Immolase Taq. Temperature profile used for sequencing was 95 °C for 10 minutes, then 35 cycles of 95 °C for 1 min, 1 min at annealing temperature of 53 °C for *trn*L-F and *trn*G, and 50 °C for *atp*B-*rbc*L, then 72 °C for 1 min, followed by a final extension step of 72 ° C for 10 min. The same primers were used for sequencing of cleaned PCR products by Macrogen Inc., South Korea (www.macrogen.com).

**Table 2.** Primers used in this study for amplification and sequencing of three chloroplast DNA regions.

**Region**	**Primer**	**Sequence 5’-3’**	**Direction**	**References**
*atp*B-*rbc*L	*atp*B-1	ACATCKARTACKGGACCAATAA	Forward	Chiang et al. (1998)
	*rbc*L-1	AACACCAGCTTTRAATCCAA	Reverse	Chiang et al. (1998)
*trn*L-*trn*F	A50272	ATTTGAACTGGTGACACGAG	Forward	Taberlet et al. (1991)
	B49317	CGAAATCGGTAGACGCTACG	Reverse	Taberlet et al. (1991)
*trn*G	*trn*GF	ACCCGCATCGTTAGCTTG	Forward	Pacak and Szweykowska-Kulinska (2000)
	*trn*GR	GCGGGTATAGTTTAGTGG	Reverse	Pacak and Szweykowska-Kulinska (2000)

### DNA sequence alignmnt and phylogenetic analyses

For each DNA region, forward (5’–3’) and reverse (3’–5’) sequences were assembled and checked for inaccurate base calling using Geneious (Drummond et al. 2012). Consensus sequences were aligned by MUSCLE (Edgar 2004) on the CIPRES portal (Miller et al. 2010) and manually edited in BioEdit 5.0.9 (Hall 1999) following alignment rules and principals of homology outlined in Kelchner (2000) and Morrison (2006).

Maximum parsimony (MP) analyses were performed on individual markers, which revealed no significant (≥ 70% bootstrap support) incongruence among markers, so markers were concatenated for subsequent analyses. MP analyses were run using the parsimony ratchet (Nixon 1999) performed by PAUP* v4.0 (Swofford 2002) on the CIPRES portal. Branch swapping was performed using TBR and 500 random addition sequence replicates, and the strict consensus of most parsimonious trees computed. Autapomorphic and constant characters were excluded prior analysis. Data consistency was assessed by conducting 1000 bootstrap replicates with 10 random addition sequence replicates per bootstrap replicate, in PAUP* v4.0. Clades were considered supported if present in ≥ 70% and strongly supported if present in ≥ 90 % of trees from each replicate.

Maximum likelihood (ML) analyses were implemented in GARLI (Zwickl 2006). A single GTR (six state model) was applied to the whole dataset, with parameters estimated from the data. Rough initial optimization was performed from a random seed, and the analysis run until 15,000 generations had passed without improvement in tree topology. Ten independent runs having different random seeds yielded the same tree topology. Clade support under likelihood criterion was estimated using ML bootstrap in RAxML v7.2.6 (Stamatakis 2006) provided on the CIPRES portal (Miller et al. 2010). Analyses used the GTRGAMMA model and replication was terminated at the discretion of the program.

Bayesian analysis was performed with a hybrid version of MrBayes (Huelsenbeck and Ronquist 2001; Pratas et al. 2009) on the CIPRES portal. For each partition the best-fit substitution model was selected according to the Akaike Information Criterion (AIC) calculated by jModelTest 0.1.1 (Posada 2008). Substitution models for each partition were unlinked. Default program settings specified a priori probabilites for other parameters. Phylogenies were estimated using two independent Metropolis coupled MCMC runs comprising one cold and three heated chains run simultaneously for 7.9 million generations (the number completed in a 24 hour period) and sampled every 1000^th^ generation. Convergence between the four runs was assessed by comparing the trace files for each using Tracer 1.5 (Rambaut and Drummond 2009) and after plotting log likelihood values the first 500 samples were discarded as burnin.

### Morphology

Specimens of *Radula buccinifera* from AK, BM, BRI, CANB, CHR, FH, G, MEL, MPN, NSW, NY, PERTH, S, and WELT were examined. Morphology was assessed with the aid of dissecting and compound microscopes. In the descriptions, measurements are given for the lowest and highest observed values for a given structure, so are indicative of range only. Leaf lobe length is perpendicular to the stem axis, width is parallel to the axis, and does not include the lobule. Lobule length and width are measured parallel and perpendicular respectively to the line between the apex and base of the keel (the lobe-lobule junction and the postical extremity of the stem insertion line). Lobule size and shape were assessed by rehydrating and mounting material on a slide for investigation with a compound microscope. This is essential for any meaningful comparison, as deformation associated with dehydration obscures subtle shape differences. For identification, lobule shape should always be assessed on the basis of hydrated material, and is best assessed from slide-mounted shoots with a compound microscope, however differences can, with practice, be observed in the field with a 20× handlens in good light.

Stem transverse sections were prepared by hand from primary shoots, with sections taken from three different shoots for each individual, and slide mounted in water for observation. Dissections of female bracts, gynoecia, and archegonia were by hand with the aid of a pair of Inox #5 ‘Biologie’ tweezers and slide mounted in water. Perianth longitudinal sections were also prepared by hand, with two or three perianths from a selection of individuals examined for each species depending on availability, and slide mounted in water for examination.

Observations of species ecology were made during fieldwork for various purposes in New Zealand and Australia from 2000 to 2013. Geographic data was drawn from digitised collections, in particular AVH, and from geo-referenced specimens.

Capsule and perianth lengths for three specimens of *Radula strangulata* were measured with an eyepiece micrometer on a compound microscope from perianths with dehisced sporophytes in situ. Capsule length is really capsule valve length. Correlation statistics were calculated using the stats package in R 2.12.0 (The R Foundation for Statistical Computing 2010, http://www.R-project.org)

### Species concept

Species described here are formal placeholders for hypotheses explaining the distribution of character data from multiple sources, including morphology, ecology, geography, and molecular sequence data among individuals (see Fitzhugh 2005). These hypotheses are derived from an explanatory model wherein restricted gene flow between groups facilitates acquisition and maintenance of character state differences that in turn precipitate partial or complete reproductive isolation, and in turn ecological, and phylogenetic divergence alone or in combination. Non-compliance with any of these criteria, where exceptions can be reconciled against likely real processes past or present, is always permitted.

## Results

### Sampling

We sampled 62 and 25 individuals of *Radula buccinifera* and *Radula australiana* respectively. An additional five individuals belonging to other species of subg. *Metaradula* were included to increase the stringency of our test of *Radula buccinifera*’s monophyly. We obtained *atp*B-*rbc*L spacer sequences for 85 accessions, *trn*G for 86 accessions and *trn*L-F for 88 accessions, thereby 259 new sequences were generated for this study (Table 1). A further 101 *trn*G and 89 *trn*L-F sequences were included for unpublished *Radula buccinifera* sequences and the 93 species in the global phylogeny (Devos et al. 2011a, b). Alignments resulted in datasets of 745 putatively homologous sites for *atp*B-*rbc*L spacer, 777 for *trn*G, and 671 for *trn*L-F, the concatenated dataset had 2193 alignment positions of which 1391 were constant, 216 autapomorphic, and 586 were parsimony informative. The combined dataset included 449 of the total 591 sequences, a missing rate of 24%, mostly in the *atp*B-*rbc*L spacer which was not sequenced by Devos et al. (2011a, b). The aligned, concatenated dataset is available at Dryad (http://datadryad.org/) doi: 10.5061/dryad.h43q8

### Phylogenetic analyses

**Figure 4.**
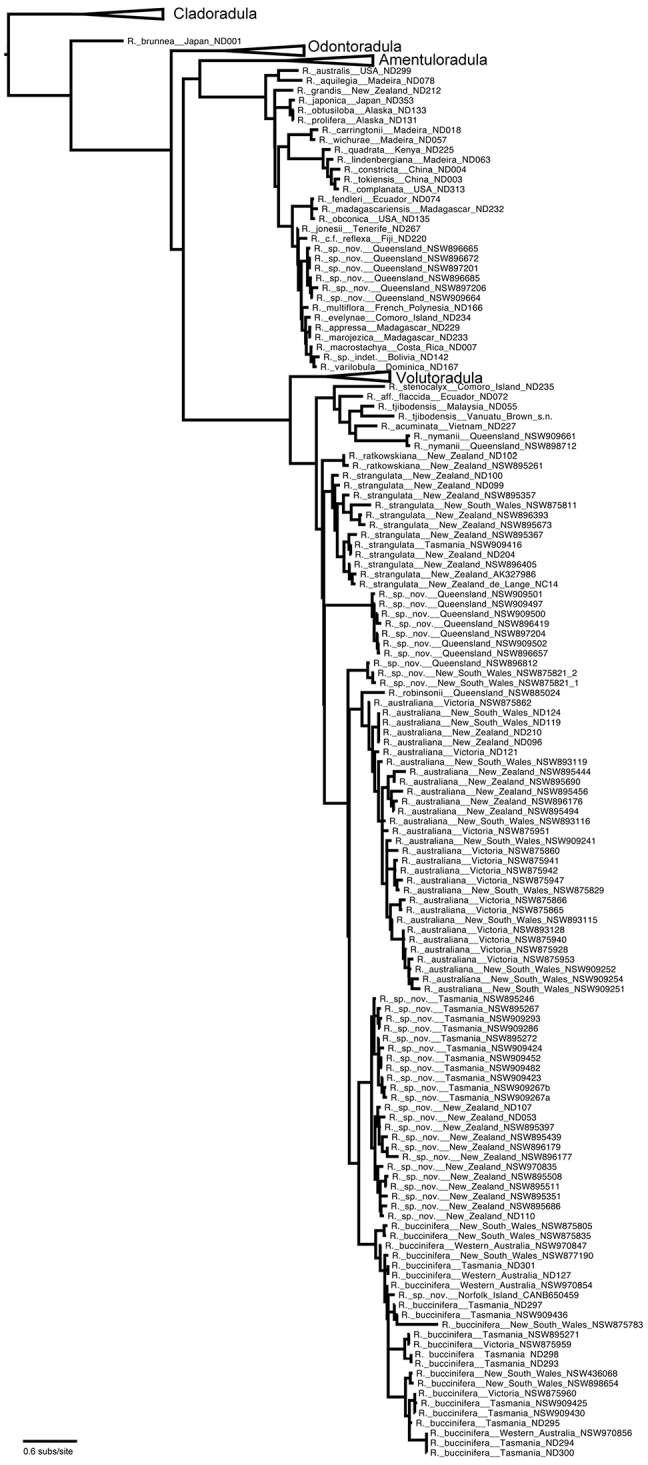
Majority rule phylogram from posterior probability distribution sampled by MrBayes showing the phylogeny of *Radula* with species named but without morphological groups identified. Branch lengths are proportional to substitution rate.

**Figure 5.**
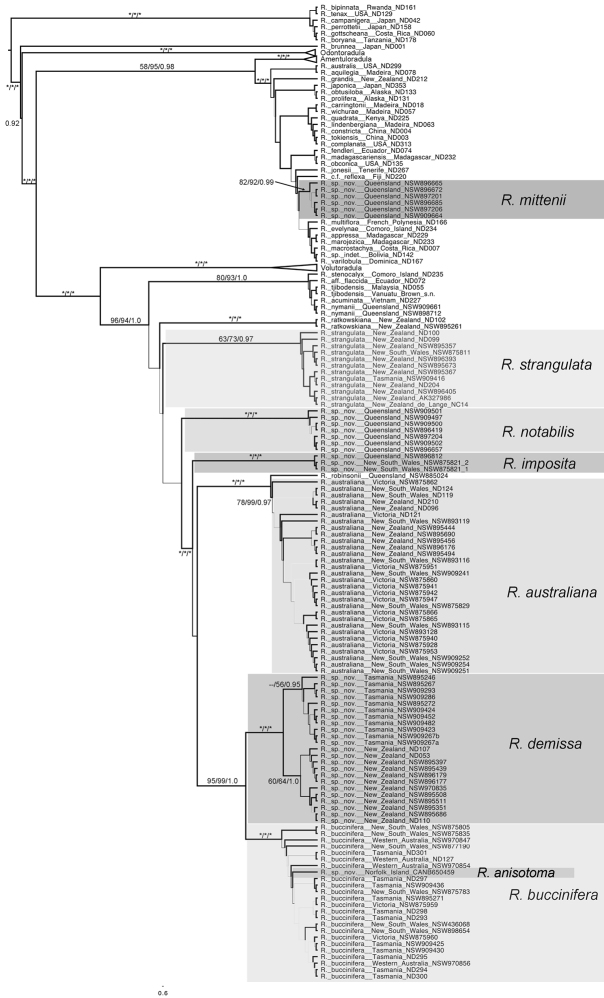
Majority Rule tree from posterior probability distribution sample taken by MrBayes, shown as a modified proportional tree, with some branch lengths shortened, for presentation purposes, with morphological groups identified. Tree topology, rather than branch length is emphasized in this tree, the branches are not to any scale. For branch lengths proportional to substitution rate refer to Figure 4 which is a phylogram with identical topology. Support values associated with each branch are parsimony bootstrap / likelihood bootstrap / posterior probability. Asterisks indicate full support. Only values for supported branches associated with the *Radula buccinifera* species complex are shown.

All data partitions converged on nearly identical topologies for supported clades, with no significant disagreement. All three partitions recover the subgeneric framework resolved in Devos et al. (2011b). In all analyses of concatenated data the tree topology recovered contained the same seven major lineages as Devos et al. (2011a, b) all of which were again fully supported, with the same relationships between subgenera (Figs 4, 5). Individuals of *Radula buccinifera* were resolved in two fully supported subgenera, six in subg. *Radula* and the remainder in subg. *Metaradula*. Within subg. *Metaradula*, *Radula buccinifera* individuals are resolved in six strongly or fully supported clades with long stems relative to tips (Fig. 4). These clades subdivide the geographical range, microhabitat diversity, and morphological variation exhibited by *Radula buccinifera*. For clarity and consistency these clades are referred to by name throughout the results.

The six individuals of *Radula mittenii* are resolved in a fully supported clade nested within subg. *Radula* (Figs 4, 5). *Radula mittenii* exhibits a number of distinctive morphological features that separates it from other members of the species complex. In life, the plants have a distinctive milky-yellow appearance that is preserved to a greater or lesser degree in herbarium material. This milky lustre may be due to the finely verrucose ornamentation on the surfaces of leaf cells. While finely verrucose leaf surface ornamentation is found in some other species of subg. *Radula*, including *Radula madagascariensis* and *Radula reflexa*, it does not occur in any other member of the *Radula buccinifera* complex. *Radula mittenii* individuals are relatively large, and regularly pinnately branched compared to other individuals, the lobules have a large ampliate interior free margin that, on primary shoots, covers and obscures the stem in ventral view. The stem section of this entity has nodular to confluent trigones throughout the stem section. Perianths have a low basal stem perigynium and a calyptra perigynium has a multistratose base. Individuals of this lineage occur on tree trunks or the sides of granite boulders in tropical rainforest habitats from sea level to 1600 m in the Wet Tropics Bioregion of north-east Queensland.

Relationships at the base of subg. *Metaradula* are not well resolved (Figs 4, 5). One clade containing a number of epiphyllous taxa from the paleotropics is resolved sister to the remainder of the subgenus without support. Relationships between *Radula ratkowskiana*, a morphologically distinctive species (Renner and Braggins 2004, 2005), and other species within subg. *Metaradula* are also unsupported.

*Radula strangulata* was represented by 11 individuals from New Zealand, one from Tasmania and one from New South Wales, and was resolved with high posterior probability. While this species is phenetically heterogeneous, individuals share several morphological features including rotund leaf-lobes spreading and usually held more or less appressed to the substrate so that the dorsal stem surface is visible between the leaves and tending remote in plants from hyper-humid and wet sites. The lobules are typically triangular when small, longitudinally oblong with increasing size, and have an acuminate apex with small but abruptly ampliate free interior margin when maximally developed. A dorsal leaf-free strip two or three cell rows wide is present. The perianth has a plane mouth and well-developed stem perigynium. Ecologically, plants occur in a variety of microsites close to ground level within forest interiors, including tree-trunk bases, rotting logs, soil banks, rocks along and within streams, sometimes under flowing water. The morphology of these plants corresponds to a number of types, including those for *Radula levieri* Steph., *Radula silvosa* E.A.Hodgs. et Allison, and *Radula strangulata* Hook.f. & Taylor, with the last name having priority.

The seven individuals of *Radula notabilis* are resolved in a clade with full support, although relationships between this species and others are again unsupported. All individuals of *Radula notabilis* were collected as trunk, branch or twig epiphytes in tropical lowland forests, growing on naked bark. They all have a distinct brown green hue and hold their leaves closely appressed to the substrate so that the dorsal stem surface is visible between the leaves. In addition the plants are sparingly branched, with shoots tending to run parallel along the substrate. The lobule is longitudinally rectangular, the stem in transverse section has heavily brown-pigmented cortical cell walls, and bulging trigones at the angles of the medulla cells. The undulate and repand perianth mouth is distinctive.

Other species of the *Radula buccinifera* complex, including *Radula australiana*, are resolved in a fully supported clade.

*Radula imposita* is represented in the phylogeny by two individuals (one extracted and sequenced twice), one a twig epiphyte on *Sloanea australis* overhanging a stream in the North Coast of New South Wales, the other an epiphyll on *Normandia* leaves, also overhanging a stream in the Wet Tropics Bioregion of north-east Queensland. Both plants are small, irregularly pinnately branched, the leaves are obliquely patent, and have lobules with an attenuate apex and pyriform carinal region. The leaf cells are bulging and occasionally weakly papillose.

*Radula buccinifera* and *Radula demissa* are resolved as sister taxa with strong support.

*Radula buccinifera* is resolved with full support. Individuals of *Radula buccinifera* are relatively large, with weakly obliquely spreading leaf lobes that do not cover the entire dorsal stem surface, leaving some of the stem visible in dorsal view. The leaf insertion does not obtain the dorsal stem mid-line, leaving one to three cortical cell rows leaf-free; leaf-lobes are rotund, not falcate, the lobules, when fully developed, have an ampliate free interior margin, the apex is obtuse to acute, and the perianths have a plane mouth. These plants occur in a range of microsites in forest interiors close to the forest floor, including tree-trunk bases, rotting logs, soil banks, rocks along and within streams, sometimes under flowing water. Microhabitat diversity decreases northward, with plants from central and northern districts of New South Wales primarily lithophytic on vertical rock surfaces.

*Radula demissa* is resolved with full support. Individuals of *Radula demissa* have obliquely patent leaf-lobes that completely obscure the dorsal stem surface, the leaf-lobes are falcate. The leaf insertion attains the dorsal stem mid-line, leaving no dorsal leaf-free strip. The lobules are rhombic to widely rhombic. The perianth mouth is often weakly inrolled in immature perianths whose apex is bicornute as a result, however the mouth is usually plane in mature perianths. These plants are epiphytes on tree trunks, branches, twigs, and occasionally epiphylls in humid, well lit sites.

*Radula australiana* is resolved in a fully supported sister relationship with an unidentified accession collected as an epiphyll in the wet tropics of north-east Queensland. Phenetically this individual is similar in some respects to *Radula gedena* Gottsche ex Steph. but lacks gemmae, and has leaf-lobe cells that are uniform in size. Furthermore the leaf-lobules have a curved keel and lack a protruding mamilliform pocket in the carinal region. In the relative size of its lobules, its leaf-lobe shape and orientation, this plant does resemble *Radula australiana*. Although similar to at least one species assigned to sect. *Epiphyllae* by Castle (1939) and Yamada (1979), broader consideration of Australian species and types reveals that in details of its lobule shape, leaf-lobe cell wall architecture and stem section the plant is a good match with the type of *Radula robinsonii* Steph. (G00042708!). *Radula robinsonii* and the *Radula parvitexta* complex will be the subject of a subsequent treatment.

*Radula australiana* itself is resolved monophyletic with strong support. Despite the sister relationship with tropical species, *Radula australiana* inhabits alpine areas in New South Wales, Victoria and New Zealand. Individuals are characterised by large leaf-lobules up to one quarter the area of the leaf lobes, with acute to acuminate apex, and broadly ampliate free-interior margin, typically brown-green plants with obliquely-patent leaves that obscure the stem in dorsal view. With one exception (NSW875829) these plants were all collected from rock outcrops and rocky areas in alpine areas, often in association with watercourses, and with the same exception all plants are a good match with the isotype of *Radula australiana*.

*Radula anisotoma*, represented by a single accession from Norfolk Island nested within *Radula buccinifera*,is morphologically divergent in having small, triangular lobules, caducous leaf lobes, and elliptic-oblong female bract lobes. These features have not been observed, alone or in combination, in *Radula buccinifera* from mainland Australia and Tasmania.

## Discussion

### Taxonomic implications

The name *Radula buccinifera* is currently applied to a phylogenetically heterogeneous assemblage of six lineages belonging to two recognised subgenera. The reason is simple: characters separating morphologically distinct entities within the *Radula buccinifera* complex have been repeatedly overlooked or misinterpreted. Demolition of substandard taxonomic output by molecular phylogenetic investigations is trivial. However, molecular investigations at species level often find conflict between Linnean classifications and phylogenetic relationships even when species taxonomy based on morphology is well resolved (e.g. Heinrichs et al. 2009a, 2012). Molecular data almost always prompts an increase in recognised diversity (e.g. Ramaiya et al. 2010; Pons et al. 2011; Heinrichs et al. 2009b, 2010) by identifying cryptic and semi-cryptic species that represent knowledge gaps (Johnson and Cairns-Heath 2010; Medina et al. 2012). The degree to which our previous understanding was deficient is reflected in the severity to which extant diversity was under or over-represented by hypotheses of relationship. By this metric, the current circumscription of *Radula buccinifera* ranks among the most ineffective yet identified among bryophytes, rivalled by *Conocephalum conicum* (Odrzykoski and Szweykowski 1991; Kim et al. 1996, 2001) and *Frullania tamarisci* (Heinrichs et al. 2010). However, both *Conocephalum conicum* and *Frullania tamarisci* have near-global distributions, whereas *Radula buccinifera* is confined to Australasia. While cryptic species within both *Conocephalum conicum* and *Frullania tamarisci* are mostly allopatric, in *Radula buccinifera* up to three entities may be found growing sympatrically. Furthermore, pairs of entities often grow syntopically, which in part explains the admixtures found in voucher specimens and, more problematically, type material.

Most published examples of cryptic diversity within bryophytes come from the northern hemisphere in particular Europe and North America (e.g. Baczkiewicz et al. 2008; Shaw and Allen 2000, Ramaiya et al. 2010, Medina et al. 2012; Heinrichs et al. 2010, 2011; Feldberg et al. 2007; Oguri et al. 2008), with some examples from the Neotropics (e.g. Heinrichs et al. 2009b; Feldberg et al. 2011; Dong et al. 2012) but only a few examples have been published to date for the southern hemisphere. One example of sympatric cryptic species having a centre of diversity in Australasia has been identified in the lichen *Cladia aggregata*, where 12 species were resolved (Parnmen et al. 2012).

In liverworts, lineage diversity suggestive of cryptic species in the Australasian *Lepidozia ulothrix* wasidentified by Cooper et al. (2011, 2012), and in another study the New Zealand *Lejeunea tumida* species group was found to be polyphyletic (Renner et al. 2010b, 2011). The additional example provided by *Radula buccinifera* suggests that crypsis and semi-crypsis are not just widespread, but are likely to be taxonomically and geographically pervasive within bryophytes, and the existence of sympatric cryptic entities may prove a common theme in the cryptogamic flora of the Australasian region.

There may be good reason for the persistent failure by traditional approaches to recognise instances of crypsis and semi-crypsis. Investigation of patterns of morphological variation within species belonging to the *Lejeunea tumida* species group found extensive overlap between species due to substantial intra-individual variation (Renner et al. 2013). Morphological variation that is within the developmental capacity of single genotypes, and even single meristems, can swamp inter-specific differences and mask differences between species, resulting in a variation white-out obscuring them to typological approaches. Furthermore, the degree of overlap between cryptic species can be independent of degree of relatedness. Crypsis can be an intrinsic by-product of the growth and development of liverwort gametophytes and cryptic species may be pervasive throughout the group. Lability in leaf morphology has been observed in the mosses Fontinalaceae (Shaw and Allen 2000), and *Tortula* (Mishler 1986, 1988), suggesting developmental plasticity in gametophyte morphology could contribute to crypsis in mosses.

Within the *Radula buccinifera* complex molecular data serve to emphasise the phylogenetic significance of the subtle morphological differences detected between lineages, differences that have either been overlooked, or dismissed via *ad hoc* and untested hypotheses of environmental or other intra-specific variation.

Variation and co-occurrence in sympatry complicate determination and may have contributed to the generally poor standard of identification in herbarium material. For this study 533 named specimens of *Radula buccinifera* (not including types) held by Australasian and overseas herbaria were examined, of which 190 (36 %) were actually *Radula buccinifera*. This implies a misidentification rate of 64%, even including specimens that have been determined for recent regional revisions and synopses. This includes specimens that are now referred to new species. Regardless of how a misidentification rate might be calculated to account for names available at the time of identification, the point is that most specimens identified as *Radula buccinifera* do not belong to that species. Perhaps encouragingly, most of the confusion is between *Radula buccinifera*, *Radula strangulata*, and *Radula demissa*, and the first two are very difficult to distinguish using morphology. However, *Radula demissa* differs in a number of qualitative and quantitative micro-morphological characters. The high rate of mis-identification has two consequences, firstly *Radula buccinifera* is misunderstood as a widespread and variable species, and secondly real phylogenetic diversity is overlooked. The set of misidentified specimens comprised 26 different *Radula* species (Table 3), including four of the new species described below, suggesting the working circumscription of *Radula buccinifera* was broad enough to include almost the entire Australasian *Radula* flora.

**Table 3.** Actual identities of 533 herbarium specimens determined as *Radula buccinifera*.

*Frullania* sp.	1
*Lejeunea* sp.	1
*Radula allisonii*	2
*Radula australiana*	11
*Radula buccinifera*	190
*Radula mittenii*	8
*Radula cuspidata*	1
*Radula demissa*	125
*Radula grandis*	1
*Radula helix*	2
*Radula jovetiana*	3
*Radula multiflora*	1
*Radula javanica*	2
*Radula novae-hollandiae*	12
*Radula notabilis*	1
*Radula plicata*	12
*Radula pugioniformis*	2
*Radula reflexa*	3
*Radula* sp. (a)	2
*Radula* sp. (k)	1
*Radula* sp. (p)	1
*Radula* sp. indet.	5
*Radula strangulata*	127
*Radula* subg. *Odontoradula*	10
*Radula tasmanica*	7
*Radula weymouthiana*	2
Total	533

### Biodiversity

The phylogenetic breadth of molecular phylogenetic investigations that have identified cryptic and semi-cryptic diversity, coupled with a mechanism explaining complicated, and often confusing, patterns of morphological variation make the extrapolation that all bryophyte groups contain overlooked diversity a fairly safe inference. Many studies result in reinstatement of synonyms (Heinrichs et al. 2006 and references therein; Heinrichs et al. 2009b, Medina et al. 2012) thereby reducing global synonymy rates, but many also detect genuinely new entities, eventually increasing the number of published and accepted names (e.g. Kreier et al. 2010). Both outcomes should result in upwardly revised estimates of global diversity. Within liverworts currently 7675 taxa are accepted (von Konrat et al. 2010a), including 227 *Radula* species (von Konrat et al. unpublished data).

Our study suggests that within *Radula buccinifera*, two synonyms need reinstating, and five new species need naming. This represents an increase of about 2% in global diversity in *Radula*, from study of a single ‘species’ in a relatively small southern region that does not have a reputation for diversity in this genus in comparison to, for example, the tropics. There may be other *Radula* species of convenience in paleotropical, neotropical, and oceanic regions. The precedent set by *Radula buccinifera* suggests that resolution of ‘snowball’ taxa could significantly increase estimates of global diversity for this genus.

The taxonomically pervasive, indeed indiscriminate, distribution of cryptic and semi-cryptic species suggests that estimates of global diversity for liverworts revised upward of 10,000 species might not be unreasonable. New Zealand, one of the regions involved in the *Radula buccinifera* complex, has the best studied liverwort flora in the southern hemisphere and is under ongoing investigation by a number of research groups, yet totally novel species are still being discovered (e.g. Glenny 1996; Renner et al. 2006, 2009; Renner 2010, Engel and Glenny 2008b, 2011; von Konrat et al. 2011, 2012; Glenny et al. 2009), synonyms within accepted species are being reinstated (Engel and Smith-Merrill 2010), taxon circumscriptions are being revised (von Konrat et al. 2010b) and through a combination of these and new geographic records (e.g. Glenny 1995; von Konrat et al. 2006; Renner and Pócs 2011) the liverwort flora is increasing at the rate of c. 5 species per annum (von Konrat et al. 2010a). If such increases are possible in a well-studied flora, the same is possible in other less well-studied parts of the world.

### Biogeography, dispersal and speciation

The identification of cryptic species, and reconstruction of spatial structure of genetic diversity informs biogeography and evolutionary ecology. In bryophytes, morphologically circumscribed species generally have larger distribution ranges than angiosperm species (Shaw 2001). This study presents another example where the broad morphological circumscription of a bryophyte species is untenable. To the best of our knowledge, *Radula buccinifera* does not occur in New Zealand. At first glance it may seem that the Australian and New Zealand floras have less in common following this revision. On the contrary the number of species shared between Australia and New Zealand doubles, due to the detection of *Radula strangulata* in Australia, and the trans-Tasman distribution of *Radula demissa*, described below.

Morphological similarity and continuity between *Radula buccinifera* and *Radula strangulata* makes inference of *Radula strangulata*’s distribution in Australia difficult, but at least two other specimens, both collected from rock under running water on the Australian mainland are morphologically and ecologically compatible with this species, suggesting it may be more widespread there. The apparent rarity of *Radula strangulata* in Australia, in contrast to its abundance in New Zealand, and the sequence similarity between the two regions are both consistent with recent east to west dispersal, though there may be other explanations. Other examples of east to west dispersal against the prevailing south-westerlies include two species of *Chionohebe* that dispersed independently from New Zealand to Australia, (Meudt and Bayly 2008), and two independent dispersals of *Asplenium hookerianum* that established populations in Victoria and Tasmania (Perrie et al. 2010).

In *Radula demissa* two reciprocally monophyletic geographic clades are also recovered in phylogenetic analysis (Figs 4, 5). Despite genetic divergence, there are no consistent morphological differences between individuals from New Zealand and Tasmania. It is possible that dispersal from Australia to New Zealand or vice versa was associated with a single dispersal event at a time when the species had a single chloroplast genotype and the genetic variation in the samples all post-dates that dispersal event. But our sampling, focussed as it is on the chloroplast genome, may have failed to detect descendants of other rare long distance spore-dispersal and events between the two regions by virtue of their low frequency, the fact that chloroplasts are probably maternally inherited, half the colonists will be male, half the offspring of female colonists will be male, and due to purely stochastic processes rare chloroplast haplotypes would be expected to go extinct within the colonised region (Hubbell 2001). Regardless, phylogeny suggests long distance dispersal and establishment between Tasmania and New Zealand is a rare event in *Radula demissa*.

Considerable lineage diversity was recovered within Australian *Radula australiana*. In contrast, diversity within New Zealand was more limited, with five of seven accessions forming a clade nested within Australian accessions. Populations established following dispersal should contain only a proportion of the variation in the parental populations (Templeton 1998), so this pattern is consistent with colonisation of New Zealand from an Australian source. *Radula australiana* is certainly easier to find on the Australian mainland than in New Zealand, and is associated with most rock outcrops (granite and basalt) in alpine regions. The apparent completeness with which *Radula australiana* has filled this habitat in Australia may explain its earlier detection there, than in New Zealand where it is widely but sparsely distributed in alpine areas, and is absent from apparently suitable sites. Formation of alpine habitats in New Zealand is believed to have occurred during the Pliocene (5–2.5 mya) (Cox and Findlay 1995; Batt et al. 2000; Winkworth et al. 2005), and dispersal to New Zealand is likely to be younger than this.

All entities within the *Radula buccinifera* complex are dioicous, which is the ancestral and most common condition in *Radula* (Devos et al. 2011a). Unlike cosexual and autogamous plants, at least two dispersal and establishment events in close proximity would be necessary to establish sexually reproductive populations in a new area. The capacity for dioicous bryophytes to form isolated mixed-sex populations was noted by Shaw (2000) from observations of *Mielichhoferia*. Aside from stochastic processes associated with spore dispersal and establishment, spore characteristics are relevant determinants of dispersability (van Zanten 1976, 1978; Gradstein et al. 1983). Tolerance of freezing might be crucial for maintaining viability during transit by highly unstable weather systems such as tropical cyclones, where violent internal convection cells expose spores to rapidly fluctuating temperatures, and not all species are tolerant of freezing (van Zanten 1978). For example, *Hymenodontopsis (Pyrrhobryum) mnioides* spores are poor stress tolerators, more so than the average species occurring in New Zealand and Tasmania and this possibly explains divergence between Australasia and Patagonia (van Zanten 1978; McDaniel and Shaw 2003). In contrast *Leptotheca gaudichaudii*, whose spores germinate following freezing (van Zanten 1978), has haplotypes shared between Australasia and Patagonia (McDaniel and Shaw 2003).

Despite its apparent rarity long-distance dispersal has contributed to diversity within the *Radula buccinifera* species-complex. One instance where a geographically isolated (Norfolk Island), morphologically distinctive individual is nested within an Australian clade was identified in this study, in *Radula buccinifera*. Rare and stochastic long-distance dispersal has been reported as contributing to diversification in *Leptoscyphus* (Vanderpoorten and Long 2006), by budding speciation where a population becomes spatially isolated and subsequently diverges, rendering the parental species paraphyletic (Vanderpoorten and Long 2006). The small size of the isolated population facilitates rapid drift and selection as parental alleles are lost at a faster rate than in parental populations. The molecular data for *Radula buccinifera* are consistent with an instance of budding speciation via dispersal from west to east across the Tasman Sea. The acquisition of gemmae by the Norfolk Island species is most likely an adaptive morphological response to challenges posed by its island habitat, i.e. dioicy and small population size resulting in limited opportunity for sexual reproduction. Highly modified gemmae are a common feature of many species within the tropical epiphyllous lineage of subg. *Metaradula*, e.g. *Radula protensa*, *Radula assamica*, *Radula epiphylla*, but gemmae are virtually unknown within other lineages of the subgenus.Asexual reproduction may be a key innovation underpinning rapid diversification within *Mitthyridium* a genus widespread across the Pacific (Wall 2005), and is a key adaptive trait to existence in a different kind of island habitat (leaves) in Lejeuneaceae (Kraichak 2012). Indeed, the idea that fast and efficient dispersal mechanisms at the local scale might be selected in volcanic island environments due to the abundance of empty niches has been recently suggested to explain the higher proportion of species producing specialized asexual diaspores on volcanic islands than on continents (Patiño et al. 2013b).

The traditional view that morphological evolution in bryophytes takes place over millions, if not tens of millions of years, has been confirmed in a couple of dated phylogenetic studies, including the moss *Hymenodontopsis* (as *Pyrrhobryum*) (McDaniel and Shaw 2003) and the liverwort *Leptoscyphus* (Devos and Vanderpoorten 2008). In contrast, the similarity in haplotypes between *Radula buccinifera* and the Norfolk Island species suggests more recent divergence, and more rapid morphological evolution. Age estimates for islands and habitats vary, and known fossils cannot be conclusively tied to nodes within the genus so dating the *Radula* phylogeny based on the current sample is not possible, Though *Radula anisotoma* on Norfolk Island has a maximum age of 7 Ma so could be used for calibrating the tree, but would need to be supplemented with other dates. Elucidating the time course underpinning processes resulting in contemporary phylogeographic patterns should be the focus of future study.

## Taxonomic treatment

Artificial key distinguishing species belonging to the *Radula buccinifera* aggregate. The first character presented is usually diagnostic. Other characters are included to 1) facilitate identification as far as possible, 2) identify couplet selection errors at subsequent steps of the key and 3) aid in the identification of species that are not included in this treatment, either because they are novel, or unrelated.

**Table:** 

1	Leaf-lobe cell surface roughened, verrucose. Lobules one quarter the lobe area on primary shoots, quadrate, with ampliate interior margin. Shoot systems regularly pinnate and subdimorphic with secondary shoots smaller than primary, and with more rectangular lobules whose antical margin may be reflexed near the stem insertion; plants from exposed situations may comprise mostly secondary shoots and the regularly pinnate branching pattern may not be apparent. Stems relatively massive 190–250 µm diameter, with cortical cells in a single tier of 30–50 rows; cell walls brown pigmented throughout; cortical cell walls heavily and continuously thickened, at times constricting the cell lumen; medulla cells in 80–110 rows, cell walls heavily thickened with coarse nodular trigones that become confluent, and constrict the cell lumen. Leaf insertion exceeding dorsal stem mid-line, insertion lines interlocking over two dorsal cortical cell rows, dorsal leaf-free strip absent. Perianths with low basal stem perigynium. Plants milky yellow-green when fresh	*Radula mittenii*
–	Leaf-lobe cell surface smooth, either unornamented or with low dome-shaped papillae. Lobules one eighth to one quarter the lobe area, shape on primary shoots various including rhombiform, tullate, quadrate and oblong with or without an ampliate interior margin. Shoot systems regularly pinnate with subdimorphic branching, or irregular with pseudodichotomous branches in association with gynoecia. Stems not massive, c. 100–200 µm diameter with cortical cells in a single tier of up to 35 rows, cell wall pigmentation various, unpigmented throughout, brown-pigmented in cortical cell walls only or brown pigmented throughout, cell wall thickening various, secondary thickening generally absent from medulla cell walls except *Radula pugioniformis*. Leaf insertion attaining the dorsal stem mid-line or not, never interlocking over two dorsal cortical cell rows, dorsal leaf-free strip present or absent. Perianths with a high basal stem perigynium. Plant colour various when fresh, including mid-green, glaucous-green, brown-green, or black-green	2
2	Female bracts in one and a half or two pairs. Lobules rhombic to trullate, inner lobule margin free for up to two thirds its length, free portion not ampliate, not extending across stem beyond insertion line, apex narrowly rounded to acute, free exterior margin straight, occasionally with a small knee above the lobe-lobule junction, margins entire; leaf-lobes weakly falcate. Stem anatomy with all cortical cell walls heavily and almost continuously thickened and brown pigmented, medulla walls with yellow-brown to brown pigmented secondary thickenings and nodular trigones that are confluent across medial walls	*Radula pugioniformis*
–	Female bracts in one pair. Lobules various, rhombic, quadrate, longitudinally rectangular; inner lobule margin free for up to one half its length, free portion ampliate or not, often extending across stem beyond insertion line, apex various, obtuse to acute, free exterior margin straight or curved, knee present or not, margins entire to crenulate; leaf-lobes not falcate to falcate. Stem anatomy with external cortical cell wall continuously thickened and brown pigmented, internal cortical cell walls unthickened or discontinuously thickened, unpigmented or with less intense pigmentation, medulla walls without pronounced secondary thickening and unpigmented (but brown pigmented in *Radula buccinifera*), or with discrete bulging trigones not confluent across medial cell walls and yellow-brown to brown pigmented	3
3	Perianth mouth flared; shoot systems pseudodichotomously branched. Medulla cells of stem with bulging trigones at cell junctions. Leaf-lobules trapeziform when well developed with exterior and interior margins nearly parallel, margins crenulate; Female bracts relatively small, subisolobous and closely overlapping	*Radula notabilis*
–	Perianth mouth not flared; shoot systems pinnately branched, with additional pseudodichotomous branches in female individuals. Medulla cells of stem without bulging trigones at cell junctions. Leaf-lobules rhomboid to quadrate, margins entire or crenulate. Female bracts various, not subisolobous, closely overlapping or not	4
4	Dorsal leaf-free strip present. Leaf lobes tending to lay in plane with the stem (not always the case) and the stem usually visible between the leaf lobes in dorsal view.	5
–	Dorsal leaf-free strip absent. Leaf lobes tending to be obliquely patent and lay over the stem, obscuring the stem surface in dorsal view	7
5	Leaf-lobes oblong-elliptic, with a straight postical margin held perpendicular to the stem. Leaf lobes fragmenting on mature shoot sectors. Female bract lobes oblong-elliptic, widely divergent. Leaf-lobules rhombic, with apex lying close to the stem margin	*Radula anisotoma*
–	Leaf-lobes rotund to ovate, with a curved postical margin. Leaf lobes not fragmenting. Female bract lobes elliptic-ovate, overlapping. Leaf lobules rhombic to quadrate, with apex lying close to the stem margin or away from it	6
6	Lobules quadrate to rhombic when small and large, one eighth to one sixth the lobe area; keel apex and postical lobe margin with shallow notch; interior lobule margin free for one third its length, free portion weakly ampliate in small stature lobules to moderately ampliate on large stature lobules, extending at most half way across the ventral stem surface; acroscopic margin S-shaped (typical in situ) to straight (when flattened), apical portion inclined toward stem, not exceeding (lying antical to) the lobule apex; apex obtuse to acute; free exterior margin straight curved, occasionally with a small knee above the lobe-lobule junction; margins plane, entire or shallowly repand; lobe-lobule junction slightly antical to, or level with, the acroscopic end of stem insertion	*Radula buccinifera*
–	Lobules quadrate when small to oblong, one twelfth to one sixth the lobe area, keel apex and postical lobe margin flush; interior lobule margin free for one fifth to one quarter its length, free portion not ampliate in small stature lobules to moderately ampliate on large lobules, extending at most half way across the ventral stem surface; acroscopic margin S-shaped, apical portion perpendicular to stem, in large lobules exceeding (lying antical to) the lobule apex; apex obtuse to apiculate; free exterior margin straight, margins plane; lobe-lobule junction well postical to the acroscopic end of stem insertion	*Radula strangulata*
7	Lobules quadrate, one quarter the lobe area, apex acute, interior margin free for one quarter to one third its length, ampliate over stem margin; keel curved, running seamlessly into leaf-lobe outline, lobe margins crenulate due to differential thickenings on medial external cell walls	*Radula australiana*
–	Lobules rhombic, one sixth the lobe area, apex obtuse, interior margin free for one fifth to one third its length, ampliate over the stem margin or not; keel curved, not running seamlessly into leaf-lobe outline, leaf-lobes weakly to strongly falcate, lobe margins crenulate due to differential thickenings on medial external walls or by bulging cells	8
8	Leaf-lobe cell surfaces unornamented, lobe margins crenulate due to bulging cells. Leaf lobes falcate	*Radula demissa*
–	Leaf-lobe cell surfaces with a single low dome-shaped papilla over each cell, lobe margins crenulate due to differential thickenings on medial external cell walls. Leaf lobes at most weakly falcate	*Radula imposita*

### Radula anisotoma

M.A.M.Renner sp. nov.

http://species-id.net/wiki/Radula_anisotoma

Figs 6 –7

#### Type:

Australia: Norfolk Island: Mount Pitt Reserve, Filmy Fern Trail, off Selwyn Pine Road, 29°01'S, 167°58'E, 130 m, 3 Dec 1984, *H. Streimann 32084A*, (holotype: CANB650459).

#### Diagnosis.

Within the *Radula buccinifera* complex *Radula anisotoma* is most similar to *Radula strangulata* by virtue of its small, rhomboid lobules whose apex lies close to or over the stem, and its leaf-lobes not interlocking over the dorsal stem surface, such that the stem is visible between leaves in dorsal view, and its habit of growing on rocks in association with waterways,but differs by its oblong leaf-lobes that are caducous, fragmenting into several irregular pieces, its narrower and longer female bract lobes, rhombic to trullate lobules and smaller stature.

#### Description.

[From CANB650459] Forming diffuse patches of small shoots, or mixed with other bryophytes, brown in herbarium; shoot systems monomorphic, irregularly branched, *Lejeunea*- type branching frequent, with additional pseudodichotomous branching due to production of subfloral innovations below gynoecia; 950–1280 mm wide and up to 20 mm long, branches initially smaller in stature than parent shoot, attaining similar stature to parent shoot after two or three leaf pairs; older shoot sectors denuded of leaf-lobes. Stems 90–150 µm diameter, with cortical cells in a single tier of 15–22 rows; cortical cell walls brown-pigmented; external free cortical cell wall continuously thickened, radial longitudinal cortical walls thin or slightly thickened, inner tangential walls discontinuously thickened; medulla cells in 12–20 rows, cell walls faintly yellow-pigmented or colourless, with small to medium-sized triangular trigones, walls between trigones unthickened. Cortical cells on dorsal stem surface arranged in straight longitudinal rows on young and mature shoot sectors. Leaf insertion not reaching dorsal stem mid-line, leaving one or two dorsal cortical cell rows leaf-free, dorsal leaf-free strip present; leaf insertion not attaining the ventral stem mid-line, leaving two ventral cortical cell rows leaf-free. Leaf lobes oblong-elliptic, 380–710 µm long by 300–490 μm wide, contiguous, not falcate, acroscopic base lying in plane with stem, plane, not interlocking over the dorsal stem surface, stem visible between leaf lobes in dorsal view; margins irregularly repand, marginal cells bulging, the interior lobe margin not or only weakly ampliate, not or hardly riding onto dorsal stem surface, antical margin curved, exterior margin sharply curved through nearly 100°, postical margin straight; angle between postical lobe margin and keel c. 135°. Lobules rhomboid, remote, one tenth to one eighth the lobe area, 140–350 µm long by 105–240 μm wide; keel straight or rarely slightly arched, angle between keel and stem 135°, keel apex and postical lobe margin flush; interior lobule margin free for one third to one half its length, free portion not or weakly ampliate, hardly riding onto ventral stem surface, not concealing the stem in ventral view; acroscopic margin straight or curved; inclined inwards toward the stem; apex acute, laying close to or over the stem margin, free exterior margin straight to weakly curved, margins irregular; lobe-lobule junction level with or slightly postical to the acroscopic end of stem insertion; attached to stem along 0.5–0.33 of the interior margin, stem insertion more or less linear, gently curved at acroscopic and basiscopic ends, not revolute; lobule apex bearing a single papilla, with another two papilla situated on the interior lobule margin above the stem insertion. Leaf lobe cells rounded, not arranged in rows, unequally sized, 9–24 µm long by 9–19 μm wide, thin walled with concave trigones, medial wall thickenings absent; cells of lobe margin smaller than those of leaf middle, quadrate to rectangular, 9–14 µm long and wide, interior and exterior cell walls not differentially thickened; leaf lobe cell surface smooth. Oil-bodies not known. Asexual reproduction by caducous leaf lobes, fragmenting into several irregular pieces, marginal lobe cells often proliferating to form bud-like shoot primordial. Dioicous. Androecia not known. Gynoecia terminal on branch shoots, subtended by 2 or 3 subfloral innovations that are the same size as the branch shoot and are again fertile; archegonia 115–130 µm tall, archegonial neck 6 cell columns; 14–15 per gynoecium on a small disc of tissue, encompassed by the protoperianth; female bracts in one or one and a half pairs, symmetrical, imbricate, narrow oblong-elliptic, lobe 655–975 μm long by 265–450 μm wide, margins entire or repand; lobules rhomboid to trullate, one fifth to one quarter the lobe area, apex obtuse to acute, not or shallowly notched, keel arched, margins irregular; bract insertion lines interlocking dorsally and ventrally, insertion equitant. Perianths c. 3100 µm long and 660 µm wide at mouth, mouth repand, more or less parallel sided for upper third, then tapering to tubular stem perigynium comprising the lower third to half, broadest at mouth, walls 2- or 3-stratose at junction with perigynium, unistratose above. Long stem perigynium present, multi-stratose throughout. Calyptral perigynium present.

**Figure 6.**
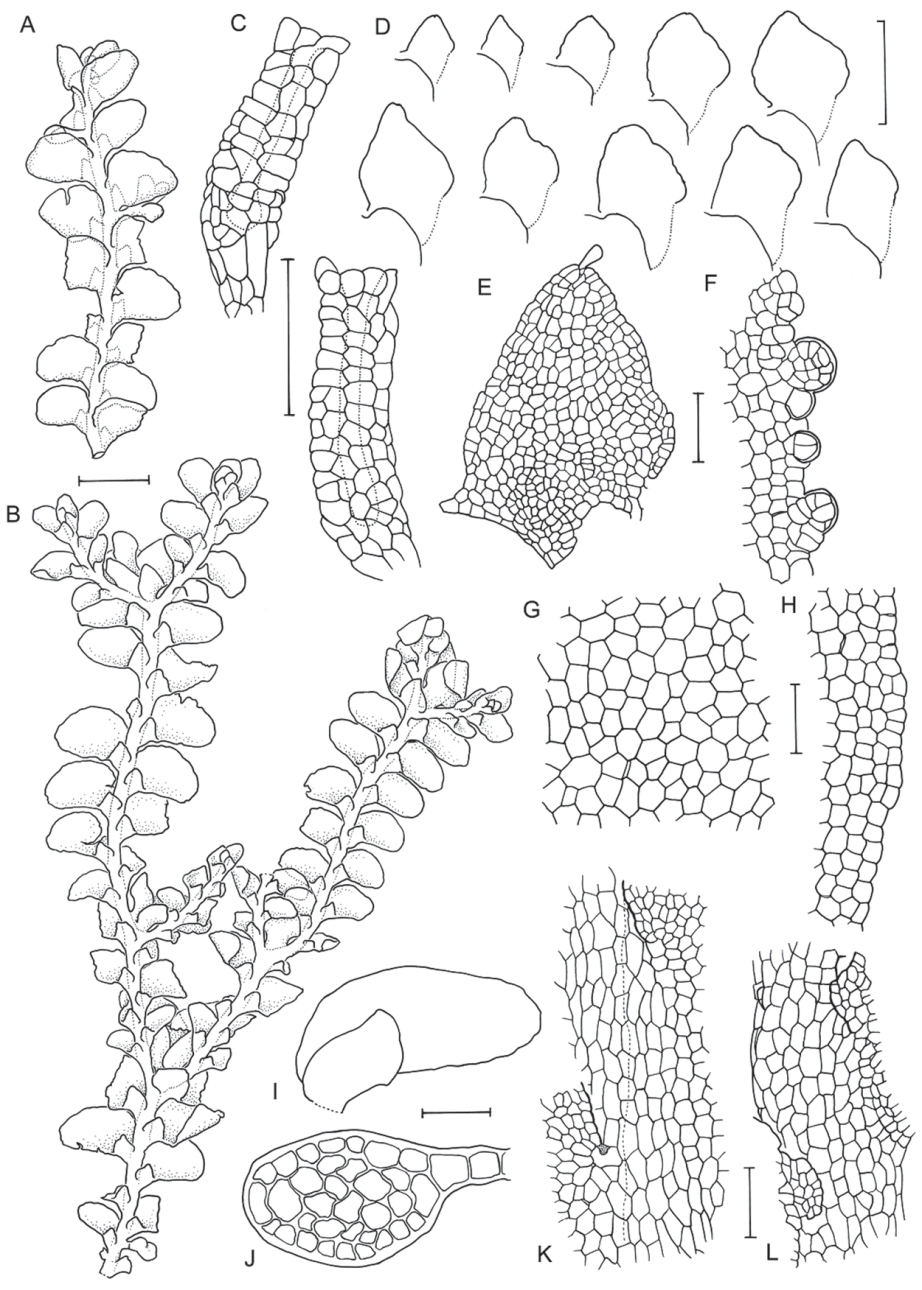
*Radula anisotoma* line drawings **A** Dorsal shoot **B** Ventral shoot **C** Archegonia **D** Ten haphazardly selected lobules, showing size and shape variation **E** Lobule showing cellular detail **F** Cells of leaf lobe margin showing initiation of shoot primordia **G** Medial leaf-lobe cells **H** Marginal leaf-lobe cells **I** Female bract showing narrow elliptical lobe **J** Stem transverse section **K** Dorsal stem surface showing leaf-free dorsal cortical cell rows **L** Ventral stem surface. Scale bars: **A–B**: 600 µm, **C, F**: 40 µm, **D, I**: 240 µm, **E, G, H, J–L**: 60 µm. All from CANB650459.

**Figure 7.**
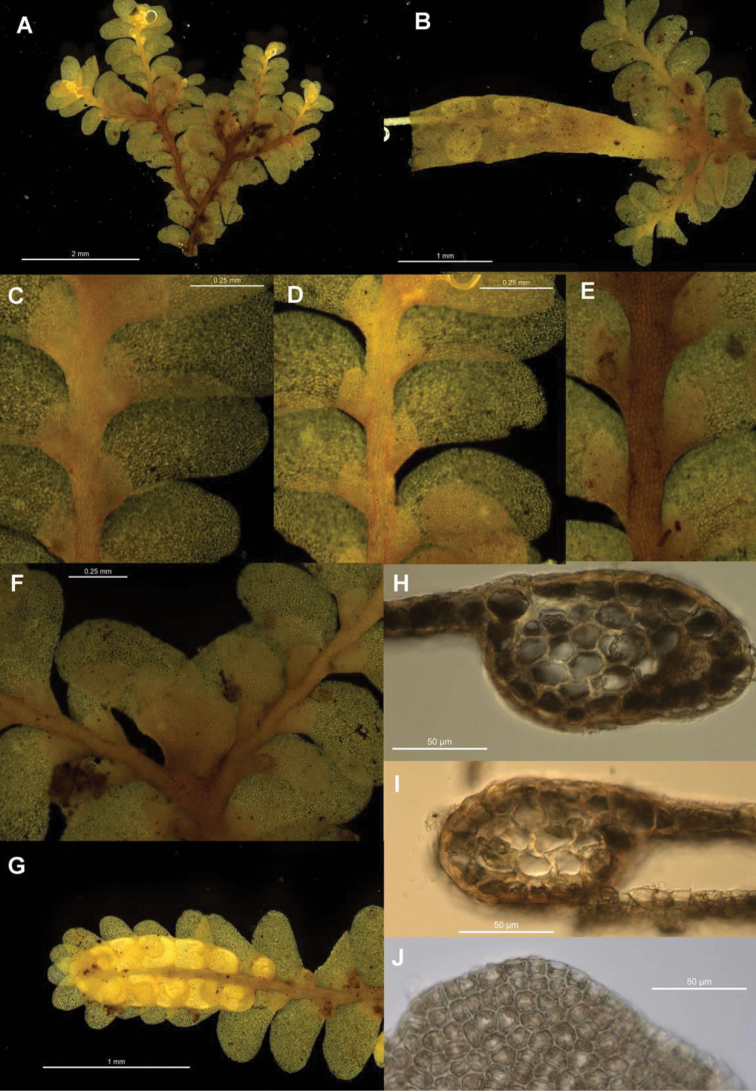
*Radula anisotoma* pictures **A** Ventral view of shoot **B** Mature perianth. **C–E** Ventral view of shoots and lobules **F** Gynoecium **G** Androecium **H–I** Transverse sections of stems from primary shoot **J** Leaf-lobe marginal cells. All from CANB650459.

#### Etymology.

From Greek *an* (αν-): not, *isos* (ισοσ): even, *tomos* (τοµος, m.): slice, piece - uneven slice, in reference to the caducous leaf lobes that fragment into uneven pieces.

#### Distribution and ecology.

*Radula anisotoma* is currently known only from Norfolk Island where, at the only known location, it occurred in dense forest at 130 m within Mt Pitt Reserve. The plants grew beside a creek, presumably within a gully, on a rock admixed with *Lejeunea anisophylla* Mont., *Metzgeria* sp. and *Radula* cf. *novae-hollandiae* (see comments below under *Radula farmeri* Pearson for explanation).

#### Recognition.

Identification of *Radula anisotoma* should present no difficulty. The most accessible morphological character of *Radula anisotoma* that differs from all other members of the *Radula buccinifera* complex is the production of caducous leaves and associated proliferation of marginal lobe cells to form bud-like shoot primordial. Caducous leaves are not produced by any other member of the *Radula buccinifera* complex, with the exception of *Radula mittenii* which differs in a number of macro- and micro-morphological characters, and bud-like shoot primordia have only been observed twice, on two different specimens of *Radula strangulata* (M.A.M. Renner pers. obs.).

*Radula anisotoma* is most similar to *Radula strangulata* and, notwithstanding the differences described above, could be confused with that species. However, there are several subtle differences between *Radula anisotoma* and *Radula strangulata* in the shape of leaf lobes, lobules, and female bracts, which will aid identification. The leaf lobes of *Radula anisotoma* are oblong, whereas they are round in *Radula strangulata*. The lobules of *Radula anisotoma* are rhombic to trullate, whereas they are rhombic to longitudinally rectangular in *Radula strangulata*. The female bract lobes are narrowly oblong in *Radula anisotoma*, whereas they are elliptic-ovate in *Radula strangulata*. Finally, *Radula anisotoma* is generally a smaller plant than *Radula strangulata*. Comparison with known material is recommended in order to appreciate the degree of difference in shape between, as well as variation within, each species when making determinations.

#### Specimens examined.

Australia: Norfolk Island: Mount Pitt Reserve, Filmy Fern Trail, off Selwyn Pine Road, 29°1.3'S, 167°57.6'E, 130 m, 3 Dec 1984, *H. Streimann 32078* (CANB650457, NICH, NY, EGR, H); ibid. *H. Streimann 32083*, CANB650458.

### Radula australiana

K.Yamada Journal of the Hattori Botanical Laboratory 51: 323. 1982.

http://species-id.net/wiki/Radula_australiana

Figs 8 –10

#### Type.

Australia: New South Wales: Merrits Creek 3 km east of Mt. Kosciuszko, 1870 m, 9 Feb 1978, *H. Streimann 5358A* (holotype: NICH, isotype: CANB!).

#### Description.

Forming pure turfs or mats of shoots, dark brown in herbarium; shoot systems regularly pinnately branched, with additional pseudodichotomous branching in female plants due to production of pairs of subfloral innovations below gynoecia; dimorphic, primary shoots 1.5–1.8 mm wide and up to 40 mm long, secondary shoots smaller in stature than parent shoot, 0.8–1.0 mm wide, and either apparently terminating growth after 4 to 7 leaf pairs, or producing reproductive structures and, in female plants, continuing vegetative growth; older shoot sectors retaining leaf-lobes.

Stems 120–160 µm diameter, with cortical cells in a single tier of 23–29 rows, cortical cell walls yellow-brown pigmented, external free cortical cell wall continuously thickened, radial longitudinal cortical walls thin or slightly thickened, inner tangential walls thickened; medulla cells in 23–45 rows, medulla cell walls faintly yellow-pigmented, thin walled, small triangular trigones, medial walls unthickened. Cortical cells on dorsal stem surface arranged in straight longitudinal rows on young and mature shoot sectors. Leaf insertion reaching dorsal stem mid-line, leaving no dorsal cortical cell rows leaf-free; leaf insertion not attaining the ventral stem mid-line, leaving two ventral cortical cell rows leaf-free. Leaf lobes rotund, 475–920 µm long by 400–780 μm wide, contiguous, not falcate, acroscopic base not sharply deflexed away from stem, weakly concave, not or weakly interlocking over the dorsal stem surface, stem visible between leaf lobes in dorsal view; margins entire or crenulated, not repand, the interior lobe margin shallowly ampliate, reaching the opposite stem margin, antical and exterior margins more or less continuously curved, postical margin shallowly curved or straight; angle between postical lobe margin and keel 140–180°. Lobules quadrate on leading shoots, one sixth to one quarter the lobe area, 330-605 µm long by 370–595 μm wide; keel straight to shallowly curved, angle between keel and stem 100–135°, keel turning through up to 30°, keel apex and postical lobe margin flush; interior lobule margin free for one quarter to one third its length, free portion ampliate, extending half way across the ventral stem surface or more; acroscopic margin S-shaped to straight, apical portion slightly inclined toward stem or perpendicular to it; apex obtuse but usually weakly apiculate; free exterior margin straight, margins plane, entire; lobe-lobule junction level with or slightly postical to the acroscopic end of stem insertion; attached to stem along 0.66–0.75 of the interior margin, stem insertion gently curved, not revolute; lobule apex bearing a single papilla, another two papilla situated on the interior lobule margin above the stem insertion. Leaf lobe cells rounded-oblong, not arranged in rows, unequally sized, 13–35 µm long by 11–21 μm wide, thin-walled with small triangular trigones, medial wall thickenings absent; cells of lobe margin smaller than those of leaf middle, quadrate to rectangular, 11–18 µm long by 9–13 µm wide, interior walls moderately and continuously thickened, exterior wall moderately and differentially thickened at mid-wall, forming a conspicuous bulge and imparting a crenulate appearance to lobe margin; leaf lobe cell surface unornamented, smooth. Oil-bodies 2 or 3, light brown, granular, internally homogeneous, filling the cell lumen. Asexual reproduction absent. Dioicous. Androecia on branches that usually terminate after production of 4 or 5 pairs of antheridial bracts, but rarely branches indeterminate, bearing ∞ pairs of antheridial bracts; lobules epistatic, keel deeply curved, bucket-like, free apical portion triangular, apex acute, interior margin ampliate, covering ventral stem surface, and imbricate with adjacent antheridial lobules; lobes rounded, not caducous, antheridia not seen. Gynoecia terminal on branch shoots, subtended by two or three subfloral innovations that are full-sized and again fertile; archegonia 125–150 µm tall, archegonia neck five cell columns, 10 per gynoecium on a small disc of tissue, encompassed by a low protoperianth; female bracts in one pair, symmetrical, tightly imbricate, elliptic-obovate, weakly falcate, lobe 690–770 μm long by 430–535 μm wide, margins crenulate; lobules rectangular, one half to two thirds the lobe area, apex obtuse, keel straight to arched, margins crenulate; bract insertion lines interlocking dorsally and ventrally, insertion equitant. Perianths 4200–4700 µm long and 1050–1200 µm wide at mouth, mouth entire to irregular, parallel sided for upper two thirds, widening to flask shaped, faint bulb in basal third, broadest in middle of this bulb, 1200–1350 µm wide, then tapering to base. Perianth walls unistratose above, with bistratose bands extending up to half way up perianth, increasing in width toward base, becoming confluent, basal perianth walls progressively increasing in thickness, 2–3-stratose. Long stem perigynium present, 5-6 stratose, cell walls heavily thickened and brown-pigmented. Calyptral perigynium present, base of calyptra 2–4 stratose at base, strata progressively lost, unistratose above, unfertilised archegonia elevated on surface of calyptra.

**Figure 8.**
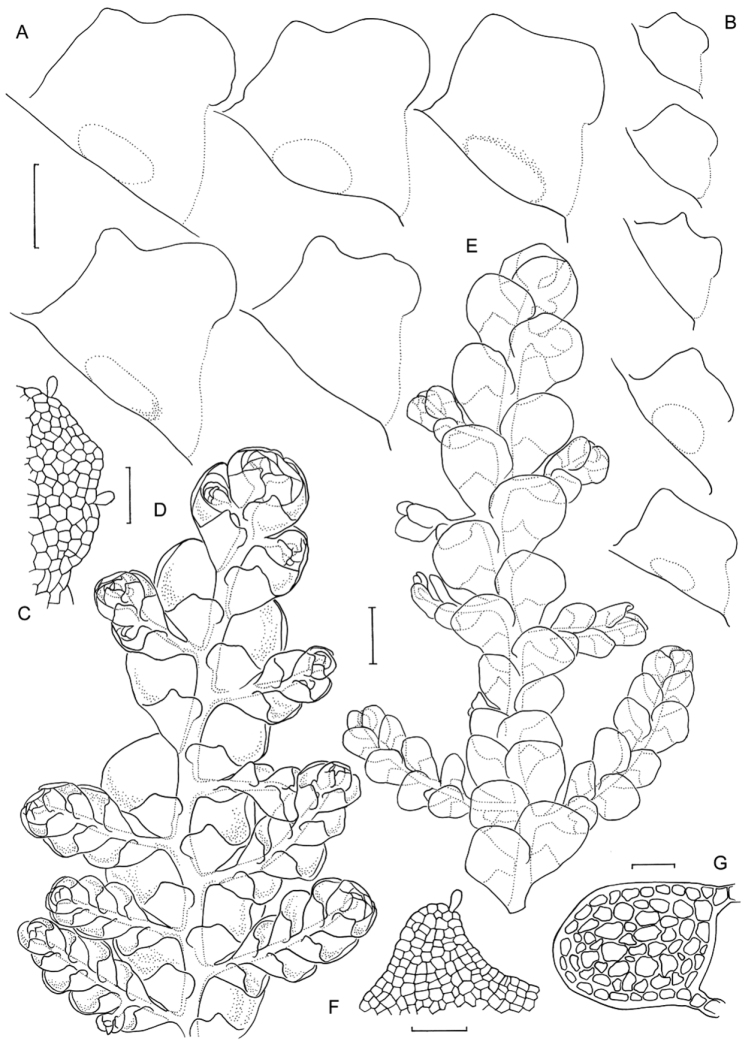
*Radula australiana* line drawings 1: **A** Five lobules from primary shoots showing size and shape variation, with broadly ampliate free interior margin and drawn-out apex **B** Five lobules from secondary shoots showing size and shape variation **C** Cellular detail of interior free lobule margin **D** Ventral shoot **E** Dorsal shoot **F** Cellular detail of lobule apex **G** Transverse stem section from primary shoot. Scale bars: **A–B**: 240 µm, **D–E**: 600 µm, **C, F**: 60 µm, **G**: 40 µm. All from NSW273911.

**Figure 9.**
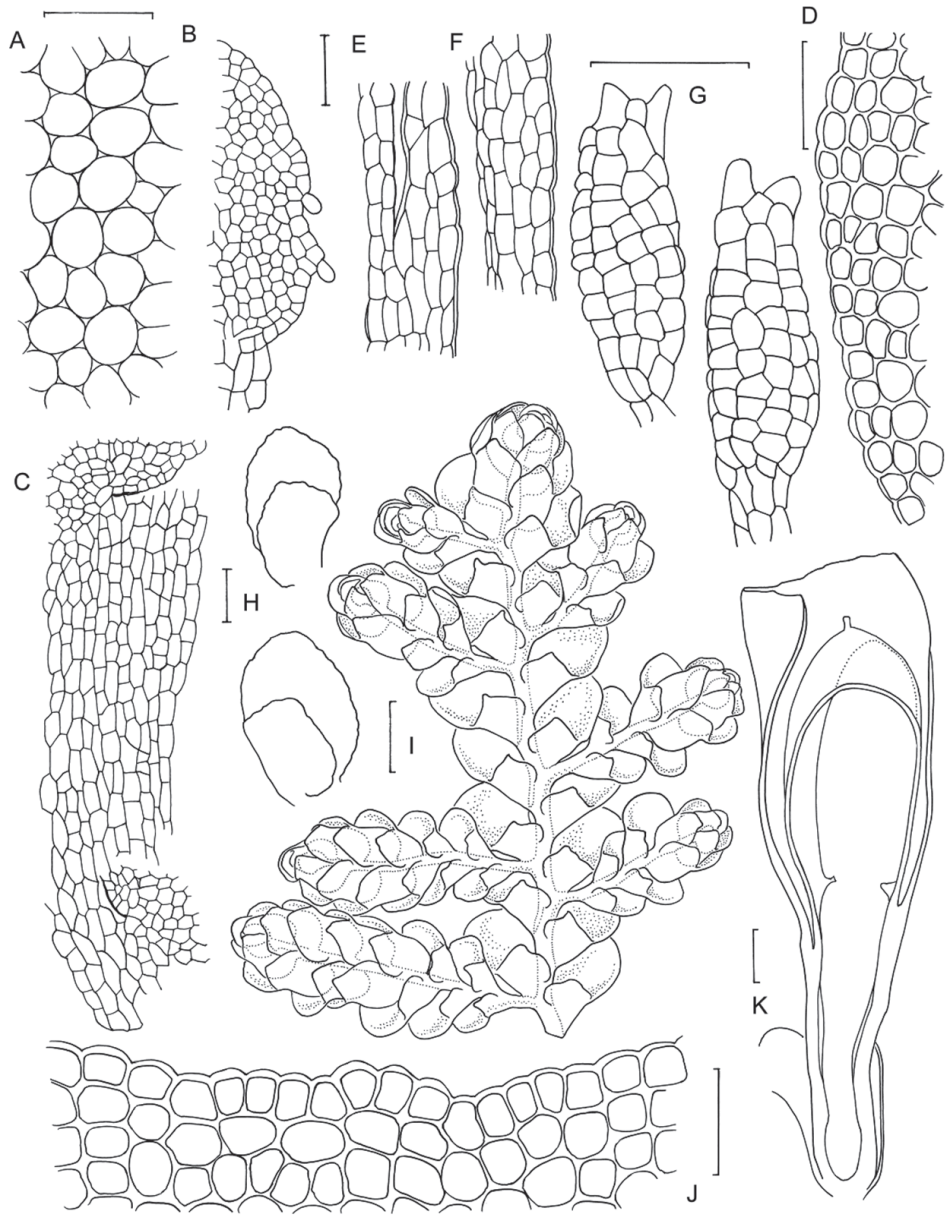
*Radula australiana* line drawings 2: **A** Medial leaf-lobe cells **B** Cellular detail of of interior free lobule margin **C** Dorsal stem surface showing leaf insertion lines meeting at the dorsal stem mid-line, leaving no dorsal leaf-free strip **D** Marginal leaf-lobe cells **E** Cellular detail of junction between stem perigynium, perianth wall, (at right) and calyptral perigynium (at left) **F** Cellular detail of stem perigynium wall **G** Archegonia **H** Female bracts **I** Ventral view of male shoot **J** Cellular detail of perianth mouth **K** Longitudinal section of perianth. Scale bars: **I**: 600 µm, **A, D, J**: 40 µm, **H, K**: 240 µm, **E–G**, 60 µm. **A–D** from NSW273911, **I** from MEL2300398, **F–H, J–K** from NSW272906.

**Figure 10.**
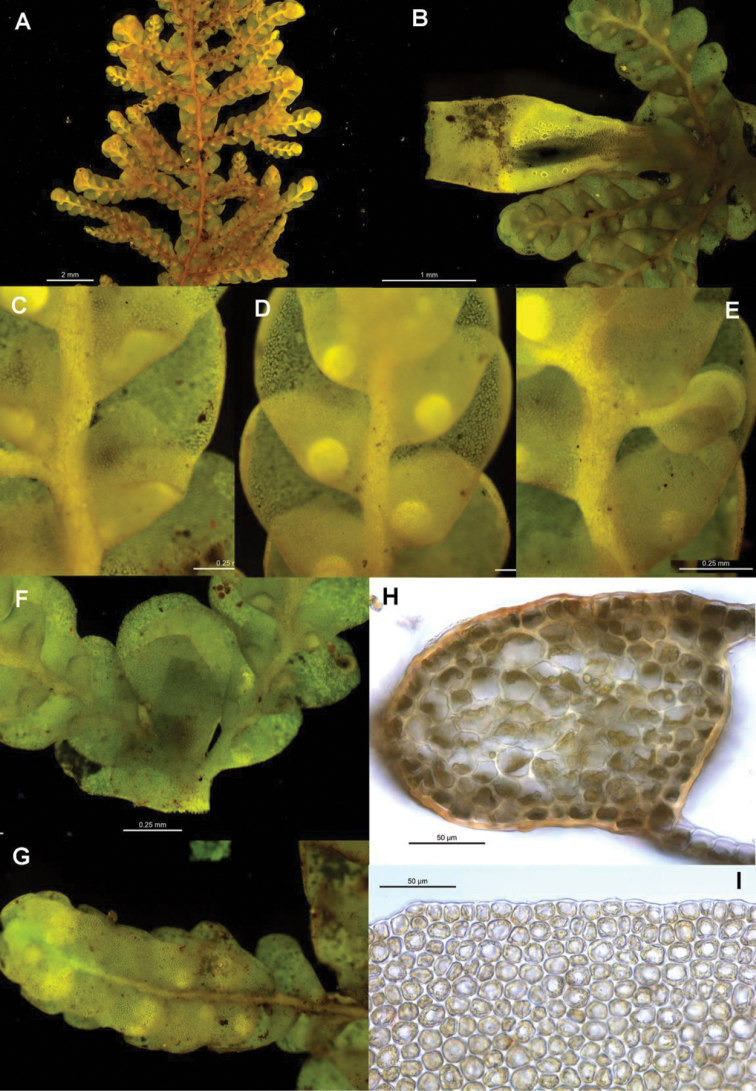
*Radula australiana* pictures. **A** Ventral view of shoot **B** Immature perianth. **C–E** Ventral view of shoots and lobules **F** Gynoecium **G** Androecium **H** Transverse sections of stems from primary shoot **I** Leaf-lobe marginal cells. **A** from CHR559976, all others from NSW875862.

#### Etymology.

Australian.

#### Distribution and ecology.

*Radula australiana* occurs on mainland Australia (NSW, ACT, VIC), and in Tasmania and New Zealand, usually well above treeline in alpine or subalpine shrublands, grasslands and tussocklands where it grows in association with seepages and running water over and around exposed bedrock and boulders on alpine bluffs, rock outcrops and rock piles. However, *Radula australiana* also inhabits rocky open sites within forest habitats, particularly in Australia where high altitude *Eucalyptus* forest occurs in the alpine zone, and on bluffs associated with watercourses in New Zealand montane beech forest. *Radula australiana* is primarily a lithophyte on a wide range of sedimentary, metamorphic, and igneous rocks including greywacke and schist in New Zealand, and granite and basalt in Australia. Microsites occupied by *Radula australiana* are typically sheltered and shaded, such as the back walls of recesses in rock bluffs, crevices, between boulders within ephemeral streambeds. *Radula australiana* may also occur in bryophyte turfs on soil, usually in the shade of surrounding woody vegetation or rock. It shares all of these habitats with *Radula helix*, *Herzogobryum teres*, *Nothogymnomitrion erosum*, *Cheilolejeunea mimosa*, and *Andreaea* spp.

#### Variation.

Individuals vary in branching density, New Zealand plants are typically densely branched, and this also occurs in Australia. Openly branched individuals typically have larger shoots and correspondingly larger lobules that produce more pronounced acuminate lobule apices. These differences may be associated with both patch age and microsite. Shoots colonizing naked rock are always openly branched. Those growing in bryophyte turfs on soil are often closely branched.

#### Recognition.

One of the first clues to the identity comes from the habitat and microhabitat occupied by *Radula australiana*, as it is one of the few Australasian *Radula* species that inhabits subalpine and alpine areas, frequently in association with exposed rock. Among alpine species it is one of two having brown pigments, the other being *Radula demissa* M.A.M.Renner. Despite the considerable morphological disparity between them, three unrelated species have been confused with *Radula australiana*. *Radula aneurismalis* is yellow-green or orange-green, has a single botryoidal, light brown oil-body per cell, possesses microphyllous branches, has a distinct narrowly inflated lobule carinal region that extends the length of the keel, produces androecia on short spike-like lateral branches that may be determinate, and the perianths lack a stem perigynium. In contrast *Radula australiana* is brown-green, has two or three granular, brown oil-bodies per cell, does not produce microphyllous branches, though lateral branches may be smaller in stature than primary shoots, has a broadly inflated carinal region, produces androecia on long branches and on primary axes, and has perianths with a long stem perigynium.

*Radula helix* is almost as different from *Radula australiana* as is *Radula aneurismalis*. *Radula helix* is yellow-green, has 3–5 smooth, hyaline, oil-bodies per cell, is paroicous, with androecia immediately below gynoecia, and the perianth lacks a stem perigynium. In contrast, *Radula australiana* is brown-green, has 2–3 granular, brown oil-bodies per cell, is dioicous, and the perianth has a stem-perigynium.

*Radula acutiloba* is similar in its lobules having an acuminate tip on main shoots, and on this basis *Radula australiana* was confused with *Radula acutiloba* in Devos et al. (2011) by the first author of this contribution who followed herbarium determinations. Though sharing similar lobule shapes, and some phenetic similarity, *Radula australiana* differs from *Radula acutiloba* in lacking subdiscoidal gemmae on the leaf lobe margin, by its brown-green not yellow-green colour, by the presence of two or three light to tan-brown oil bodies in each leaf lobe cell, lack of secondary thickening on cell walls in the stem medulla, and by its occurrence in subalpine and alpine habitats. In all populations of *Radula acutiloba* examined, subdiscoidal gemmae have been present on the leaf lobe margin. This feature alone is sufficient to discriminate between these two species.

Two related species, also members of the *Radula buccinifera* complex, occasionally co-occur with *Radula australiana* and may be confused with it. In Australia, *Radula buccinifera* is typically a forest inhabitant, but at some sites in Victoria and Tasmania where forest occurs over and among exposed granite boulders at high altitude, the ecological envelopes of *Radula australiana* and *Radula buccinifera* overlap, and they may co-occur. Morphological characters by which *Radula australiana* may be distinguished from *Radula buccinifera* are presented in the recognition section for *Radula buccinifera*.

In New Zealand, *Radula demissa* is typically a forest inhabitant, but has an alpine ecotype that, although it has not yet been found co-occurring with *Radula australiana*, occupies similar microhabitats. Morphological characters by which *Radula australiana* may be distinguished from *Radula demissa* are presented in the recognition section for *Radula demissa*. *Radula australiana* could be confused with *Radula strangulata*, for differences between these two species see the recognition section of *Radula strangulata*.

#### Specimens examined.

Australia: New South Wales: Southern Tablelands, Mount Kosciuszko National Park, Main Range track to Kosciuszko summit from Charlotte Pass, 36°27'01"S, 148°18'31"E, 1920 m, 26 Feb 2011, *M.A.M. Renner 5114 & E.A. Brown*, NSW893115; Southern Tablelands, Mount Kosciuszko National Park, Main Range track to Kosciuszko summit from Charlotte Pass, 36°27'01"S, 148°18'31"E, 1920 m, 26 Feb 2011, *M.A.M. Renner 5115 & E.A. Brown*,NSW893116; Southern Tablelands, Kosciuszko National Park, unnamed hill north of Daners Creek and Pipers Creek junction, 36°22'35"S, 148°27'37"E, 1600 m, 27 Feb 2011, *M.A.M. Renner 5127 & E.A. Brown*, NSW909241; Southern Tablelands, Kosciuszko National Park, unnamed hill north of Daners Creek and Pipers Creek junction, 36°22'35"S, 148°27'37"E, 1600 m, 27 Feb 2011, *M.A.M. Renner 5129 & E.A. Brown*, NSW909251; Southern Tablelands, Kosciuszko National Park, unnamed hill north of Daners Creek and Pipers Creek junction, 36°22'18"S, 148°27'34"E, 1720 m, 27 Feb 2011, *M.A.M. Renner 5130 & E.A. Brown*, NSW909252;

Australian Capital Territory: 36 km SSW of Capital Hill, Canberra, Tower 2.5 km north of Orroral Tracking Station, 35°37'S, 148°59'E, 1340 m, 22 Oct 1987, *H. Streimann 38961*, HO312250; New South Wales: Southern Tablelands, Kosciuszko National Park, The Rams Head Range, Merrits Spur, 36°29'S, 148°18'E, 1770 m, 13 Jan 2002, *J.A. Curnow 5638*, CANB636815; *J.A. Curnow 5635*, CANB636812; Kosciuszko National Park, 7.5 km NE of Mt Kosciuszko, Blue Lake, 36°24'S, 148°19'E, 2020 m, 3 Mar 1991, *H. Streimann 47093*, CANB9107074; Snowy Mountains, The Rams Head Range, Charlotte Pass, 36°26'S, 148°19'E, 1830 m, 3 Dec 1965, *L.G. Adams 1545*, CANB162786, CHR265733;

Victoria: Mt McKay, Alpine National Park, 16 km SSE of Mount Beauty, 36°52'S, 147°14'E, 1840 m, 18 Feb 1994, *H. Streimann 53505*, CANB9403675; *H.Streimann 53469a*, MEL2300398; Snowfields, Mount Buller, 37°07'S, 146°25'E, 1665 m, 9 March 1953, *J.H. Willis* *s.n.*, MEL1037780, as *Radula physoloba*; Mt Buller area, 37 km ESE of Mansfield, 37°09'S, 146°26'E, 1600 m, 30 December 1992, *H.Streimann 50758*, CANB9219759; Snowfields, Alpine National Park, Cope Creek, 36°54'S, 147°14'E, 1650 m, 5 March 1993, *E.A. Brown 93/72 & K.L. McClay*,NSW272960; Snowfields, Baw Baw National Park, Alpine Walking Track, c. 100 m north of Mount Erica car park, 37°53'S, 146°21'S, 1100 m, 9 March 1993, *E.A. Brown 93/145 & K.L. McClay*, NSW273911; Charlotte Pass, Ramshead Range, Snowy Mountains, c. 6,000 ft, 3 December 1965, *L.G. Adams 1545*, FH00284638; Snowfields, Mount Buller, summit, 37°08'41"S, 146°25'29"E, 1795 m, 28 Feb 2011, *M.A.M. Renner 5133 & E.A. Brown*, NSW875860; Snowfields, Mount Buller, summit, 37°08'41"S, 146°25'29"E, 1795 m, 28 Feb 2011, *M.A.M. Renner 5135 & E.A. Brown*, NSW875862; Snowfields, Mount Buffalo National Park, Slope immediately below carpark at The Horn, 36°46'33"S, 146°45'51"E, 1614 m, 01 Mar 2011, *M.A.M. Renner 5142 & E.A. Brown*, NSW875928; Snowfields, Mount Buffalo National Park, Mahomets Tomb outcrop, 36°45'12 S, 146°47'41"E, 1570 m, 01 Mar 2011, *M.A.M. Renner 5150 & E.A. Brown*, NSW875947; Snowfields, Alpine National Park, Mount Loch, summit, 36°57'30"S, 147°09'19"E, 1858 m, 2 Mar 2011, *M.A.M. Renner 5162 & E.A. Brown*, NSW875951; East Gippsland, Errinundra National Park, Mount Ellery, track to summit from the Ferntree track, 37°23'38"S, 148°46'21"E, 1080 m, 4 Mar 2011, *M.A.M. Renner 5204 & E.A. Brown*, NSW909259;

Tasmania: Ben Lomond, Hamilton Crags, 42°32'S, 147°41'E, 1460 m, 5 Jan 1992, *A. Moscal 22365*, HO301803; Mt. Field, Mt. Field East, 42°40'S, 146°39'E, 1155 m, 12 Apr 1992, *A. Moscal 23324*, HO132882; Mt. Field, Naturalist Peak, 42°60'S, 146°31'E, 1390 m, 10 Mar 1992, *A. Moscal 23011*, HO525812;

New Zealand: South Island: Nelson, Kahurangi National Park, Cobb Valley, between Cobb Lake and Round Lake, 41°03'23"S, 172°30'08"E, 1150 m, 19 Feb 2012, *M.A.M. Renner 6230*, NSW895690; Nelson, Kahurangi National Park, Cobb Valley, Round Lake cirque, 41°03'21"S, 172°29'53"E, 1290 m, 19 Feb 2012, *M.A.M. Renner 6239*, NSW896176; Hawkdon Ecological Region, Arthurs Pass Ecological District, Arthurs Pass, Temple Basin, 42°55'S, 171°35'E, 1440 m, 1 Apr 2001, *M.A.M. Renner 01/114*, AK280485; Canterbury Land District, Ohau, South Huxley Valley, 44°4'S, 169°42'E, 1200 m, 9 Nov 1996, *D. Glenny 6567*, CHR559967; Canterbury Land District, Ohau, South Huxley Valley, 44°02'S, 169°46'E, 1530 m, 9 Nov 1996, *D. Glenny 6575*, CHR559976; South Westland, northern Olivine Range, north of Dragon, 44°9'S, 168°37'E, 1250 m, 14 Feb 1995, *D. Glenny 5769b*, WELT-H0010890;Otago Land District, Remarkables Range, head of Wye Creek, 45°5'S, 168°50'E, 1760 m, 28 Dec 1998, *D. Glenny 7627*, CHR529388; East Dome, Garvie Range, 4500 ft, 5 Dec 1981, *J. Child*, CHR427233; Earnslaw, N ridge, 24 Oct 1973, *J. Child*, CHR427228; Fiordland, Fiordland National Park, Cozette Burn, 1080 m, 10 Apr 2002, *M.A.M. Renner* *s.n.*, CHR583911; South Westland, Haast Pass, western slopes of Mount Armstrong above Brewster Hut, 44°05'22"S, 169°24'55"E, 1530 m, 16 Feb 2012, *M.A.M. Renner 6142*, NSW895444; ibid, 44°05'27"S, 169°24'56"E, 1580 m, 16 Feb 2012, *M.A.M. Renner 6148*, NSW895456.

### Radula buccinifera

(Hook.f. et Taylor) Taylor ex Gottsche, Lindenb. et Nees Synopsis Hepaticarum 2: 261. 1845.

http://species-id.net/wiki/Radula_buccinifera

Figs 11 –13

Jungermannia buccinifera Hook.f. et Taylor. London Journal of Botany 3: 580. 1844.

#### Type:

Australia: Tasmania: “ Van Diemen’s Land ”. Syntypes: Voyage of HMS Erebus & Terror. *J.D. Hooker* *s.n.* 1840, BM, K, FH00284039! L, NY00831294! ex herb Möller S-B25060! ex herb Lehmann S-B25061! S-B25062! YU; “ Van Diemen’s Land ”, *Gunn*, FH00284037! “ Van Diemen’s Land”, on *Sticta glabra*, without collector, date or number, FH00284038!

*Radula wattsiana* Steph. *Species Hepaticarum* 4: 211. 1910.

Type: Australia: Cambewarra Mountain, 1903, leg. *Rev. W.W. Watts*, ex herb. Levier No. 4144 in herb. Steph (as *Radula plicata*). Lectotype (designated by Castle (1963 p. 46): G00264951! isolectotypes: FH! NSW764184!

#### Description.

[from NY00831294 and MEL38047] Forming interwoven mats of shoots, brown in herbarium, shoot systems regularly pinnately branched, with additional pseudodichotomous branching due to production of pairs of subfloral innovations below gynoecia; dimorphic, primary shoots 1.2–1.7 mm wide and up to 40 mm long, secondary shoots smaller in stature and either apparently terminating growth after five to seven leaf pairs, or continuing vegetative growth and attaining similar stature to primary shoots by fourth to sixth pair of leaves; older shoot sectors retaining leaf-lobes.

Stems 130–155 µm diameter, with cortical cells in a single tier of 25–31 rows, medulla cells in 20–35 rows, cortical cell walls yellow-brown pigmented, ventral cortical walls occasionally yellow pigmented, external free cortical cell wall continuously thickened, radial longitudinal cortical walls thin or slightly thickened, inner tangential walls continuously thickened; medulla cell walls faintly yellow-pigmented, with small triangular trigones, walls between trigones lacking thickenings; cortical cells on dorsal stem surface arranged in straight longitudinal rows on young and mature shoot sectors. Leaf insertion variable within single individuals, reaching the dorsal stem mid-line or not, leaving zero to three dorsal cortical cell rows leaf-free, dorsal leaf-free strip usually present; leaf insertion not attaining the ventral stem mid-line, leaving two to five ventral cortical cell rows leaf-free. Leaf lobes rotund-ovate, 600–845 µm long by 400–655 μm wide, contiguous, not to weakly falcate, acroscopic base not sharply deflexed away from stem, plane, not interlocking over the dorsal stem surface, stem visible between leaf lobes in dorsal view; margins irregularly but minutely repand, otherwise entire, the interior lobe margin shallowly ampliate, not or only just reaching the opposite stem margin, antical margin curved, exterior margin sharply curved through nearly 100°, postical margin shallowly curved or straight; angle between postical lobe margin and keel c. 135°. Lobules quadrate to rhombic when small and large, one eighth to one sixth the lobe area, 310–475 µm long by 215–420 μm wide; keel curved in small stature lobules, to straight, to arched in large stature lobules, angle between keel and stem 135°, keel turning through 90° mostly at keel-lobe junction, keel apex and postical lobe margin with shallow notch; interior lobule margin free for one third its length, free portion weakly ampliate small stature lobules to moderately ampliate on large stature lobules, extending at most half way across the ventral stem surface; acroscopic margin S-shaped (typical in situ) to straight (when flattened), apical portion inclined toward stem; apex obtuse to acute; free exterior margin straight to curved, occasionally with a small knee above the lobe-lobule junction; margins plane, entire or shallowly repand; lobe-lobule junction slightly antical to, or level with, the acroscopic end of stem insertion; attached to stem along 0.66 of the interior margin, stem insertion more or less linear, gently curved at acroscopic and basiscopic ends, not revolute; lobule apex bearing a single papilla, with another two papilla situated on the interior lobule margin above the stem insertion. Leaf lobe cells rounded-oblong, not arranged in rows, unequally sized, 10–23 µm long by 11–19 μm wide; thin walled with small triangular trigones, medial wall thickenings absent; cells of lobe margin smaller than those of middle, quadrate to rectangular, 9–15 µm long and wide, interior and exterior cell walls not differential thickened, cell lumen not bulging medially, leaf lobe cell surface unornamented, smooth. Oil-bodies not known. Asexual reproduction absent. Dioicous. Androecia on lateral branches that usually terminate following production of 2–4 pairs of antheridial bracts, occasionally these branches continue vegetative growth; bract lobules epistatic, keel deeply curved, bucket-like, free apical portion triangular, apex obtuse, inner margin ampliate, plane; lobes rounded, not caducous; antheridia 1–2 per bract. Gynoecia terminal on leading shoots, subtended by two subfloral innovations, usually full-sized and again fertile; archegonia 130–155 µm tall, archegonia neck six cell columns, 6–8 per gynoecium, on a small disc of tissue, interspersed with paraphyses of 1–3 moniliform cells capped by a hyaline papilla, not encompassed by a protoperianth. Female bracts in one pair, symmetrical, tightly imbricate, elliptic-obovate, weakly falcate, lobe 725–845 μm long by 390-610 μm wide, margins entire; lobules rectangular, one half the lobe area, apex obtuse to broadly acute, keel arched, margins entire; bract insertion lines interlocking dorsally and ventrally, insertion equitant. Perianths 2670–3650 µm long and 630–930 µm wide at mouth, mouth repand, more or less parallel sided for upper third, then tapering to tubular stem perigynium comprising the lower third to half, faint bulb in basal third, broadest c. one third from mouth where 660–950 µm wide, walls bi- or tri-stratose at junction with perigynium, unistratose above, cell walls with triangular trigones. Long stem perigynium present, 5-6 stratose throughout, cell walls not thickened or brown-pigmented, hyaline, perianth-calyptra junction elevated above female bracts on 9–15 tiers of cells. Calyptral perigynium present, 2–4 stratose at base, unistratose above, unfertilised archegonia elevated on surface of calyptra.

**Figure 11.**
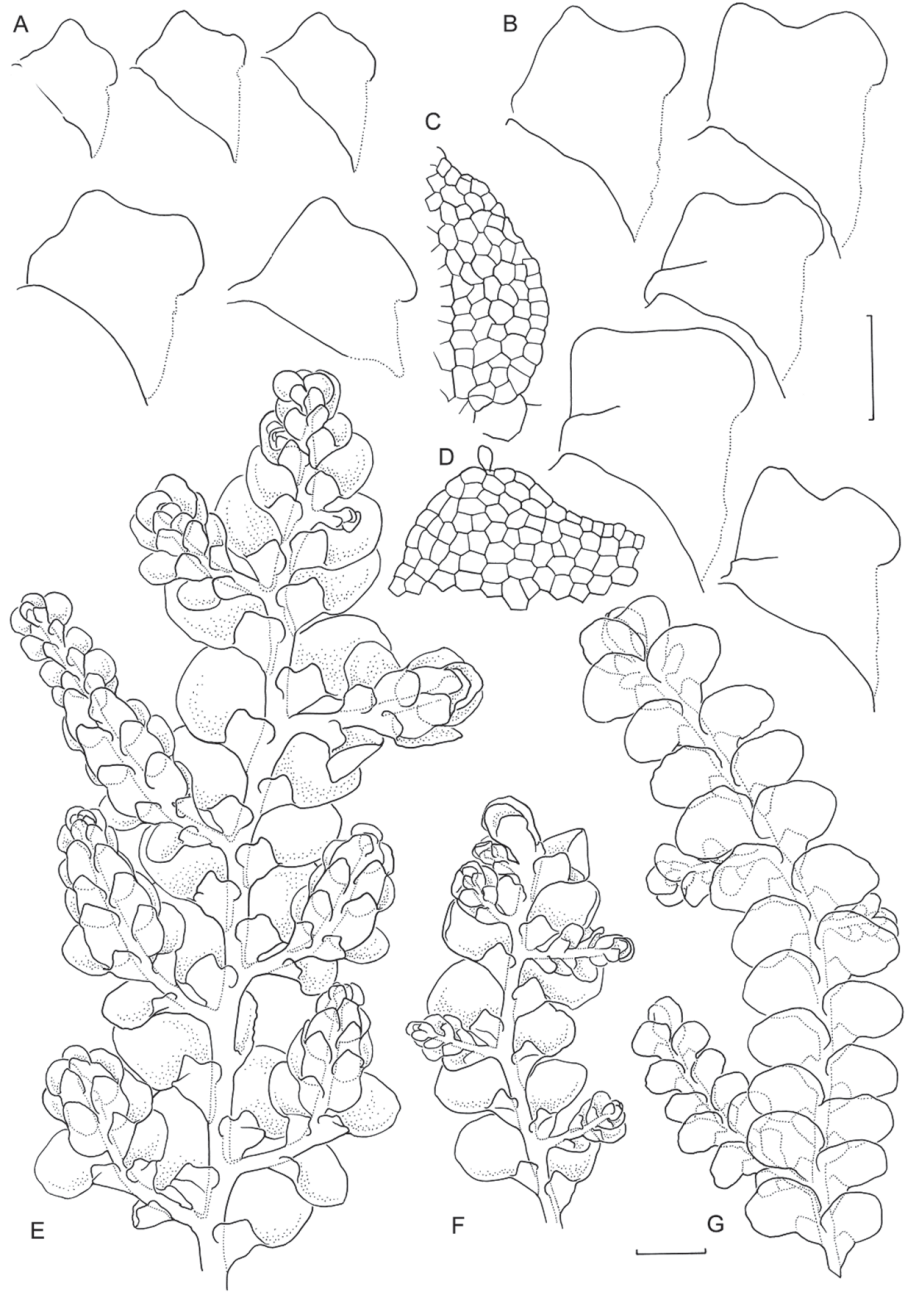
*Radula buccinifera* line drawings 1. **A** Five lobules from secondary shoots showing variation in size and shape **B** Five lobules from primary shoots showing variation in size and shape **C** Cellular detail of free interior lobule margin **D** Cellular detail of lobule apex **E** Ventral view of male shoot **F** Ventral view of female shoot **G** Dorsal view of shoot. Scale bars: **A, B**: 240 µm. **C–D**: 60 µm, **E–G**: 600 µm. **A–E** from MEL2054447, **F–G** from NY00831294.

**Figure 12.**
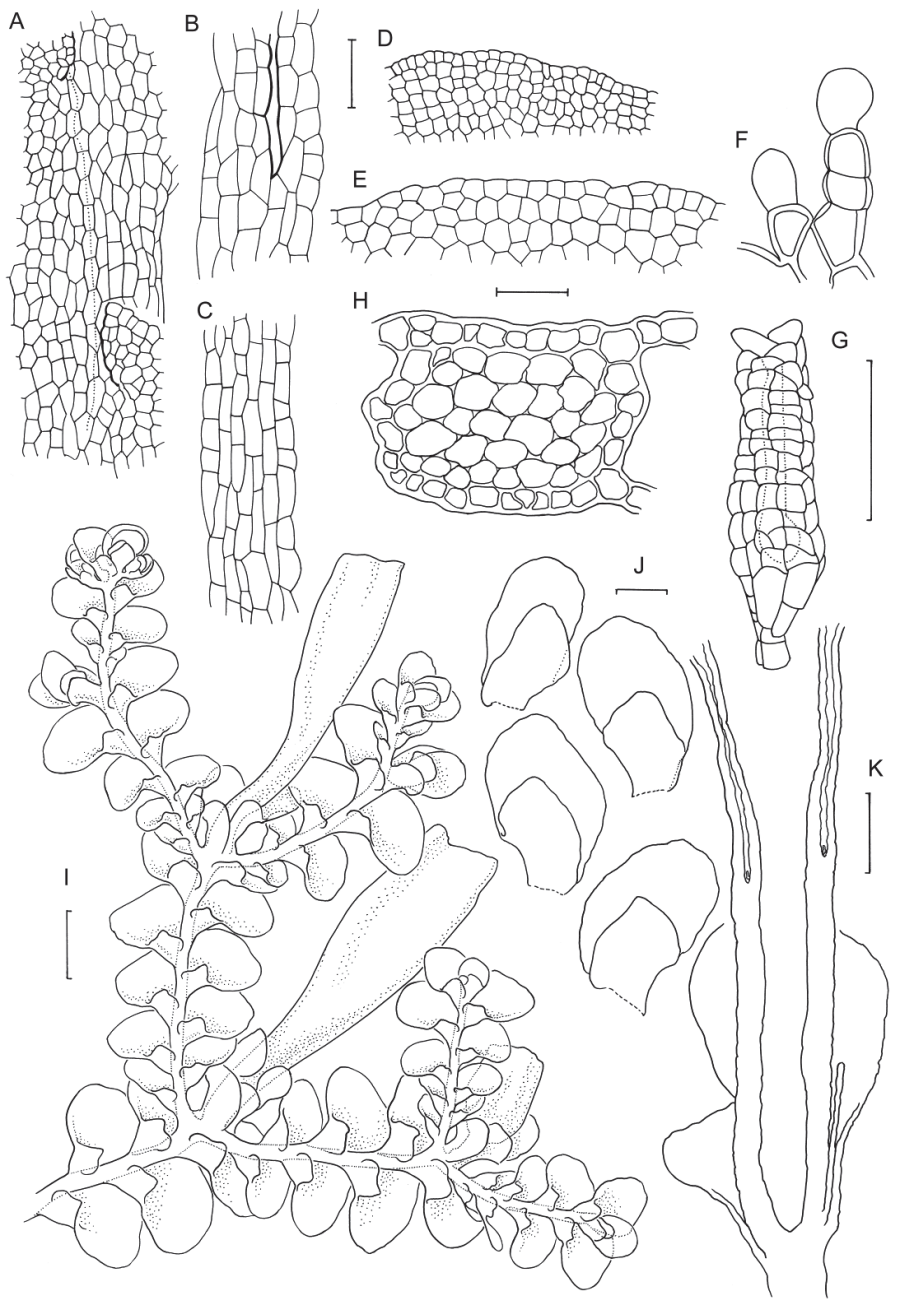
*Radula buccinifera* line drawings 2. **A** Dorsal stem surface showing leaf insertion lines not attaining dorsal stem mid-line, leaving a dorsal leaf-free strip **B** Cellular detail of junction between stem perigynium, perianth wall, (at right) and calyptral perigynium (at left) **C** Cellular detail of stem perigynium wall **D** Cellular detail of perianth mouth **E** Cellular detail of lobe marginal cells **F** Paraphyses among archegonia, capped by slime-papillae **G** Archegonium **H** Transverse section of stem from primary shoot **I** Ventral view of perianth bearing shoot section **J** Four female bracts **K** Longitudinal section of perianth. Scale bars: **A–C, D, G**: 60 µm, **E, F**: 40 µm, **J, K**: 240 µm, **I**: 600 µm. **A, E** from NY00831294, **B–D, F–K** from MEL38047.

**Figure 13.**
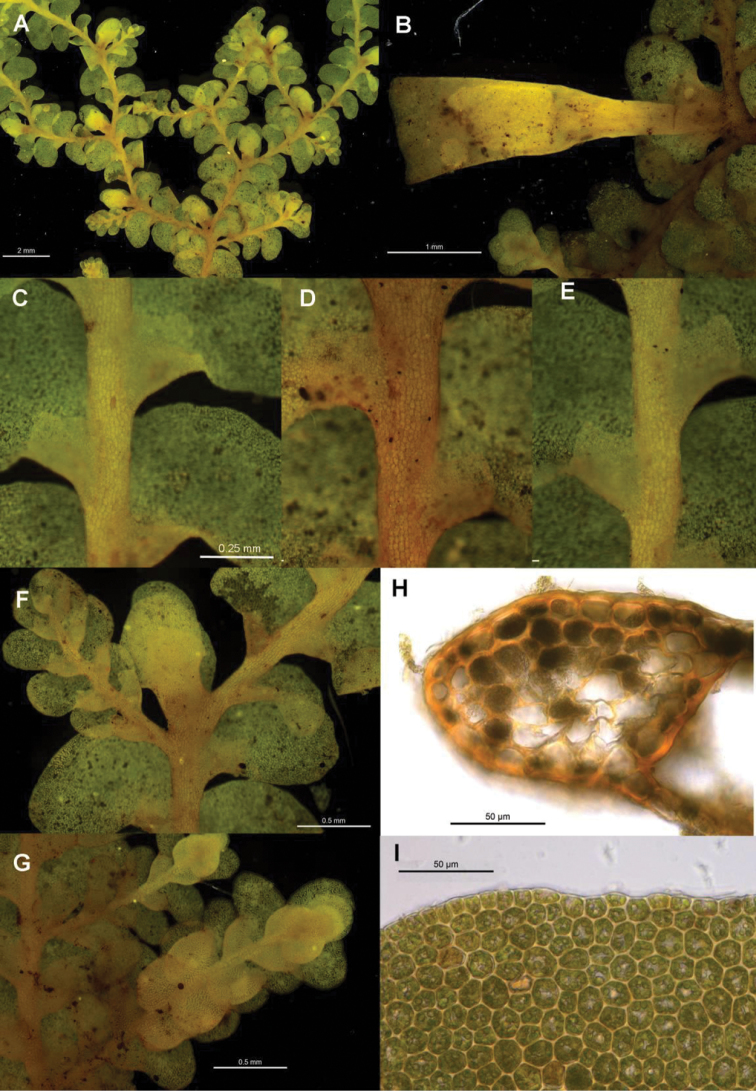
*Radula buccinifera* pictures. **A** Ventral view of shoot **B** Mature perianth. C–E: Ventral view of shoots and lobules **F** Gynoecium **G** Androecium **H** Transverse sections of stems from primary shoot **I** Leaf-lobe marginal cells **G** NSW875961, other NSW909436.

#### Etymology.

Horn-bearing.

#### Distribution and ecology.

*Radula buccinifera* is endemic to Australia, where it is widespread throughout Tasmania, and Victoria but more restricted in New South Wales, being confined to the eastern side of the Great Dividing Range and associated escarpments. In Western Australia it is confined to the far south-west. The northern limit of this species has not been identified, currently the northernmost locality is Point Lookout in New England National Park, in association with *Nothofagus moorei*. Cool temperate rainforests dominated by *Nothofagus* also occur in south-east Queensland and *Radula buccinifera* may be be found in these areas.

Casual field observation suggests the elevational range occupied by *Radula buccinifera* is correlated with latitude. In Tasmania and Victoria *Radula buccinifera* occurs across a broad elevational range from sea level to 1500 m,encompassing a range of habitat types from lowland to montane forests, including wet sclerophyll forest and cool temperate rainforest, wetlands, and in alpine scrub and within habitats may occupy a range of microsites from twigs, branches, tree trunks and tree bases, exposed tree roots on the forest floor, to rotting logs, exposed soil on forest banks, dripping rocks adjacent waterfalls, and on rocks within stream beds, sometimes under running water.

The ecological range decreases with latitude; the further north, the more restricted to foothills, escarpments, and mountains in association with cool temperate rainforests. This altitudinal contraction is associated with restriction in the diversity of microhabitats occupied, such that at the northern end of its range, where *Radula buccinifera* occurs at 1000 m or higher, and is always encountered as a lithophyte most frequently on vertical rock faces associated with bluffs and outcrops.

*Radula buccinifera* is one of three [*Radula novae-hollandiae*, *Radula strangulata*, *Radula buccinifera*] *Radula* species in south-eastern Australia that may be found growing under running water. On rocks it frequently co-occurs with *Radula novae-hollandiae*, forming mixed mats. The type specimen of *Radula novae-hollandiae* comprises such a mixed patch. In north-eastern New South Wales it may co-occur and form mixtures with a number of other *Radula* species, initially presenting as confusing and exceptionally variable individuals.

#### Variation.

Individual populations exhibit generous amplitude in shoot stature and lobule shape. Lobules vary from rhombic-quadrate with little ampliation of the interior margin and a straight antical margin, to quadrate with a pronounced ampliate interior margin and an S-shaped antical margin having a distinct, obtuse, lobule apex. Colour of individuals varies, from glaucous yellow-green to dark green, in part this appears correlated to microhabitat, with epiphytic plants tending yellow green, and lithophytic plants being dark green.

The amplitude of morphological variation expressed between individuals appears negatively correlated with latitude. The greatest morphological variation between individuals in plant colour, shoot size and lobule shape occurs in the southern end of the distribution. Individuals vary from brown-green, mid-green and glaucous green and from small to large, with associated differences in lobule shape. In Central and Northern Coast, and Northern Tableland regions in the northern part of *Radula buccinifera* range, individuals are fairly consistent in morphology being large, mid-green, and having lobules whose antical margin is more or less straight, with an pronounced ampliate free interior margin.

**Recognition.** Despite the variability exhibited by *Radula buccinifera* this species is relatively easy to recognize. The first clue to identity comes from the habitat and microhabitat the plants occupy; *Radula buccinifera* inhabits forest interiors, frequently in microsites on or close to the forest floor that are often well shaded. Other species of the *Radula buccinifera* complex with which *Radula buccinifera* could be confused on morphological grounds almost all occupy quite different habitats and microsites.

*Radula australiana* inhabits subalpine and alpine habitats typically dominated by shrubland, tussockland or grassland, and is almost always found above tree-line. However, *Radula australiana* and *Radula buccinifera* may co-occur in forest interiors on forested mountain tops in Victoria and Tasmania, particularly in association with exposed large granite boulders, for example at Mt. Ellery in Errinundra National Park, Victoria. The most accessible morphological character by which *Radula buccinifera* differs from *Radula australiana* is the shape of the leaf lobes, where the junction between the lobe and keel forms a simple angle in *Radula buccinifera* and the leaves are not or weakly falcate as a result. In *Radula australiana* the keel runs more or less seamlessly into the lobe outline, the two following the same curve. If this character proves ambiguous, lobule shape is a good source of discriminating characters. Lobule shape should always be assessed on the basis of hydrated, slide-mounted material, and at least 50× magnification. When hydrated the lobules of *Radula buccinifera* are quadrate to rhombic, and one eighth to one sixth the lobe area. The antical margin is usually (northern plants excepted) S-shaped in situ (straight when flattened), but this is more pronounced with a deep medial curve, and the apex is obtuse. The lobules of *Radula australiana* are quadrate to rectangular, and one quarter the lobe area. The antical margin is S-shaped in situ and when flattened, but the medial curve is relatively shallow, and the apex varies from obtuse to acute. Further differences exist in the lobe marginal cells that bulge in *Radula buccinifera*, but are crenulate, due to medial thickening on the external cell wall in *Radula australiana*. Finally, if all of these characters prove ambiguous, diagnostic differences can be derived by counting the number of rows of dorsal cortical cells that are not crossed by the leaf insertion lines. In *Radula buccinifera* 0–3 cortical stem cell rows are leaf free, whereasno cell rows are leaf-free in *Radula australiana*. Obviously multiple counts from a few shoots are required. If none of these characters leads to a satisfactory conclusion, consider the possibility that the material at hand comprises a mixed collection.

*Radula buccinifera* may be confused with *Radula demissa*, with which it co-occurs in Victoria and Tasmania where the two species may occupy similar microhabitats. For characters distinguishing these two species, see the recognition section of *Radula demissa*.

*Radula buccinifera* could also be confused with *Radula strangulata*, as the two species occupy similar microhabitats, both have a dorsal leaf-free strip, and are identical in overall appearance. Lobule shape differences are possibly the best, and only, source of discriminating morphological characters. In *Radula buccinifera* the antical lobule margin is typically straight and perpendicular to the stem and the lobule apex is obtuse at least in large lobules. In contrast the antical margin of *Radula strangulata* is either inclined toward the stem or arched and the lobule apex is broadly acute in large lobules. Aquatic morphs of *Radula strangulata* have longitudinally rectangular lobules, whose straight exterior margin runs parallel with the stem and whose interior margin is ampliate over the ventral stem surface. In *Radula buccinifera* the lobule is trapeziform or quadrate, and not ampliate to the same degree. More substantial characters have not been identified. Some interpretations of morphology would find (and have found) strong evidence for the synonymisation of *Radula buccinifera* with *Radula strangulata* from this circumstance. However, here as in other liverwort complexes morphological unity and the existence of morphological continua are immaterial to the phylogenetic structure underpinning the observed morphological diversity, and the existence of populations of both species intermediate in size and shape does not indicate phylogenetic unity (Renner et al. 2013).

The name *Radula buccinifera* has been applied to specimens belonging to a wide variety of unrelated species from tropical Queensland, even as far north as Moa Island in the Torres Strait (i.e. CANB9500180). However, with the exception of two specimens collected by Pentzke in 1882, one of which is a mixture of elements from at least two locations (see discussion of the type of *Radula mittenii*), all records of known provenance from tropical Queensland are based on misidentifications. *Radula buccinifera* can be distinguished from virtually every species in Queensland by 1) the presence of a stem perigynium in the perianth, 2) the production of two subfloral innovations, 3) the absence of secondary cell wall thickening in the stem medulla, 4) the sub-dimorphic shoot systems, 5) the rhombic lobules, 6) the presence of two papillae on the interior lobule margin, 7) the smooth leaf-lobe cell surfaces. *Radula buccinifera* may be confused with *Radula notabilis*, and *Radula imposita*; for distinguishing characters see the recognition section of those species.

#### Remarks.

*Radula buccinifera* is among the most widely misunderstood species of *Radula*, having been confused in herbarium collections with almost every other *Radula* species in Australia, and several that aren’t. Misidentifications of *Radula buccinifera* have been the basis for several records of species now excluded from the Australian flora, including *Radula physoloba* and *Radula plicata*. Confusion with *Radula physoloba* seems attributable to R.A. Bastow, all of whose specimens identified as *Radula physoloba* in MEL ex herb. R.A. Bastow are *Radula buccinifera*, and confusion with *Radula plicata* is attributable to Stephani, who misidentified several gatherings by Weymouth and Bastow.

*Radula buccinifera* was understandably confused with *Radula strangulata* by Mitten (1855) who cited collections by Colenso, Stephenson, and Lyall from the southern North Island in his treatment for Hooker’s Flora of New Zealand, and this confusion has been perpetuated in various manifestations. Allison and Child (1975) illustrated *Radula strangulata* as *Radula buccinifera*, and again in the next figure as *Radula levieri* Steph. Renner (2005) made a different, and less forgivable, error when he presented a key to New Zealand species that included *Radula buccinifera*. The plants he referred to were actually *Radula demissa* M.A.M.Renner, and his error was based on mis-interpretation of type material.

Castle, however, appears to have been hopelessly confused regarding the identity of *Radula buccinifera*, arbitrarily placing specimens of *Radula buccinifera*, *Radula demissa*, and *Radula strangulata* under the names *Radula buccinifera*, *Radula mittenii*, and *Radula wattsiana*. As an example, the figure illustrating *Radula buccinifera* in Castle (1967) includes a male shoot of *Radula strangulata*. Castle maintained that *Radula mittenii* differed from *Radula buccinifera* in its more falcate leaf lobes, and the shorter perianths and he may have had *Radula demissa* in mind in this assessment.

Yamada (1984) synonymised *Radula mittenii* with *Radula buccinifera* on the basis of his examination of ‘holotype’ material held in g, and his assertion that the differences observed were environmental. Yamada’s identification of holotype material is incorrect, his interpretation was derived from examination of duplicate material in Geneva and the implications of this are discussed below under *Radula mittenii*. The synonymisation of *Radula mittenii*, having a type from tropical Queensland, with *Radula buccinifera* by Yamada (1984) seems to have encouraged the application of the name *Radula buccinifera* to a range of specimens from tropical Queensland, to the point where nearly every species occurring in Queensland has, at some point, been identified as *Radula buccinifera*. Beyond the specimen collected by Pentzke, no populations attributable to *Radula buccinifera* from tropical Queenslandexists within herbaria, and the species has not been observed during the course of recent fieldwork in the Wet Tropics of Queensland.

#### Nomenclature.

So (2005) identified a Hooker collection from Van Diemens Land as the holotype of *Radula buccinifera*. This is inappropriate as Hooker and Taylor (1844) did not identify a single type collection, the only specimen details given in the protologue were “Van Diemen’s Land”. Collections from “Van Diemen’s Land” by both R. C. Gunn and J. D. Hooker were made prior to 1844 and were studied by Hooker and Taylor for their contribution to Hepaticae Antarcticae, as made clear in the subtitle to Hooker and Taylor’s (1844) publication, and by the presence of duplicates from both collectors in Taylors own herbarium. At least three gatherings may comprise the syntype series, being the more or less pure gatherings made by Gunn and Hooker, and a gathering on *Sticta*, whose collector has not been recorded. Furthermore, Castle (1967) had attempted to lectotypify *Radula buccinifera*, though we are unclear about exactly what Castle intended in his lectotypification. Castle’s (1967) lectotypification of *Radula buccinifera* is somewhat obscured by his own commentary. In the list of specimens examined he states ‘Van Dieman’s Land, *the Type* (BM K and Y) *and* Van Dieman’s Land, R.C. Gunn (BM and K)’ [italics ours]. Here Castle appears to identify two groups of specimens, one of which was collected by Gunn and was not identified as the lectotype. But Castle then notes ‘we may assume that those collections which bear the label Van Diemen’s Land are portions of the collection upon which Thomas Taylor based his *Jungermannia buccinifera*. Several samples of this type, preserved in the herbaria of the British Museum and of Kew Gardens, also include in the label the name Gunn. Gunn presumably made the type collection as his name, as collector, appears in the title of the article in which the original description of *Jungermannia buccinifera* was published, without data’. Here Castle implies that the Gunn collection should be regarded as the lectotype. All gatherings comprising the syntype series we have seen, being duplicates of both Hooker and Gunn’s collections, and the specimen *sine. coll.* are unambiguously assignable to *Radula buccinifera*, so application of the name *Radula buccinifera* will remain the same regardless of which gathering and which particular specimen the species is lectotypified on. Castle’s lectotypification at best requires narrowing because he did not identify a single specimen as type, and at worst lectotypification requires repeating because it is not quite clear whether Castle identified a single gathering.

#### Specimens examined.

Australia: New South Wales: Northern Tablelands, The Cascades, Point Lookout, New England National Park, 30°30'S, 152°24'E, 1010 m, 11 March 1990, *A.K. Brooks 204 & E.A. Brown*, NSW233743; Northern Tablelands, Washpool National Park, Coombadjha Stream catchment, Washpool walk. Between Bellbird Campground and Coombadjha Stream, 29°28'01"S, 152°19'19"E, 800 m, 11 Apr 2011, *M.A.M. Renner 5246*, NSW875783; Northern Tablelands, Mount Hyland Nature Reserve, Mount Hyland Circuit Track, southern summit, 30°10'30"S, 152°25'38"E, 1380 m, 13 Apr 2011, *M.A.M. Renner 5257*, NSW875805; Northern Tablelands, New England National Park, Point Lookout area, Lyrebird Track between Banksia Point and Weeping Rock, 30°29'24"S, 152°24'32"E, 1470 m, 16 Apr 2011, *M.A.M. Renner 5288*, NSW875835; North Coast, Myall River State Forest, Strike-a-light camping area, 32°17'S, 152°05'E, 210 m, 5 Apr 2002, *E.A. Brown 2002/18 & B.J. Conn*, NSW491702; Central Tablelands, Blue Mountains National Park, Waterfall Reserve, Waterfall track, 33°31'S, 150°22'E, 860 m, 24 September 2001, *N. Klazenga & V. Stajsic 2809*, MEL2137146; South Coast, Rutherford Creek near Piper'S, Lookout, c. 12 km WNW of Bemboka, 36°36'S, 149°27'E, 700 m, 17 August 1985, *K.R. Thiele 1001*, MEL2273913. Central Tablelands, Mount Victoria, track at Thomas Mitchell Monument Hill, 33°34'S, 150°15'E, 13 Mar 1989, *E.A. Brown 89/35*, NSW436068; Central Coast, Nowra, Cambewarra Mountain, rocks near Cambewarra Lookout, 34°47'56"S, 150°34'36"E, 470 m, 6 Jun 2011, *M.A.M. Renner 5303 & E.A. Brown*, NSW877190; Northern Tablelands, Barrington Tops National Park, Dilgry River, Devils Hole Campground., 31°54'55"S, 151°28'59"E, 1400 m, 16 Dec 2011, *M.A.M. Renner 5868*, NSW898654;

Victoria: Wilsons Promontory, headwaters of Blackfish Creek, Wilsons Promentory National Park, 39°02'S, 146°23'E, 27 July 1996, *D.A. Meagher* *s.n.*, MEL240137; Gippsland Highlands, Tarra Bulga National Park, Bulga Section, Suspension Bridge circuit, 38°26'S, 146°34'E, 650 m, 21 Feb 1997, *A.W. Thies FN1626K*, MEL241693; Eastern Highlands, Toolangi/Black Range State Forest, Sylvia Creek Road, Wirrawilla rainforest walk, 37°31'S, 145°31'E, 650 m, *N. Klazenga & V. Stajsic 2325*, MEL2111876; Otway Range, upper Calder River, 8 miles west of Apollo Bay, 38°45'S, 143°31'E, 19 Nov 1995, *J.H. Willis* *s.n.*, MEL1514803; Otway Range, Grey River Road, Angahook-Lorne State Forest, 28 km NE of Apollo Bay, 38°38'S, 143°46'E, 480 m, 5 Dec 1996, *H. Streimann 58978*, MEL2300394; Gippsland Plain, Tara Bulga National Park, near Yarram, Gippsland, 38°34'S, 146°40'E, Aug 1960, *K. Healey* *s.n.*, MEL38047; Grey River Reserve, Angahook – Lorne State Park, 17 km ENE of Apollo Bay, 38°39'S, 143°49'E, 280 m, 5 Dec 1996, *H. Streimann 58874*, CANB9802545; East Gippsland, Errinundra National Park, Errinundra Road, between Ada River and Errinundra Saddle, 37°20'47"S, 148°51'43"E, 921 m, 04 Mar 2011, *M.A.M. Renner 5176 & E.A. Brown*, NSW875959; East Gippsland, Errinundra National Park, Errinundra Road, between Ada River and Errinundra Saddle, 37°20'47"S, 148°51'43"E, 921 m, 04 Mar 2011, *M.A.M. Renner 5177 & E.A. Brown*, NSW875960;

Tasmania: Furneaux Group, Flinders Island, 660 m east of summit of Big Badger Hill, 40°02'S, 148°01'E, 137 m, 29 Jul 2004, *J.S. Whinray B1861*, MEL2209244; North West, Dismal Swamp Nature Reserve, 41°59'S, 144°51'E, 40 m, 23 Mar 2000, *A. Moscal 30979*, HO558827; Southwest National Park, Styx Valley, Big Tree Reserve, river walk (7 km SSE of Maydena), 42.81354°S, 146.65559°E, 350 m, 6 Dec 2007, *B. Shaw 6463*, DUKE; Marriots Falls Track paralleling Tyenna River (4.5 km NE of Maydena, 42.727°S, 146.663°E, 230 m, 6 Dec 2007, *B. Shaw 6511*, DUKE; Mt Field National Park, Growling Swallet (E of F8 East Road, NNE of Florentine Road), ca. 70 km WNW of Hobart, 42.687°S, 146.496°E, 580 m, 4 Dec 2007, *B. Shaw 6287*, DUKE; *B. Shaw 6351*, DUKE; Junee Cave State Park, 3 km NW of Maydena, 42.737°S, 146.596°E, 300 m, 7 Dec 2000, *B. Shaw 6619*, DUKE; Junee Cave State Park, 3 km NW of Maydena, 42.738°S, 146.597°E, 300 m, 4 Dec 2000, *B. Shaw 6361*, DUKE; Southwest National Park, Styx Valley, Big Tree Reserve, river walk (7 km SSE of Maydena), 42.812°S, 146.657°E, 350 m, 6 Dec 2007, *B. Shaw 6476*, DUKE; Scottsdale to St. Helens Highway, 147°40'E, 41°10'S, 800 m, 11 Jan 1974, *D. Norris 31903*, F; Fingal Municipality, St. Mary's Pass, 148°12'E, 41°34'S, 12 Jan 1974, *D. Norris 32308*, F; Deloraine Municipality, Lyons Creek 8 miles W of Liena, c. 700 m, 41°33'S, 146°12'E, 15 Jan 1974, *D. Norris 32735*, F; *D. Norris 32744*, F; *D. Norris 32754*, F; Scotts Road, 41°42'S, 146°34'E, 740 m, 10 Nov 1991, *J. Jarman* *s.n.*, HO310154; Tasmania merid. Mt Wellington, New Town Falls, 17 August 1889, *W.A. Weymouth*, as *Radula plicata* det. Stephani, FH00284645; Tasmania, *Watts*, syntype of *Radula wattsiana* Steph. ex herb. Steph. FH00284647; South West, Waterfall Creek State Reserve, South Bruny Range, 42°24'S, 147°19'E, 100 m, 28 Apr 1993, *A. Moscal 25083*, HO558839; Mt. Field National Park, Lake Dobson Road, near Horseshoe Falls, 42°41'S, 146°42'E, 400 m, 7 Dec 1988, *J.A. Curnow 2602*, HO304660; South West, Deadmans Bay, south coast, 43°32'S, 146°30'E, 5 m, 17 Jan 1987, *A. Moscal 14077*, HO558830; South West, South West Conservation Area, Huon River, adjacent Huon campground, 43°02'17"S, 146°18'12"E, 285 m, 23 Jan 2012, *M.A.M. Renner 5939 & E.A. Brown*, NSW895271; Central Highlands, Jerusalem Walls National Park, Southern side of Mt Jerusalem, 41°49'23"S, 146°18'02"E, 1315 m, 29 Jan 2012, *M.A.M. Renner 6025 & E.A. Brown*, NSW909425; Mersey River, below Lake Rowallan, 41°41'56"S, 146°13'08"E, 440 m, 30 Jan 2011, *M.A.M. Renner 6027 & E.A. Brown*, 30 Jan 2012, NSW909430; Mersey River, below Lake Rowallan, 41°41'56"S, 146°13'08"E, 440 m, 30 Jan 2012, *M.A.M. Renner 6032 & E.A. Brown*, NSW909436;

Western Australia: Mt Chudalup, 17 km SSE of Northcliffe, 34°46'S, 116°05'E, 185 m, 14 Sep 1994, *H. Streimann 54341*, CANB9504479, PERTH04957172; Denmark Shire, mid-slopes on the east side of Mt Hallowel on the Bibbulmun track, 35°0'3"S, 117°19'3"E, 14 Aug 2000, *B.G. Hammersley 2598*, PERTH05803047; Cascades S of Pemberton, 16 Dec 1973, *N.G. Marchant 73/45*, PERTH01929305; NE of Mount Frankland, 34°49'17"S, 116°47'36"E, 26 Aug 1997, *K.A. Redwood 612*, PERTH04983963; Stirling Range National Park, Stirling Range National Park, Toolbrinup track, boulder slope at base of hill, 34°23'11"S, 118°02'59"E, 765 m, 25 Aug 2009, *E.D. Cooper 09/067 & E.A. Brown*, NSW970847; Stirling Range National Park, Stirling Range National Park, Toolbrinup track, boulder slope at base of hill, 34°23'11"S, 118°02'59"E, 765 m, 25 Aug 2009, *E.D. Cooper 09/068 & E.A. Brown*, NSW970854.

### Radula demissa

M.A.M.Renner sp. nov.

http://species-id.net/wiki/Radula_demissa

Figs 14 –16

#### Type.

New Zealand, North Island, Puaiti Bush south of Rotorua, ca 1,600 ft, 20 Jun 1931, *K.W. Allison*, (holotype: CHR587329, isotypes: AK, F, NSW, WELT).

*Radula epiphylla* Colenso Transactions and Proceedings of the New Zealand Institute 21: 71. 1888 [1889] *nom. illeg.* (Art. 53.1) *non R. epiphylla* Mitt. ex Steph. Hedwigia 23: 151. 1884.

Type. New Zealand: North Island: ‘Epiphytical on fronds of *Hymenophyllum* (sps); damp woods, Dannevirke, County of Waipawa, 1888, *W. C*[*olenso*]’. Not located and non vidi, however *Colenso a. 2254*, on *Hymenophyllum* leaf, ex herb Steph, received 1897, BM! is possibly a duplicate of *Colenso*’s type.

#### Diagnosis.

Similar to *Radula buccinifera* and *Radula strangulata* but differs from both species in the leaf insertion attaining the dorsal stem mid-line, leaving no dorsal cortical cell rows leaf-free, the leaf lobes overlapping across the dorsal stem, such that the stem is not visible from above, the falcate leaf lobes, the rhombic lobules with antical margin sloping inwards toward the stem at an angle of c. 45°, and in its typically epiphytic and epiphyllous habit.

**Description.** [from CHR587329 and AK280339] Forming hanging wefts or patches of interlocking shoots, brown-green when fresh, fading to a glossy tan or brown in herbarium; shoot systems regularly pinnately branched in male plants and sterile female plants, but pseudodichotomous in fertile female plants due to production of pairs of subfloral innovations below gynoecia; dimorphic, 1.0–2.0 mm wide and up to 40 mm long, branches smaller in stature than parent shoot; older shoot sectors retaining leaf-lobes. Stems 115–175 µm diameter, with cortical cells in a single tier of 20–35 rows. Cortical cell walls yellow-brown to brown pigmented; external free cortical cell wall heavily and continuously thickened, radial longitudinal cortical walls thin, inner tangential walls thin or continuously thickened; medulla cells in 22–43 rows; cell walls yellow-brown pigmented, with small to medium triangular trigones, walls between trigones lacking thickenings. Cortical cells on dorsal stem surface arranged in straight longitudinal rows on young and mature shoot sectors. Leaf insertion reaching dorsal stem mid-line, leaving no dorsal cortical cell rows leaf-free, except in instances where stem growth appears to have introduced an additional cortical cell row to the dorsal stem surface, in which case a single row of dorsal cortical cells is leaf-free, but this row is discontinuous between adjacent leaf pairs; leaf insertion not attaining the ventral stem mid-line, leaving two or three ventral cortical cell rows leaf-free. Leaf lobes ovate-falcate, 650–1100 µm long by 430–900 μm wide, larger on leading shoots, contiguous to imbricate, falcate, acroscopic base not sharply deflexed away from stem, flat, obliquely patent, interlocking over the dorsal stem surface, stem not visible between leaf lobes in dorsal view; margins irregularly but minutely repand, entire to weakly minutely crenulate, the interior lobe margin ampliate, covering the dorsal stem surface and reaching or exceeding the opposite stem margin, antical margin curved, exterior margin curved, postical margin curved; angle between postical lobe margin and keel 60–90°. Lobules rhombic when small to widely rhombic, one eighth to one sixth the lobe area, 255–535 µm long by 180–385 μm wide, larger on leading shoots; keel straight to curved, angle between keel and stem 135°, keel apex and postical lobe margin notched; interior lobule margin free for one fifth to one third its length, free portion ampliate, extending at most half way across the ventral stem surface; acroscopic margin S-shaped, apical portion inclined towards stem; apex rounded to apiculate-rounded; free exterior margin curved, margins plane, entire; lobe-lobule junction antical to the acroscopic end of stem insertion in small lobules to level with or below it in larger lobules; attached to stem along 0.66 to 0.8 of the interior margin, stem insertion curved, particularly at basiscopic end, not revolute at acroscopic end; lobule apex bearing a single papilla, with another two papilla situated on the interior lobule margin above the stem insertion. Leaf lobe cells rounded-oblong, not arranged in rows, unequally sized, 14–25 µm long by 9–18μm wide, thin walled with small triangular trigones, medial wall thickenings absent; cells of lobe margin smaller than those of leaf middle, quadrate to rectangular, 9–15 µm long and wide, interior and exterior cell walls not differential thickened, cell lumen not or only slightly bulging medially; leaf lobe cell surface unornamented, smooth or weakly bulging; upper lobe wall differentially thickened over cell lumen, forming a weak papilla. Oil-bodies two or three per cell, ellipsoidal, filling cell lumen, light-brown, surface granular, internally homogeneous or with a hyaline droplet. Asexual reproduction absent. Dioicous. Androecia on indeterminate branches that continue vegetative or reproductive growth, androecial bracts in 4–20 pairs, lobules epistatic, keel deeply curved, bucket-like, free apical portion triangular, apex obtuse, moderately deflexed, lobes rounded, not caducous, antheridia not seen. Gynoecia terminal on leading shoots, subtended by two subfloral innovations that are full-sized and again fertile; archegonia 145–190 µm tall, archegonia neck six cell columns, 6–8 per gynoecium on a small disc of tissue, encompassed by the protoperianth; female bracts in one pair, symmetrical, tightly imbricate, ovate-falcate, lobe 870–940 μm long by 450–560 μm wide, margins entire to repand; lobules ovate to trullate, one third to one half the lobe area, apex rounded to obtuse, keel arched, margins entire; bract insertion lines interlocking dorsally but not ventrally, insertion equitant. Perianths 2300–3900 µm long and 710–760 µm at mouth, mouth entire to irregularly lobed, perianth narrowing gently from slightly flared mouth to approximately one third to one half length above base, where 670–790 µm wide, then tapering to narrow cylindrical base, often inflated independent of sporophyte growth, in which case perianth labia roll inward to overlap one another, rather than laying in plane, the inrolling may cause perianths to appear weakly bicornute, perianth walls unistratose above, with bistratose collar 3 or 4 cell tiers high above the perianth-calyptra junction; basal stem perigynium present, 5-6 stratose, cell walls not thickened or pigmented; perianth-calyptra fusion elevated above female bracts on 9–15 tiers of cells; calyptral perigynium present, base of calyptra bistratose, unistratose above, unfertilised archegonia elevated on surface of calyptra.

**Figure 14.**
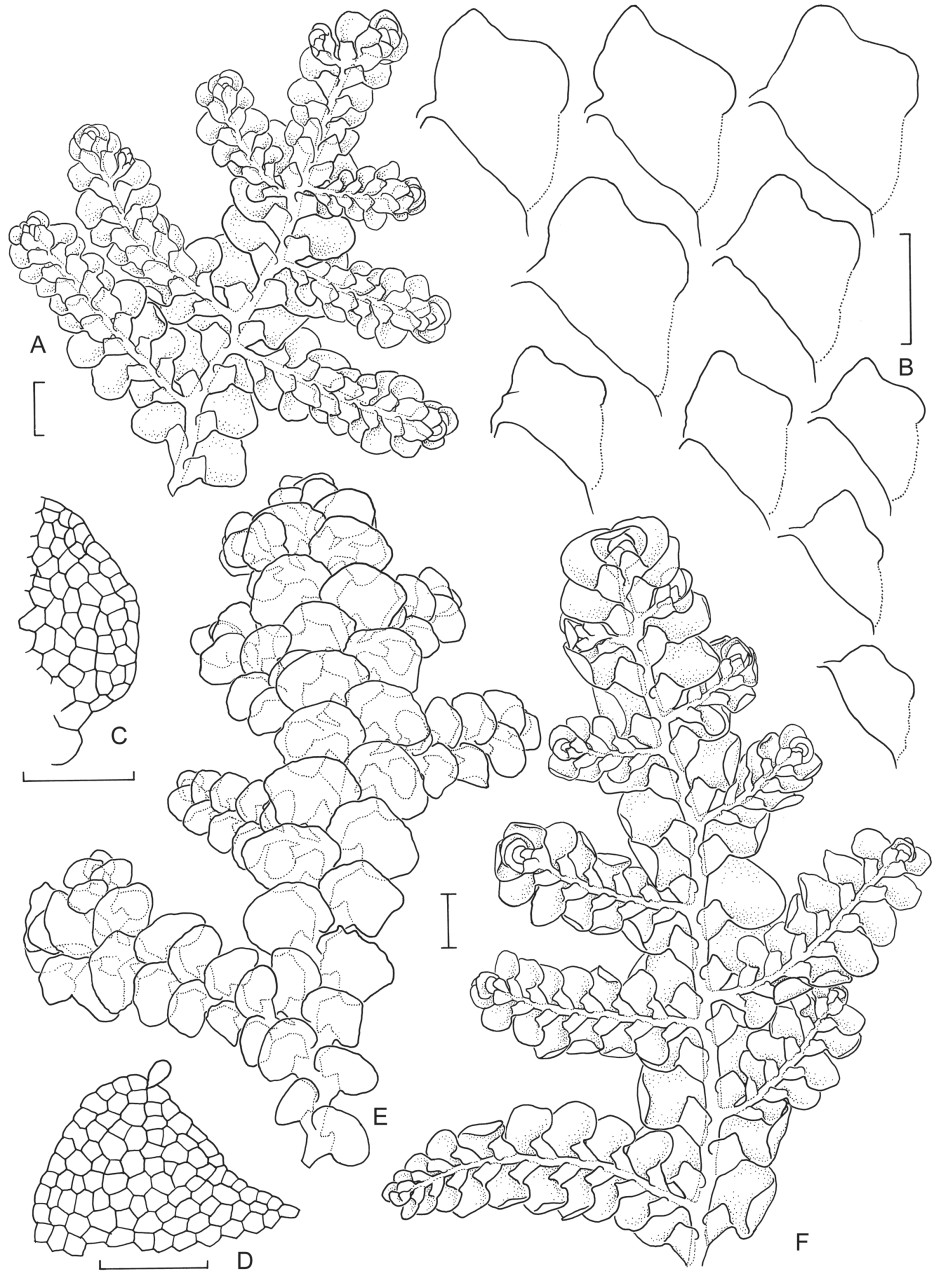
*Radula demissa* Line drawings 1. **A** Ventral view of male shoot **B** Ten lobules, upper five from primary shoots, lower five from secondary shoots, showing variation in size and shape **C** Cellular detail of interior lobule margin **D** Cellular detail of lobule apex **E** Dorsal view of shoot **F** Ventral view of shoot. Scale bars: **A, E, F**: 600 µm, **C, D**: 60 µm, **B**: 240 µm. All from CHR587329.

**Figure 15.**
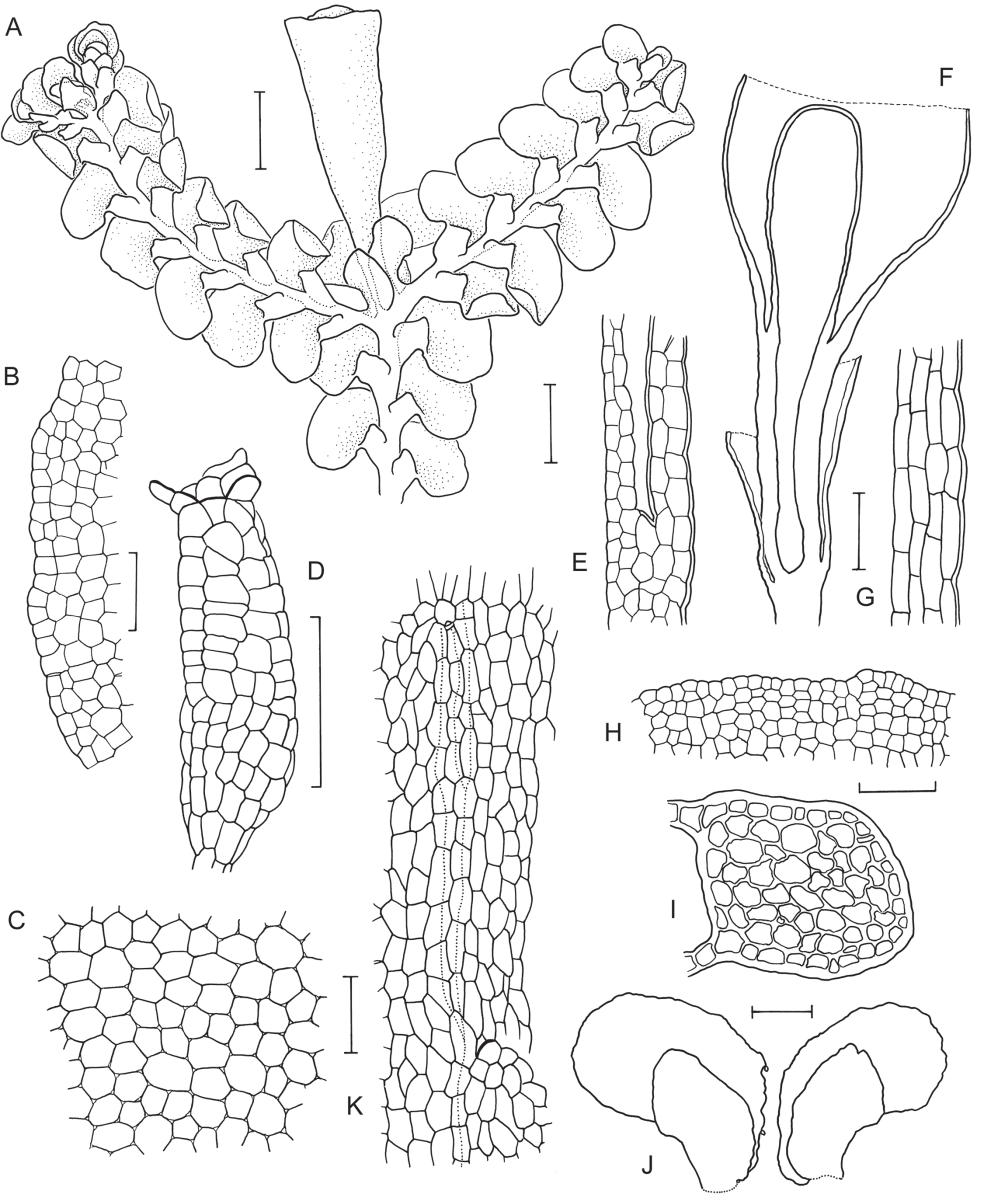
*Radula demissa* Line drawings 2. **A** Perianth bearing shoot sector in ventral view **B** Detail of leaf-lobe marginal cells **C** Detail of leaf-lobe medial cells **D** Archegonium **E** Cellular detail of junction between stem perigynium, perianth wall, (at right) and calyptral perigynium (at left) **F** Longitudinal section of perianth **G** Cellular detail of stem perigynium wall **H** Cellular detail of perianth mouth **I** Transverse section of stem from primary shoot **J** One pair of female bracts **K** Dorsal stem surface showing three possible interpretations of dorsal cortical cell row, two of which has leaf insertion lines meeting at the dorsal stem mid-line, leaving no dorsal leaf-free strip. Scale bars: **A**: 600 µm, **B–C, I**: 40 µm. **D, E, G, H, K**: 60 µm. **F, J**: 240 µm. **E, G**: from NSW895686. Others from CHR587329.

**Figure 16.**
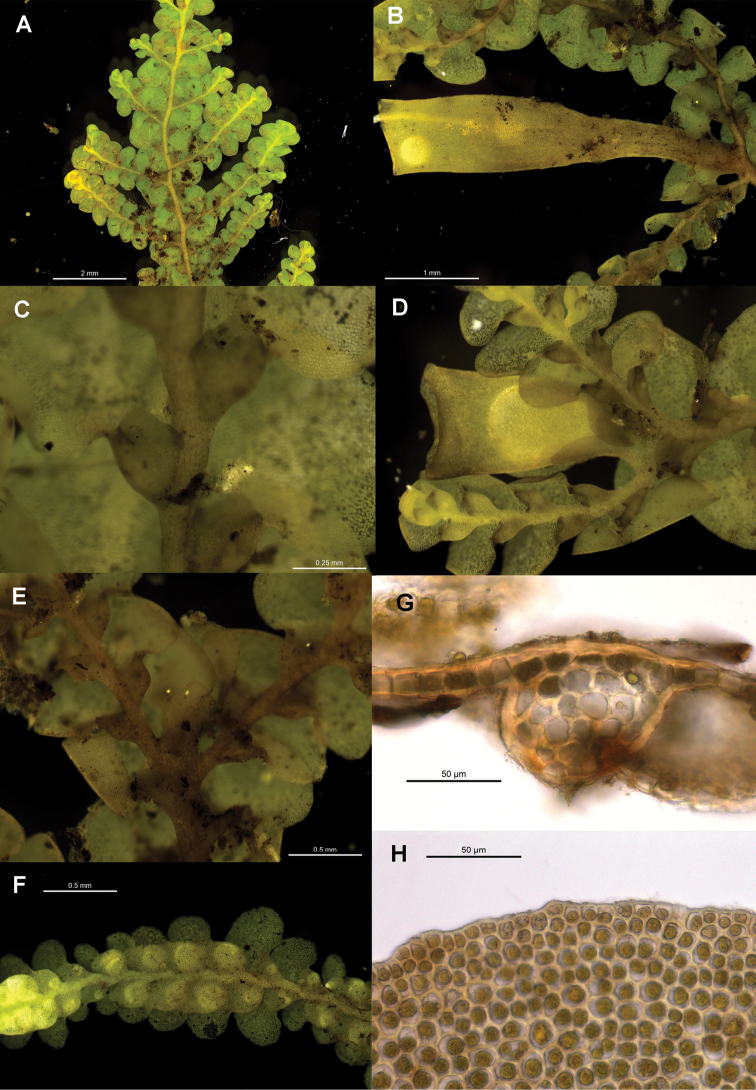
*Radula demissa* pictures. **A** Ventral view of shoot **B** Mature perianth **C** Ventral view of lobules on primary shoot **D** Immature perianth showing inrolled labia **E** Gynoecium **F** Androecium **G** Transverse sections of stem from primary shoot **H** Leaf-lobe marginal cells. All from NSW895351.

#### Etymology.

From Latin *demissa*, hanging, in reference to the hanging fan shaped wefts formed by this species when growing on twigs and branches, also in reference to the frequent colonisation of leaves of the fern *Hymenophyllum demissum*.

#### Distribution and habitats.

South-eastern Australia in Victoria and Tasmania, and New Zealand. On mainland Australia *Radula demissa* occurs only on tablelands and highlands in southern and eastern Victoria, but in Tasmania the species is widespread. *Radula demissa* grows in cool temperate and warm-temperate rainforests and occasionally in wet sclerophyll forests, particularly in the southern part of its range.

In New Zealand *Radula demissa* occurs throughout all three main islands, and extends east to the Chatham Islands and south to the Auckland and Campbell Islands. It inhabits all forest environments, but is rare in coastal forests with high exposure to salt-laden winds. *Radula demissa* occupies a broad elevation range, from the coast to around 1600 m asl, where it grows in subalpine scrub above the treeline.

In forests *Radula demissa* is typically an epiphyte on tree trunks, branches and twigs where it forms characteristic fan-shaped hanging wefts, and is frequently encountered in riparian areas and edge habitats where local light environments are brighter than adjacent forest interiors. Common host species in New Zealand include *Melicytus ramiflorus* and *Beilschmiedia tawa*. *Radula demissa* is also commonly encountered as an epiphyll on the leaves of ferns and broadleaved shrub and tree species. In New Zealand *Hymenophyllum* species, including *Hymenophyllum demissum*, *Hymenophyllum ferrugineum* and *Hymenophyllum scabrum* are favorite fern hosts, and *Beilschmiedia tawa* is a frequent broadleaf host. In lowland to montane podocarp-broadleaf forest of tall stature with sparse sub-canopy and shrub layers *Radula demissa* can be found as a trunk epiphyte and epiphyll on *Beilschmiedia tawa*, and as an epiphyll on leaves of *Hymenophyllum demissum* where this species grows in dense carpets on the forest floor. On bark *Radula demissa* is often associated with *Radula allisonii*, *Radula plicata*, *Radula strangulata*, *Metzgeria* spp. and various species of Lejeuneaceae including *Drepanolejeunea aucklandica* and *Metalejeunea cucullata*, Sematophyllaceae, various Plagiochilaceaespecies including *Dinckleria pleurata*, *Frullania* spp. Co-occurrence with phenetically similar species can pose a challenge to identification. On twigs *Radula demissa* grows with *Plagiochila colensoi*, *Lepidolaena taylorii*, *Lepidolaena palpebrifolia*, *Radula plicata*, and on leaves *Radula demissa* grows with *Echinolejeunea papillata*, *Cololejeunea laevigata*, and members of the *Lejeunea epiphylla* Colenso aggregate.

In subalpine scrub *Radula demissa* occurs on damp rock and adjacent soil, sometimes in highly insolated microsites. Alpine plants exhibit some subtle morphological differences from forest plants, as discussed in the next section.

#### Variation.

Included within *Radula demissa* are forms that grow on wet rocks in alpine habitats, typically between 800 and 1600 m asl. Alpine forms exhibit subtle morphological differences from forest plants. The leaves are remote to contiguous rather than imbricate, and the lobes posses a small auricle at the dorsal base of the stem insertion, which is not found in forest plants. The carinal region is typically narrowly inflated along the length of the keel, rather than more broadly inflated across the width of the lobule. Although alpine plants expressing these features are known from several sites in New Zealand, alpine plants are not currently known from Tasmania or mainland Australia.

The existence of subtly different, ecologically distinct, alpine plants of *Radula demissa* could be explained in several ways. Alpine plants could be an ecotype within a reproductively cohesive species that also occupies forests. They could be a partially reproductively isolated population that retains genetic contact with forest populations. They could be fully reproductively isolated, morphologically cryptic species. These alternatives remain untested. Alpine accessions nest within *Radula demissa* in our phylogeny, but may warrant investigation through additional genetic, ecological, and physiological data. Given the species straddles what are effectively different biomes (forest and alpine environments: Crisp et al. 2009) the transition from forest to alpine environments concomitant with a change in microsite occupancy could represent a striking example of phenotypic plasticity.

#### Recognition.

Despite being confused with a range of related and unrelated species, *Radula demissa* is easy to recognise. The first clue to identity comes from the habitat and microhabitat the plants occupy. *Radula demissa* is typically an inhabitant of well-lit microsites, and is an epiphyte or epiphyll in forest, and a lithophyte in subalpine shrublands and grasslands. Three other species of the *Radula buccinifera* complex occupy similar microsites across the range of *Radula demissa*, and may co-occur with it.

In Australia, *Radula buccinifera* may co-occur with *Radula demissa* on tree trunks. Differentiating these two species is only possible on the basis of hydrated, slide-mounted material because deformation associated with drying renders void examination and interpretation of leaf-lobe and lobule shape and orientation in dry material. The most accessible character by which *Radula demissa* and *Radula buccinifera* differ is in the orientation and spacing of the leaf lobes, however differences are not as clear cut as between other pairs of species (i.e. between *Radula demissa* and *Radula strangulata*) and this character may not prove reliable in all instances. In *Radula demissa* the leaves are contiguous to imbricate, and obliquely patent, spreading up and away from the substrate so that they overlap across the dorsal stem surface, such that the dorsal stem surface is not usually visible from above. In *Radula buccinifera* the leaves are patent to obliquely patent, and while they may spread upward away from the substrate, do not usually overlap completely across the dorsal stem surface so the stem can usually be seen from above, at least in part. However, as there is variation in both leaf lobe orientation and how falcate the lobes are in both species, this character should be used as a guide only. Leaf shape provides another useful clue to identity. In *Radula demissa* the leaves are falcate, with a distinct notch at the junction of the lobe and keel. In *Radula buccinifera* the leaves are not or only weakly falcate, and the junction between the lobe and keel forms a simple angle. Lobule shape is also a good source of diagnostic differences, though the differences between these two species are subtle. For convenience, some conceptual re-orientation of the shoot is required to describe these differences clearly. Imagine the shoot is being held vertically with the apex top-most. In this orientation the lobule apex of *Radula demissa* always lies well above the uppermost point of the ampliate portion of the lobule margin. Between these two points the lobule margin is shallowly S-shaped, and this slopes downward at c. 45° toward the stem. In *Radula buccinifera* the lobule apex lies variably between the same level as, or slightly above, the uppermost point of the ampliate portion of the lobule margin. Between these two points the lobule margin varies from straight to S-shaped in situ (straight when flattened), but when S-shaped there is a pronounced medial curve. The slope of the antical margin varies between sloping downward toward the stem at up to 45° and remaining level. Several shoots should be examined to gauge the patterns of variation in lobule shape, and comparison with known material is recommended.

The lobule keel differs between *Radula demissa* and *Radula buccinifera*. In *Radula demissa* the keel is curved, on lobules from both main shoots and branches. In *Radula buccinifera* the keel is typically straight, but may be curved on some lobules, particularly those on branches.

If these more accessible characters prove ambiguous, diagnostic differences can be derived by counting the number of rows of dorsal cortical cells that are not crossed by the leaf insertion lines. In *Radula demissa* no rows are leaf-free, whereaszero to three rows are leaf-free in *Radula buccinifera*. To achieve this count, leaves must be removed from the stem in such a way as to preserve the leaf insertion yet make the dorsal stem surface visible, which is a challenging proposition at the best of times.

*Radula demissa* may co-occur with *Radula strangulata* on tree trunks and on living leaves close to the forest floor. For guidance on separating these two species on morphological grounds see the recognition section of *Radula strangulata*.

*Radula demissa* has been confused with the New Zealand endemic *Radula plicata*. *Radula demissa* may co-occur with *Radula plicata* on tree trunks, branches, and leaves. Although *Radula plicata* is not closely related to *Radula demissa*, confusion may arise due to the similarity in size and shape of lobes and lobules, in the curved keel with inflated carinal region, the S-shaped antical lobule margin, and the falcate leaf lobes. When fertile female plants are encountered *Radula plicata* is readily separated by the plicate perianth surfaces. However, sterile and male herbarium material may cause problems if gross morphological characters are relied on for identification. *Radula demissa* is readily separable from sterile and male material of *Radula plicata* by its unornamented leaf-lobe cell surfaces, and bulging, rather than crenulate leaf-lobe margin. In *Radula plicata* the leaf-lobe cells bear conspicuous coarse granular ornamentation on their surface. This is easily observed in hydrated slide-mounted material on the lobe marginal cells. The cells of the lobe margin are also distinctly crenulate, although comparison with known material is recommended as a guide to the degree of difference between these and the bulging cells of *Radula demissa*. Differences in stem anatomy also differentiate these two species. In *Radula demissa* the cortical stem cell walls are brown-pigmented, and the medulla walls are yellow-pigmented. In *Radula plicata* the cortical and medulla stem cells walls are unpigmented. If living material is being examined, diagnostic differences may be found in the oil-bodies. In *Radula demissa* there are 2–3 large, light brown, internally homogeneous oil-bodies that have a granular surface and completely fill the cell lumen of each cell. In *Radula plicata* there are 3–5 small, clear or grayish oil bodies with a smooth surface each containing a hyaline droplet, that are arranged in a loose submarginal ring and do not fill the lumen in each cell.

In alpine environments, *Radula demissa* may be confused with *Radula australiana*. However, these two species are readily separated by differences in lobe shape that, with care, can be accessed in the field. In *Radula demissa* the leaf lobes are falcate, with a notch at the lobe-lobule junction. In *Radula australiana* the leaf lobes are not falcate, rather the lobule keel runs more or less seamlessly into the lobe outline. Lobule size and shape also differs between these two species. In *Radula demissa* the lobules are rhombic to widely rhombic and one eighth to one sixth the lobe area. In terms of the same conceptual re-orientation described above, the lobule apex of *Radula demissa* lies above the ampliate inner lobule margin. In *Radula australiana* the lobules are quadrate and one sixth to one quarter the lobe area, with the lobule apex lying level with the ampliate inner lobule margin. Furthermore, the ampliate portion of the lobule is typically much larger, and the lobule apex more acute in *Radula australiana* than in *Radula demissa*.

Perianth morphology may lead to confusion between *Radula demissa* and *Radula ratkowskiana*, particularly given comments by Renner (2005) that inrolling perianth labia and the associated bicornute perianth is unique to *Radula ratkowskiana*. This is not the case, both features may be developed in young perianths of *Radula demissa*, which can be distinguished from *Radula ratkowskiana* by the small rhombic lobules, and the absence of dome-shaped papillae on the leaf lobe cells.

#### Remarks.

Mitten (1855) was the first to record *Radula buccinifera* for New Zealand, citing collections by Colenso, Stephenson and Lyall. Hodgson (1944) evidently followed Mitten in accepting *Radula buccinifera* for New Zealand, but in the absence of an examined type, equated *Radula demissa* with *Radula buccinifera*. Hodgson’s (1944) description of *Radula buccinifera* having glossy dried plants with falcate leaf lobes whose margins extend over the stem and rhomboid lobules agrees with *Radula demissa*, and on the basis of these characters Hodgson accurately distinguished *Radula demissa* (as *Radula buccinifera*) from *Radula strangulata* (as *Radula levieri* for which she had examined type material). Renner (2005) followed Hodgson’s (1944) conception of *Radula buccinifera*, and the plants presented in his key as *Radula buccinifera* also correspond to *Radula demissa*. This error was made despite the fact that he had examined syntype material of *Radula buccinifera* held in bm in early 2004, apparently the diagnostic differences in leaf lobe and lobule shape were overlooked. As discussed under *Radula buccinifera*, Mitten’s New Zealand record of that species must be rejected.

In Australia *Radula demissa* has remained unrecognized, being subsumed within an increasingly broad working circumscription of *Radula buccinifera*, as discussed under that species.

Colenso’s description of a bipinnately branched plant with dimorphic shoots, ‘peduncled’ perianths with a truncate mouth with slightly uneven labia, and subtended by two divergent subfloral innovations, growing epiphytic on *Hymenophyllum* is consistent with *Radula demissa*. However, no definite type material for Colenso’s *Radula epiphylla* has been located, and our attribution of this invalid name to synonymy under *Radula demissa* is tentative.

#### Specimens examined.

New Zealand, North Island: Te Paki Ecological Region and District, Te Paki, Radar Bush, 34°28'03"S, 172°51'15"E, 160 m, 19 Sep 2011, *P.J. de Lange 9991 & M.A.M. Renner*, NSW970835; Northland Ecological Region, Maungataniwha Ecological District, Mangamuka Range, 35°10'S, 173°24'E, 200 m, 17 Apr 1984, *J.E. Braggins 84/26h*, AK312107; Maungamuka Stream headwaters, 35°11'S, 173°29'E, 210 m, 30 Nov 1986, *J.E. Braggins 86/304*, AK259001; Auckland Ecological Region, Hunua Ecological District, Kohukohunui Track c. 1 km west of Kohukohunui summit, 37°02'S, 175°13'E, 630 m, 21 Aug 2000, *M.A.M. Renner 00/51*, AK280339; Coromandel Ecological Region, Te Aroha Ecological Distric, Mt Te Aroha summit, Dog Kennel Flat track, 37°32'S, 175°45'E, 940 m, 12 Mar 1995, *J.E. Braggins 95/213C*, AK255288; Rotorua, Blue Lake, May 1953, *E.A. Hodgson 10382*, BM;Puaiti Bush south of Rotorua, 23 May 1931, *K.W. Allison*, CHR587333; Near Atiamuri north of Waikato River south of Rotorua, 6 Sep 1929, *K.W. Allison*, CHR587334; Roto-a-kia Bush, east of Taupo, southern Kaingaroa Plains, 18 Sep 1935, *K.W. Allison*, CHR587330; near Atiamuri, south of Rotorua, Aug 1930, *K.W. Allison*, CHR587331; Urewera Ecological Region, Waikaremoana Ecological District, Lake Waikaremoana, Whanganuioparua Inlet, Te Kumi Stream, 38°45'S, 177°9'E, 700 m, 19 Jan 2001, *M.A.M. Renner 01/27*, AK280333; Central Volcanic Plateau Ecological Region, Taupo Ecological District, Pureora Forest, northern end of Waihora Lagoon, 38°39'S, 175°40'E, 640 m, 27 May 1988, *J.E. Braggins 88/020B*, AK258496; Eastern Volcanic Plateau Ecological Region, Kaingaroa Ecological District, Poronui Valley, Mangatamingimingi Stream, 38°59'S, 176°16'E, 725 m, 8 Apr 2003, *J.E. Braggins & M.A.M. Renner 00/61*, AK280574, WELT-H011593, CANB738633.1;Waipapa Ecological Area, Pureora Forest Park, 38°26'S, 175°35'E, 565 m, 26 Jan 1982, *J.E. Braggins et al.*, AK291246; Egmont Ecological Region and District, North Egmont Tourist track and Nature walk, 39°16'S, 174°5'E, 960 m, 12 Sep 1999, *J.E. Braggins 99/219C*, AK253449;Mt. Ruapehu, off Ohakune Mountain Road, downhill of 7 km marker, 39°22'S, 175°28'E, 890 m, 27 Nov 1992, *J.E. Braggins 92/91*, AK312261; Hawkes Bay, Morere Hotel, Morere, on tree bark in dense bush, 21 Aug 1964, *R.E. Hatcher 2*, F;Edge of forest by road, Lake Waikaremoana, 2000 ft, Jan 1955, *E.A. Hodgson*, ex herb. E.A. Hodgson 11207, S-B89674 as *Radula buccinifera*; Egmont Ecological Region and District, Mt Taranaki, Egmont National Park, track to skifield from Pembroke Road, 39°18'S, 174°05'E, 1260 m, 11 Dec 1999, *J.E. Braggins 99/317*, AK254565; *Colenso s.n.*, ex herb Steph. in FH as *Radula plicata*; South Island: Whataroa Ecological Region, Hokitika Ecological District, Lake Kaniere, Slip Bay, 42°52'S, 171°10'E, 200 m, 25 Nov 1995, *J.E. Braggins 95/637B*, AK285658;Arthur'S, Pass National Park, *Nothofagus solanderi* Wald am Bridal Veil Fall Nature Walk bei Arthur's Pass Village, auf Fels am Bach, 800–850 m, 5 Feb 1991, *Schäfer-Verwimp & Verwimp*, Herb. Schäfer-Verwimp 14336; Westland Land District, Otira River, Arthurs Pass, 730 m, 7 Apr 2004, *T. Hay*, CHR583489; Haast, 3 miles north of bridge, 30 m, 4 Mar 1972, *J. Child*, CHR453978; Otago Coast Ecological Region, Dunedin Ecological District, Dunedin City, Leith Valley, Morrisons Burn, 45°50'S, 170°30'E, 220 m 20 Nov 1998, *J.E. Braggins 98/335*; AK253710; Marlborough, Pelorus River catchment, head of Elvy Stream, 41°18'52"S, 173°34'24"E, 270 m, 12 Feb 2012, *M.A.M. Renner 6076*, NSW895351; South Westland, Haast Pass, Cross Creek, 44°05'50"S, 169°21'31"E, 560 m, 15 Feb 2012, *M.A.M. Renner 6127*, NSW895397; South Westland, Haast Pass, track to Brewster Hut, 44°04'49"S, 169°23'24"E, 660 m, 15 Feb 2012, *M.A.M. Renner 6137*, NSW895439; Westland, Chesterfield, Kapitea Creek, 42°37'20"S, 171°07'29"E, 35 m, 17 Feb 2012, *M.A.M. Renner 6180*, NSW895508; Westland, Chesterfield, Kapitea Creek, 42°37'20"S, 171°07'29"E, 35 m, 17 Feb 2012, *M.A.M. Renner 6183*, NSW895511; Westland, Paparoa National Park, Fox River, 42°02'26"S, 171°23'58"E, 20 m, 18 Feb 2012, *M.A.M. Renner 6227*, NSW895686; Nelson, Kahurangi National Park, Cobb Valley, Round Lake cirque, 41°03'08"S, 172°30'03"E, 1410 m, 19 Feb 2012, *M.A.M. Renner 6241*, NSW896177; Nelson, Kahurangi National Park, Cobb Valley, Round Lake cirque, 41°03'08"S, 172°30'03"E, 1410 m, 19 Feb 2012, *M.A.M. Renner 6244*, NSW896179;

Chatham Islands: Chatham Island, Waitangi, Crispans Lane, Unnamed Stream, 43°57'S, 176°33'E, 10 m, 9 Jan 2006, *P.J. de Lange CH784 & J.W.D. Sawyer*, AK299897;

Australia: Victoria: Otway Range, Carslisle State Park, Carlisle-Gellibrand Road, 38°32'08"S, 143°29'16"E, 11 Jun 1996, *D.A. Meagher* *s.n.*, MEL2053881; Eastern Highlands, Warburton, 37°42'S, 145°42'E, 28 Feb 1902, *R.A. Bastow*, MEL1037783.

Tasmania: New Norfolk Municipality, tributary of the Styx River 10 miles west of Maydena, 146°32'E, 42°46'S, 4 Dec 1973, *D. Norris 28952*, F; Kentish Municipality, cliffs above Lake Barrington near Forth Falls, c. 150 m, 41°24'S, 146°11'E, 15 Nov 1973, *D. Norris 27306*, F; Upper North West Bay River, 42°55'S, 147°12'E, 12 Feb 1980, *A.V. Ratkowsky H1137*, HO304430, as *Radula plicata* det M.-L. So; Mt. Wellington, Upper N.W. Bay River, 42°55'S, 147°13'E, 12 Feb 1980, *A.V. Ratkowsky*, CANB8205585, as *Radula plicata* det Yamada; South West, South West Conservation Area, Huon River, adjacent Huon campground, 43°02'17"S, 146°18'12"E, 285 m, 23 Jan 2012, *M.A.M. Renner 5936 & E.A. Brown*, NSW895267; South West, South West National Park, Mount Eliza, track to summit from Scotts Peak Road, 100 meters above Eliza Hut, 42°57'38"S, 146°24'16"E, 1000 m, 22 Jan 2012, *M.A.M. Renner 5916 & E.A. Brown*, NSW909267; South West, South West Conservation Area, Mount Eliza, Condominium Creek, 42°57'26"S, 146°21'49"E, 350 m, 23 Jan 2012, *M.A.M. Renner 5923 & E.A. Brown*, NSW895246; South West, South West Conservation Area, Huon River, adjacent Huon campground, 43°02'17"S, 146°18'12"E, 285 m, 23 Jan 2012, *M.A.M. Renner 5940 & E.A. Brown*, NSW895272; South West, Princess Creek catchment, east side of Lyell Highway between stream and road, 42°08'40"S, 145°29'18"E, 325 m, 26 Jan 2012, *M.A.M. Renner 5989 & E.A. Brown*, NSW909286; South West, Mount Dundas Regional Reserve, Manuka River, between Lyell Highway and river, 42°08'36"S, 145°22'56"E, 195 m, 26 Jan 2012, *M.A.M. Renner 5998 & E.A. Brown*, NSW909293; West Coast, Waratah-Savage River Road, Arthur River catchment, unnamed stream, 41°27'52"S, 145°25' 26"E, 490 m, 28 Jan 2012, *M.A.M. Renner 6023 & E.A. Brown*, NSW909423; West Coast, Waratah-Savage River Road, Arthur River catchment, unnamed stream, 41°27'53"S, 145°25'22"E, 540 m, 28 Jan 2012, *M.A.M. Renner 6024 & E.A. Brown*, NSW909424; West Coast, Williamsford Road, Ring River, 41°49'33"S, 145°30'41"E, 405 m, 31 Jan 2012, *M.A.M. Renner 6036 & E.A. Brown*, NSW909452; West Coast, Williamsford Road, Ring River, 41°49'33"S, 145°30'41"E, 405 m, 31 Jan 2012, *M.A.M. Renner 6048 & E.A. Brown*, NSW909482.

### Radula imposita

M.A.M.Renner sp. nov.

http://species-id.net/wiki/Radula_imposita

Figs 17 –18

#### Diagnosis.

Similar to both *Radula buccinifera* and *Radula demissa* and could easily be mistaken for a poorly developed morph of either, but differs in the crenulate leaf-lobe margins and low dome-shaped papillae over each leaf-lobe cell. Differs also in the relatively small size, the densely but irregularly pinnate branching pattern, and the irregular margins to the female-bract lobes, which are also crenulate.

#### Type.

Australia: Queensland: Cook, Daintree National Park, Mount Lewis, headwaters of Leichhardt Creek flowing down the south-west flanks of the summit, epiphyllous on *Normandia* frond overhanging stream, 1150 m, 16°35'03"S, 145°16'33"E, 27 May 2012, *M.A.M. Renner 6356, V.C. Linis & E.A. Brown* (holotype: NSW896812; isotype: BRI).

#### Description.

[From NSW896812] Forming tufts of overlapping and interlocking shoots on leaves and twigs, bright- to mid-green when fresh, fading to a glossy tan or brown in herbarium; shoot systems densely irregularly pinnately branched in female plants, with additional pseudodichotomous branching in fertile female plants due to production of pairs of subfloral innovations below gynoecia; monomorphic, 0.8–1.2 mm wide and up to 30 mm long, branches smaller in stature than parent shoot; older shoot sectors retaining leaf-lobes. Stems 80–130 µm diameter, with cortical cells in a single tier of 15–30 rows; cortical cell walls yellow-brown to brown pigmented; external free cortical cell wall heavily and continuously thickened, radial longitudinal cortical walls thickened or not, inner tangential wall more or less continuously thickened; medulla cells in 10–25 rows; cell walls yellow-brown pigmented, with medium-sized triangular to weakly bulging trigones, walls between trigones lacking thickenings or continuously thickened; cortical cells on dorsal stem surface arranged in straight longitudinal rows on young and mature shoot sectors. Leaf insertion reaching dorsal stem mid-line or not, leaving zero or one dorsal cortical cell rows leaf-free, in some shoots stem growth appears to have introduced an additional cortical cell row to the dorsal stem surface, in which case a single row of dorsal cortical cells is leaf-free, but this row is discontinuous between adjacent leaf pairs; leaf insertion not attaining the ventral stem mid-line, leaving two to four ventral cortical cell rows leaf-free. Leaf lobes elliptic-ovate, 430–700 µm long by 320–450 μm wide, larger on primary shoots, contiguous to imbricate, not to weakly falcate, acroscopic base not sharply deflexed away from stem, flat, patent, not interlocking over the dorsal stem surface, stem visible between leaf lobes in dorsal view; margins irregularly but minutely repand, crenulated due to medial wall thickenings on marginal cell walls, the interior lobe margin weakly ampliate, covering part of the dorsal stem surface but not often reaching the stem midline, and never exceeding the opposite stem margin, antical margin curved, exterior margin curved, postical margin curved to straight; angle between postical lobe margin and keel 45-60°. Lobules rhombic when small to almost quadrate, one sixth the lobe area, 175–345 µm long by 150–265 μm wide, larger on leading shoots; keel straight to curved, angle between keel and stem 135°, keel apex and postical lobe margin running into lobe margin smoothly or shallowly notched; interior lobule margin free for one eighth to one sixth its length, free portion not or weakly ampliate, not or hardly extending onto the ventral stem surface; acroscopic margin shallowly S-shaped, apical portion inclined towards stem; apex apiculate-rounded; free exterior margin straight to curved, margins plane, entire; lobe-lobule junction level with the acroscopic end of stem insertion in small and large lobules; attached to stem along 0.83 to 0.88 of the interior margin, stem insertion straight to curved, not revolute at acroscopic end; lobule apex bearing a single papilla, with another two papilla situated on the interior lobule margin above the stem insertion. Leaf lobe cells rounded-oblong, not arranged in rows, unequally sized, 11–22 µm long by 8–16 μm wide, walls slightly thickened their entire length, sometimes with small triangular trigones, medial wall thickenings absent; cells of lobe margin smaller than those of leaf middle, quadrate to rectangular, 9–14 µm long and wide, interior walls not differentially thickened, exterior walls with pronounced medial thickening, and medially bulging cell lumen; leaf lobe cell surface papillose, upper lobe wall differentially thickened over cell lumen forming a single low dome-shaped papilla over each cell. Oil-bodies not known. Asexual reproduction not known. Dioicous. Androecia not known. Gynoecia terminal on leading shoots, subtended by two subfloral innovations that are full-sized and again fertile; archegonia 145–160 µm tall, archegonia neck five or six cell columns, 6–8 per gynoecium on a small disc of tissue, encompassed by the protoperianth; female bracts in one pair, slightly asymmetrical with lobule on one larger than the other, imbricate, elliptic-ovate, lobe 520–570 μm long by 240–320 μm wide, margins irregular and crenulate; lobules rectangular-ovate, one half to two thirds the lobe area, apex rounded to obtuse, keel arched, irregular.

**Figure 17.**
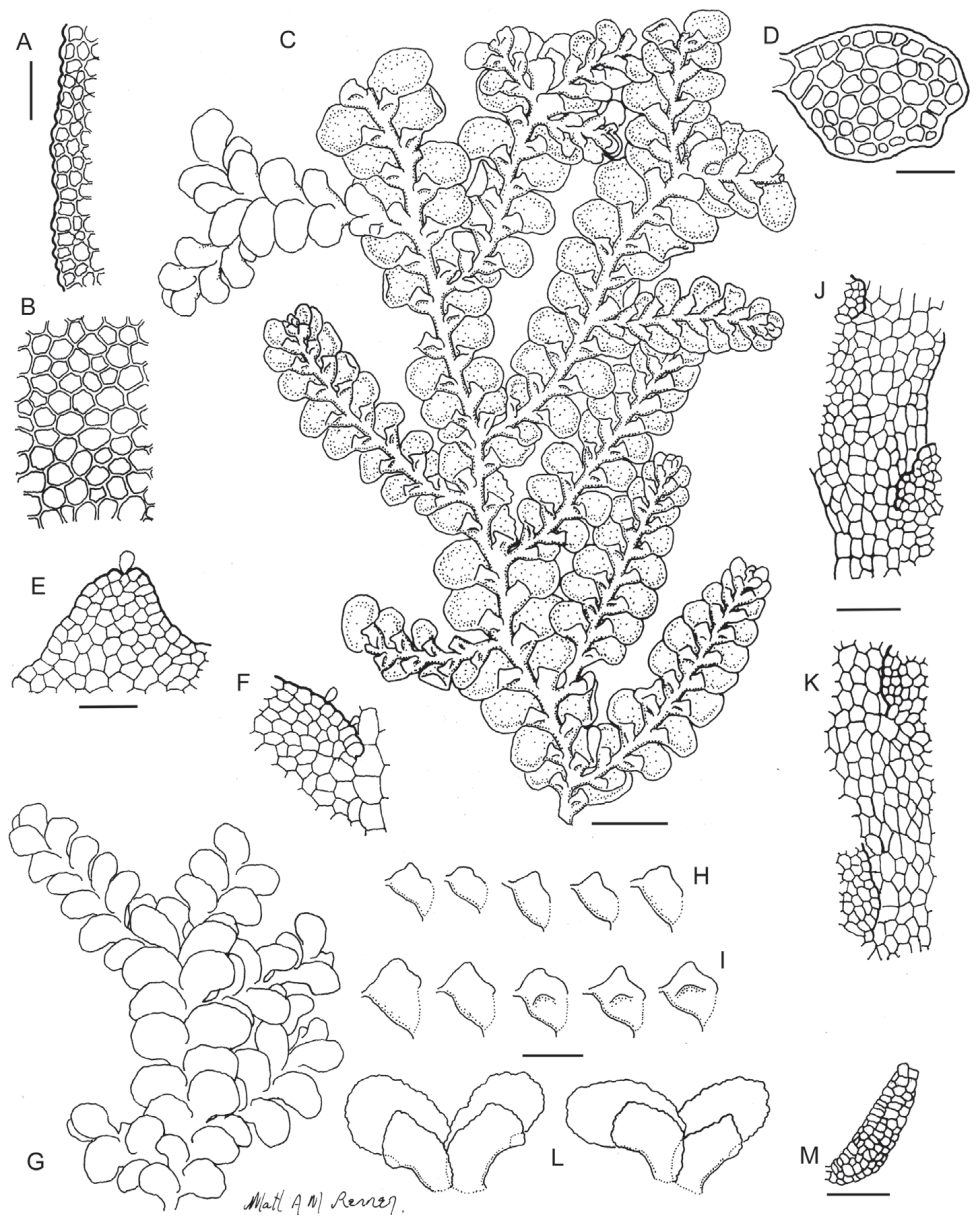
*Radula imposita* Line drawings. **A** Cellular detail of leaf lobe margin **B** Cellular detail of medial leaf-lobe cells **C** Ventral view of shoot **D** Transverse section of stem from primary shoot **E** Cellular detail of lobule apex **F** Cellular detail of lobule interior base **G** Dorsal view of shoot **H** Five lobules from secondary shoots **I** Five lobules from primary shoots **J** Ventral view of stem surface **K** Dorsal view of stem surface, showing leaf-lobe insertion attaining the dorsal stem mid-line **L** Female bracts **M** Archegonium. Scale bars: **A–B, D–F**: 40 µm. **C, G**: 600 µm.: **H, I, L**: 240 µm. **J–K, M**: 60 µm. All from NSW896812.

**Figure 18.**
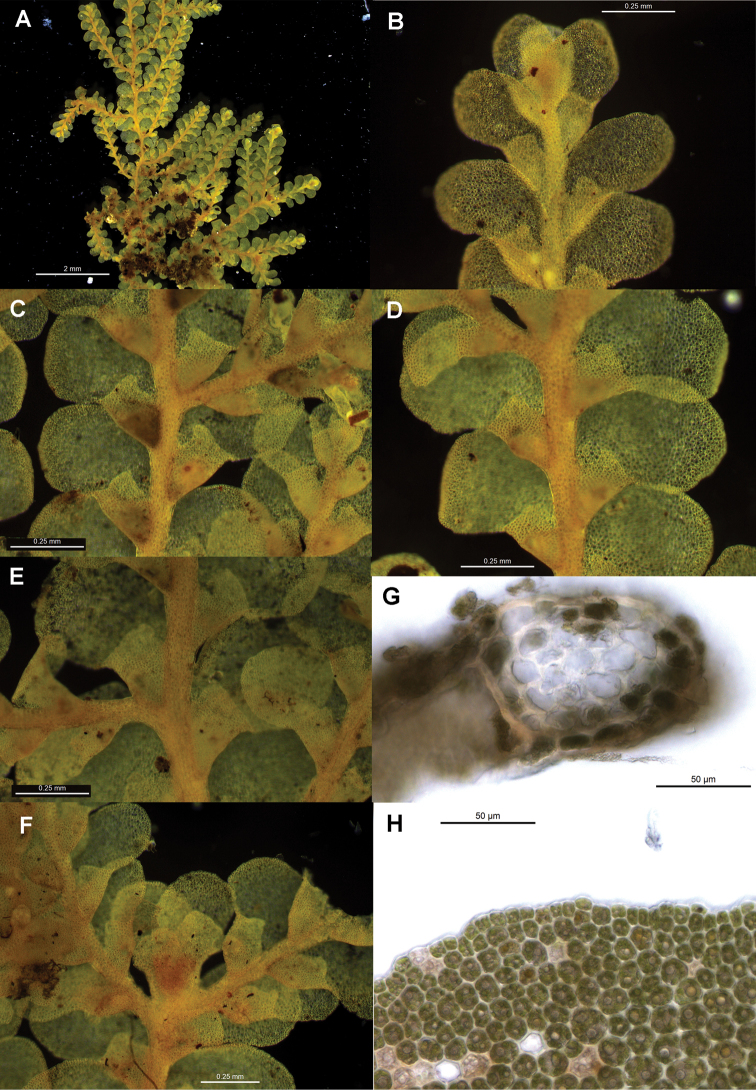
*Radula imposita* pictures. **A** Ventral view of shoot **B** Dorsal view of shoot apex. **C–E** Ventral view of lobules on primary shoots **F** Gynoecium **G** Transverse sections of stems from primary shoot **H** Leaf-lobe marginal cells. All from NSW896812.

#### Etymology.

*Imposita*: laid-upon, in reference to both the epiphyllous habit and the way the small, densely packed branches grow over one another in the type.

#### Distribution and ecology.

Known to date from four specimens, all growing over running streams on either leaves or bark including tree-trunks and branches. The specimens were collected in the North Coast of New South Wales, and in the Wet Tropics Bioregion of north-east Queensland.

#### Variation.

Within its already reduced size, in comparison to its relatives, *Radula imposita* exhibits limited variation in shoot size and lobule morphology within individuals, and much more variation between individuals. However, knowledge of this species is based on only four specimens, from extremes of a geographical distribution spanning 15 degrees of latitude, so more variation than described here should be anticipated.

#### Recognition.

*Radula imposita* could easily be mistaken for a poorly developed morph of either *Radula buccinifera* or *Radula demissa*. The ecological envelope and geographical distribution of both species exhibits some overlap with *Radula imposita*. However, the crenulate leaf-lobe margins and low dome-shaped papillae over each leaf lobe cell are distinctive and will immediately distinguish *Radula imposita* from both *Radula demissa* and *Radula buccinifera*. Several other features are distinctive of *Radula imposita* including the relatively small size, the dense irregularly pinnate branching pattern with almost monomorphic shoots, and the irregular and crenulate margins to the female-bract lobes.

#### Specimens examined.

Australia: New South Wales: North Coast, Dorrigo National Park, Rosewood River, Rosewood Creek Track, Oreocallis Gully, 30°21'58"S, 152°48'12"E, 650 m, 15 Apr 2011, *M.A.M. Renner 5275*, NSW875821; North Coast, Karuah River Road at Karuah River, Chichester State Forest, 26 km SW of Gloucester, 32°07'S, 151°43'E, 380 m, 27 Apr 1990, *H. Streimann 44710*, CANB9010658; North Coast, Myall River State Forest, Strike-a-light camping area, on road leading to ford across Macleans River, 32°17'31"S, 152°05'04"E, 210 m, 5 Apr 2002, *E.A. Brown 2002/18 & B.J. Conn*, NSW491702.

### Radula mittenii

Steph. Hedwigia 23: 148. (1884)

http://species-id.net/wiki/Radula_mittenii

Figs 19 –21

#### Type.

Australia: Zaintree River [orthographical corruption of Daintree River], *Pentzke s.n.* 1882, herb. Mitten (lectotype [designated here]: Element 2, separated as subpacket within NY00831342!)

#### Description.

[From NSW897201] Forming extensive pure sheets of interwoven pendulous shoots on tree trunks and rocks; living plants opaque yellow-green to glaucous brown-green, fading to milky pale-brown in herbarium; shoot systems regularly pinnate, subdimorphic, with shoots 1.6–2.5 mm wide and up to 80 mm long, branches typically slightly smaller in stature than parent shoot; older shoot sectors becoming ragged in appearance due to irregular leaf fragmentation. Stems 190–250 µm diameter, with cortical cells in a single tier of 30–50 rows; cell walls brown pigmented throughout; cortical cell walls heavily and continuously thickened, at times constricting the cell lumen, dorsally arranged in an oblique zig-zag on young shoot sectors, cell elongation somewhat obscuring this pattern in mature shoot sectors; medulla cells in 80–110 rows, cell walls heavily thickened with coarse nodular trigones that become confluent, and constrict the cell lumen, thin walls occasional. Leaf insertion exceeding dorsal stem mid-line, insertion lines interlocking over two dorsal cortical cell rows, dorsal leaf-free strip absent. Leaf lobes oblong-falcate, 770–1360 µm long by 560–870 µm wide, contiguous to weakly imbricate, acroscopic base sharply deflexed away from stem, otherwise leaves weakly convex, not interlocking over the dorsal stem surface, stem visible in dorsal view, margins may be irregular in outline but always entire, the interior lobe margin curved, not auriculate, antical margin shallowly curved, exterior margin broadly rounded, postical margin straight or substraight. Lobules on leading shoots typically one quarter the lobe area, more or less quadrate, 430–720 µm long by 510–750 µm wide, keel straight to shallowly arched, angle between keel and stem 135°, keel turning through 45–55° at the apex; interior free margin weakly ampliate, acroscopic margin straight or shallowly arched, usually more or less perpendicular to shoot axis, and apices obtuse or broadly acute; attached to stem along 0.4–0.5 of the interior margin, stem insertion gently S-shaped but abruptly revolute at acroscopic end; lobule apex bearing a single papilla, with another papilla situated on the interior lobule margin above the stem insertion; lobules on leading shoots typically larger than those on branches, lobules on branches more rhomboid than quadrate, one fifth to one quarter the lobe area, keel shallowly arched to straight to slightly curved, angle between keel and stem 135°, keel turning through 45–55° at the apex, interior free margin weakly ampliate, acroscopic margin typically S-shaped to shallowly arched, inclined to shoot axis, apex typically broadly acute. Leaf lobe cells rotund to rounded-oblong, 19–28 µm long by 15–23 µm wide, thin walled with concave to triangular trigones, medial wall thickenings absent; cells of lobe margin smaller than those of leaf middle, quadrate to rectangular, 10- 15 µm long by 7–11 µm wide, long axis orientated parallel to lobe margin, exterior cell walls each with a medial wall thickening that bulges into the cell lumen margin; cell surface weakly bulging, bearing heavy verrucose ornamentation. Oil-bodies not known. Asexual reproduction by caducous leaf lobes, sporadic, typically but not always on old shoot sectors, fragmentation scars irregular, shoot primordia forming as irregular buds on leaf lobe margins in older shoot sectors prior to leaf fragmentation. Dioicous. Androecia on lateral branches that continue vegetative growth, androecial bracts in 3–5 pairs, smaller than vegetative leaves, lobes 510–660 µm long and 360–430 µm wide, ovate, imbricate, lobules hypostatic, keel deeply curved, bearing 1–2 antheridia each. Gynoecia terminal on axes, with one pair of female bracts subtended by one (on branches) or two (on leading shoots) full sized subfloral innovations that may again be fertile; archegonia 160–200 µm tall, archegonia neck five cell tiers, cells regularly arranged, 18–20 per gynoecium on a small raised disc of tissue encompassed by the base of the protoperianth; female bracts equal, ovate-falcate, lobes 1200–1310 µm long by 685–870 µm wide, lobules ovate, one half the lobe area, apex rounded to obtuse, keel strongly arched, insertion interlocking both dorsally and ventrally, insertion equitant. Perianths 3000–3500 µm long and 800–900 µm wide at mouth, cyathiform, flaring widely from a narrow base, which is a low stem perigynium 4–5 stratose with brown-pigmented walls, broadest immediately above base, straight-sided, gradually tapering to the mouth, which has irregularly sinuous labia; perianth walls 2–3 stratose at base, unistratose above; calyptral perigynium present, unfertilised archegonia ‘riding’ onto base of calyptra, calyptra 2–3 stratose at base, tapering to unistratose above.

**Figure 19.**
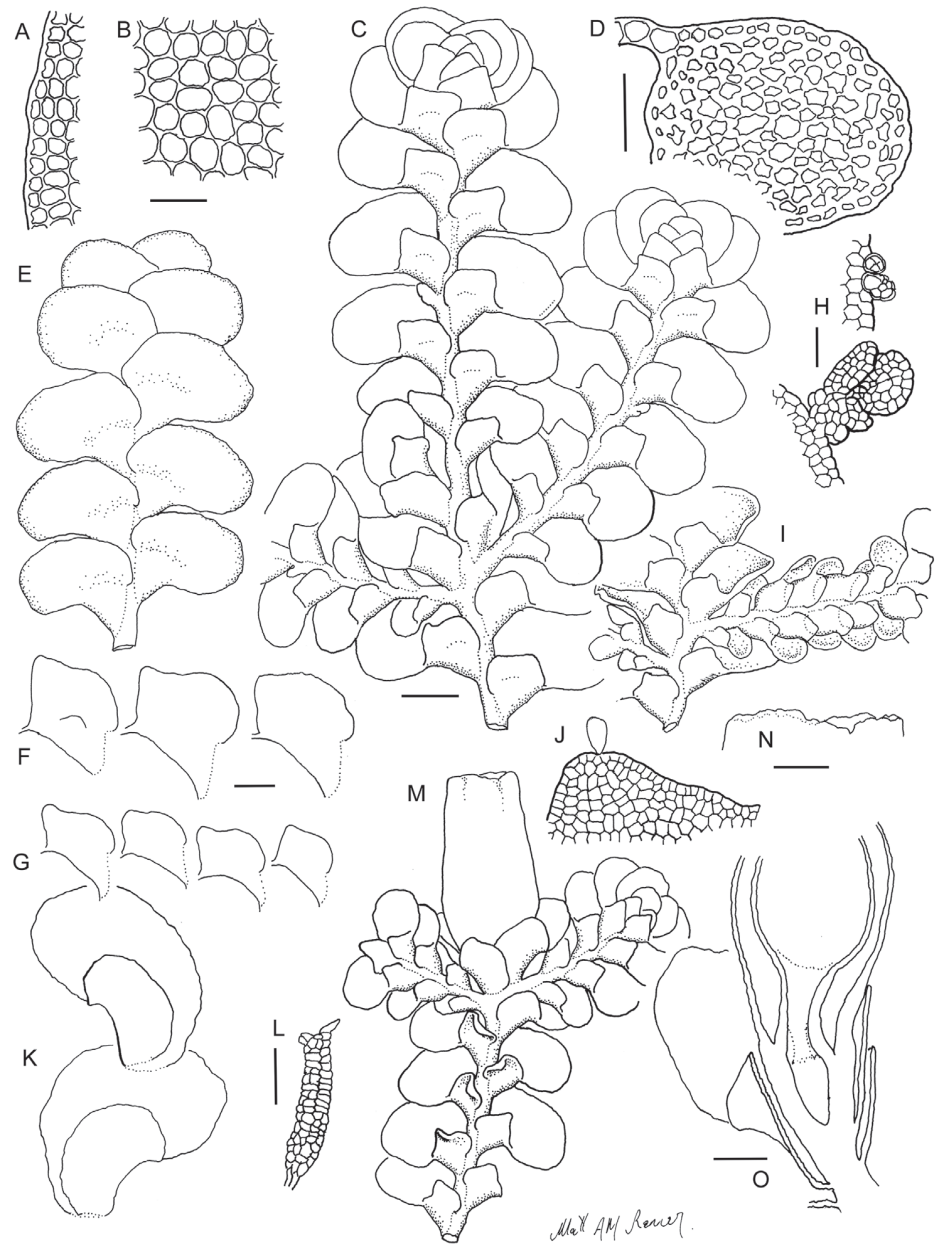
*Radula mittenii* line drawings. **A** Lobe marginal cells **B** Lobe medial cells **C** Ventral view of female shoot **D** Transverse section of stem from primary shoot **E** Dorsal view of primary shoot **F** Three lobules from primary shoots **G** Four lobules from secondary shoots **H** Shoot primordia and regenerating shoot from leaf-lobe margin **I** Ventral view of androecium **J** Cellular detail of lobule apex **K** Female bracts **L** Archegonium **M** Ventral view of perianth-bearing shoot. N. Perianth mouth **O** Transverse section of perianth. Scale bars: **A–B**: 40 µm, **D, J, H, L**: 60 µm. **C, E, I, M**: 600 µm, **F, G, K, N**: 240 µm, **A–H, J** from NSW867314. **I, K–O** from BRI-AQ331973.

**Figure 20.**
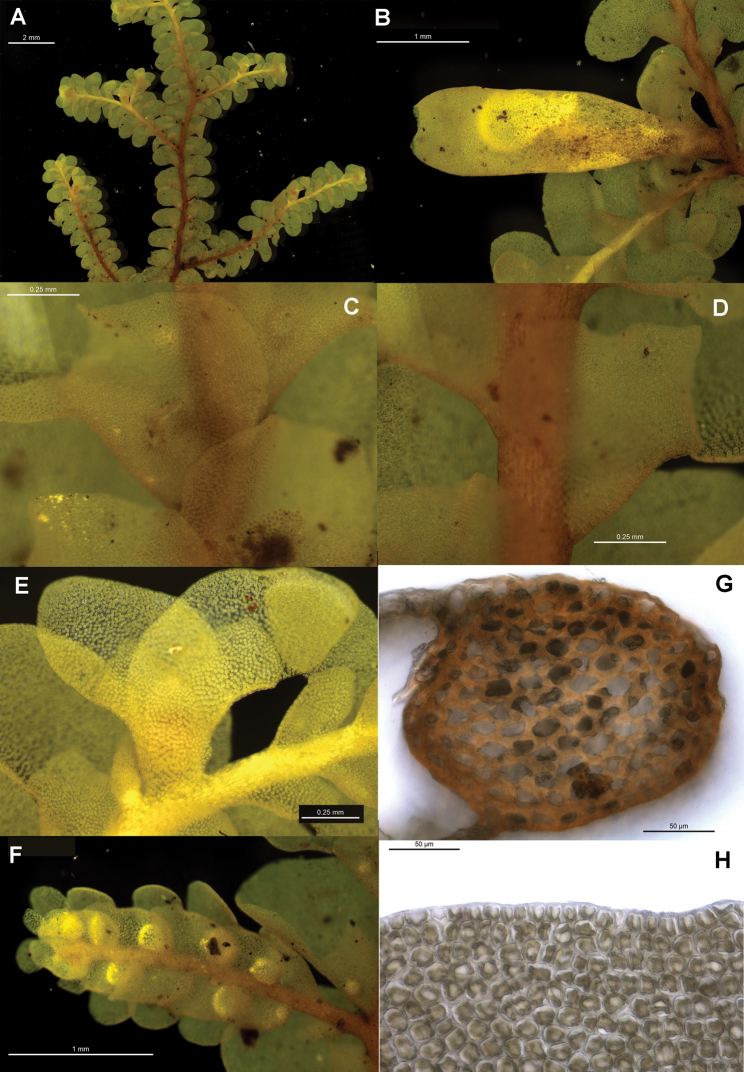
*Radula mittenii* pictures 1. **A** Ventral view of female shoot **B** Immature perianth. **C, D** Ventral view of lobules on primary shoots, C showing maximal extent of imbrication, D showing typical **E** Gynoecium **F** Androecium **G** Transverse sections of stems from primary shoot **H** Leaf-lobe marginal cells. All from NSW897201

**Figure 21.**
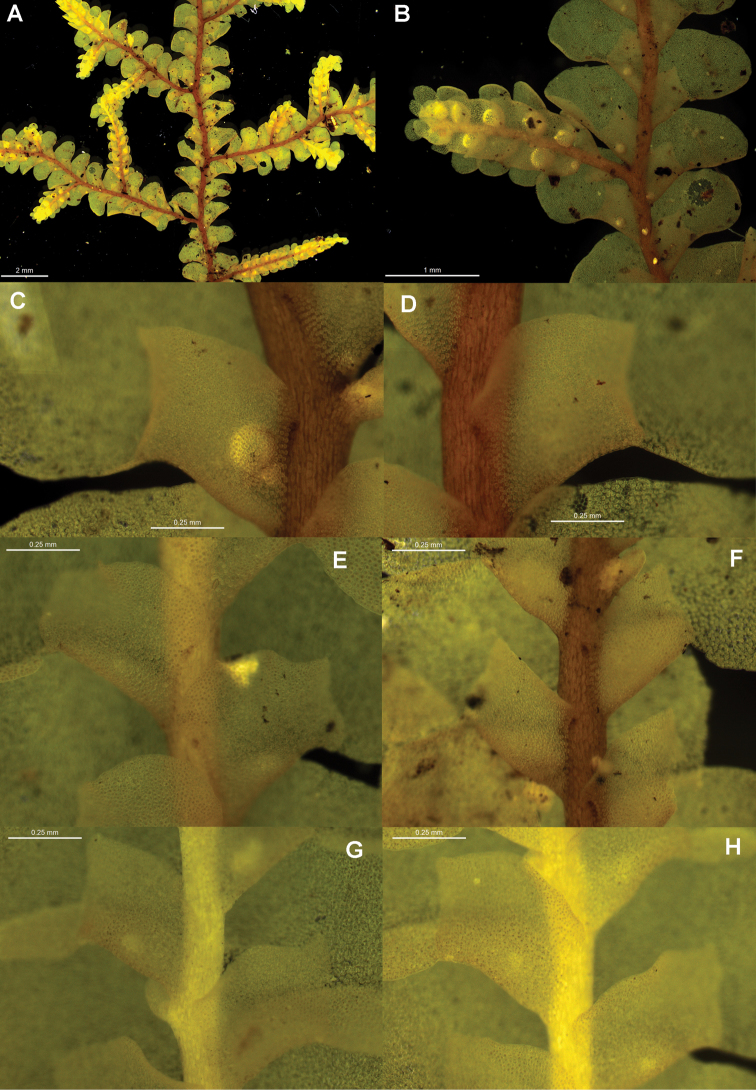
*Radula mittenii* pictures 2. **A** Ventral view of male shoot **B** Androecium on tertiary shoot **C–H** Ventral view of lobules on secondary shoots. All from NSW897201.

#### Etymology.

Named for William Mitten (1819–1906) pharmaceutical chemist and bryologist, contemporary of and collaborator with W.J. and J.D. Hooker, father-in-law to Alfred Russell Wallace; and author of the first floristic treatment of liverworts of New Zealand in Hooker’s Handbook of the New Zealand flora.

#### Distribution and ecology.

In Australia *Radula mittenii* is known from a range of localities in the Wet Tropics Bioregion of north-eastern Queensland. *Radula mittenii* occurs over a broad elevational range, from near sea level to 1500 m, encompassing an array of tropical forest types from lowland mesophyll forests on river floodplains, to cyclone disturbed forests on hillslopes, to notophyll-vine forests on summit peaks. Within these habitats *Radula mittenii* occupies a range of microsites, from boulders, to tree trunks, and branches, twigs, and liane stems. As a lithophyte on the sides of large boulders *Radula mittenii* may form extensive pendulous mats of milky yellow-green shoots.

#### Variation.

Within individuals the dimorphic nature of shoot systems means lobules on primary shoots differ in size and shape from those on secondary shoots, a feature first documented by Renner et al. (in press) that is widespread among species of subg. *Radula*, *Volutoradula* and *Cladoradula*. Lobules on secondary shoots tend toward transverse rectangular shape, and the development of a reflexed antical lobule margin, which causes the lobule apex to appear acute. Plants from exposed situations, including those growing on branches and twigs, may appear to consist only of tangled shoots lacking architectural regularity, bearing lobules of this type, and so differ considerably from the lush, regularly pinnate plants found on rocks and tree-trunks in forest interiors. Close inspection of plants from exposed situations will reveal a primary shoot closely appressed to the substrate from which secondary branches arise. Comparison with plants from sheltered situations will reveal commonalities in the shape and size of lobules from secondary shoots.

#### Recognition.

Among Australasian *Radula*, *Radula mittenii* is easily recognized by its large size, regularly pinnate branching, more or less quadrate lobules, verrucose ornamentation on leaf-lobe cell surface, and the leaf-insertion interlocking over the dorsal stem mid-line. highly distinctive, and easily recognized species. The outstanding feature of *Radula mittenii* is the verrucose ornamentation that it imparts a milky yellow appearance to plants in life and in herbaria. However, colour is not always a reliable guide to identity.

*Radula mittenii* shares with *Radula sumatrana* Steph. A number of characters, including shoot size and branching architecture, the verrucose cuticle, and the large more or less quadrate lobules, and the two are probably closely related. However, the type of *Radula sumatrana* (G00112217!) differs in that the lobules tend to become much larger, imbricate, and obscure the stem surface in ventral view. This is consistent with other specimens seen from Borneo.Yamada describes stem cell walls of the type of *Radula sumatrana* as being thin-walled with large trigones. Thin walls in the stems of Australian material examined are rare, and in general trigones are more confluent and less discrete than illustrated by Yamada (1974). *Radula sumatrana* occurs throughout South East Asia from Thailand to Indonesia including Sumatra, Java and Borneo (Yamada 1979), and plants corresponding to it do not occur in Australia. However, in the unlikely circumstance that these two names do refer to the same entity, *Radula mittenii* has priority.

Some forms of *Radula mittenii* bear resemblance to the type specimen of *Radula similis* (G00042714!) in their lobule shape and smaller stature. However, *Radula similis*, a taxon from New Caledonia,has apparently smooth leaf lobe cell surfaces, so is readily distinguished on this basis. However, granular ornamentation in some species of subg. *Radula* can be very difficult to detect with some light microscopes, and we may have missed this feature. At any rate if ornamentation is present, it is not as pronounced as in *Radula mittenii*.

*Radula mittenii* canbe differentiated from members of the *Radula buccinifera* complex on the basis of many characters, including 1) the verrucose lobe cell surfaces, in *Radula buccinifera* the lobe cell surfaces are smooth or bear low dome-shaped papillae; 2) the presence of heavy, continuous, irregular, brown-pigmented secondary thickenings on the medullar cell walls of the stem formed by fusion of nodular trigones, in *Radula buccinifera* the medullar walls are pigmented or not, and if secondarily thickened bear trigones that rarely fuse to form irregular continuous thickenings, 3) the large quadrate lobules on leading shoots one quarter the lobe area, in *Radula buccinifera* complex the lobules are one eighth to one fifth the lobe area and are rhomboid, not quadrate; 4) the production of caducous leaves, all members of the *Radula buccinifera* complexbar *Radula anisotoma* lack specialised means for asexual reproduction; 5) the regular pinnate, subdimorphic branching of female plants, in the *Radula buccinifera* complex female plants are irregularly or pseudodichotomously branched, as a function of patterns of subfloral innovation production (male plants may be pinnately branched).

*Radula mittenii* could be confused with a number of related species that occur in the wet tropics of north Queensland, however as the identity of several of these has not yet been fully resolved, the differentiation of *Radula mittenii* from these species will be dealt with in a subsequent treatment of *Radula reflexa*.

#### Nomenclature.

In the protologue of *Radula mittenii* Stephani states *Radula mittenii* is ‘Dioicous, robust 4–5 cm long, olivaceous, rigid, regularly pinnately branched, branches remote, short. Leaf-lobes obliquely patent, contiguous, subrotund, concave, apices inflexed, dorsally longitudinally adnate, subauriculate… [lobule insertion] long decurrent, lobule base inflated, exterior margin truncate’, and in notes states that “Die Form der lobuli ist fast genau die der Rad. recubans, welche aber viel längere Bätter hat.” [the form of the lobule is similar to *Radula recubans*, which however has longer leaves].

Stephani (1884) identified a single specimen gathered by Pentzke in 1882 from the ‘Zamtree River’ held in the herbarium of Mitten as the type of *Radula mittenii*, in so doing effectively identified a holotype. Zamtree River is a lexigraphical error for the hand-written locality on the holotype, which is “Zaintree”. This is a corruption of ‘Daintree’, the river immediately south of Cape Tribulation in north Queensland.

In this holotype (NY00831342) are three elements: two separate pieces of material and three shoots loose within the packet. These three elements comprise three different *Radula* species.

The first element has shoot systems that are sparingly dichotomously branched, and lobules whose interior margin is not ampliate over the stem, and in these characters it does not agree with the protologue.

The second and third elements are pinnately branched, and lobules with inflated base and truncate exterior margins, so agree in some pertinent details with the protologue. However, besides the description of shoot length, the protologue is insufficiently detailed to discriminate between elements two and three. Element two has shoots approaching 50 mm long which element 3 does not.

Of the three elements only two, the first and second, are known to occur in the vicinity of the Daintree River. The third is a southern temperate species whose northern limit is somewhere in the vicinity of the Clarence River, northern New South Wales.

As there is not much basis, beyond shoot length, for selecting between elements 2 and 3 given the protologue, Article 9, Recommendation 9A.4 of the ICN could be invoked, to preserve current usage of the name *Radula mittenii*, as a synonym of *Radula buccinifera* following Yamada (1984), as the third element is this species. However, *Radula buccinifera* does not occur at the Daintree River, from where the holotype was collected, and the holotype contains other elements that do occur there, one of which agrees with the protologue, making this undesirable. Lectotypification of the name *Radula mittenii* on the second element capitalizes on an available name for a species that has not otherwise been described. Historical misinterpretation of *Radula mittenii*, particularly by Castle (1967), is immaterial as Yamada’s proposed synonymy with *Radula buccinifera* resolved this at the specimen level. This means the name *Radula mittenii* has no current usage to preserve. We therefore lectotypify *Radula mittenii* on that element agreeing with the diagnosis and originating from the type locality, being element two within NY00831342, which is separated as a subpacket.

Of the other elements, the first corresponds to *Radula notabilis* M.A.M.Renner, described below and the third, comprising three loose shoots within the packet corresponds to *Radula buccinifera*.

The origin of these three loose *Radula buccinifera* shoots is unclear – based on current knowledge of *Radula buccinifera*’s distribution it cannot have come from the Daintree River region. The most likely explanation is that the type in Mitten’s herbarium comprises an aggregate of material from two geographical locations. It is not known how this mixing may have occurred, however there is in MEL a collection, also attributed to Pentzke in 1882 from the Daintree River, and this is a large mat of pure *Radula buccinifera*. We can rule no explanation for the attribution of *Radula buccinifera* to the tropics out at this stage. Mitten’s specimen was communicated from the Phytologic Museum of Melbourne by F. von Mueller, and it is possible the contamination, and confusion, occurred at the time duplicates were made. A duplicate of Pentzke’s collection ex herb. Mitten in herb. Stephani (G00067469!) also contains a shoot of *Radula buccinifera*, including branches, as does another duplicate ex herb Steph in BM (BM000969294!), these specimens can therefore be regarded as duplicates of the type specimen but are not isolectotypes because they do not contain the element upon which the name is lectotypified.

#### Specimens examined.

Australia, Queensland: Cook District, Leo Creek, upper Nesbit River, 13°33'S, 143°28'E, 420 m, 20 Aug 1948, *L.J. Brass 19954*, MEL1037795, as *Radula acutiloba*; Cook District, McIlwraith Range, N of Lankelly Creek at point c. 20 km from Coen, 13°53'S, 143°15'E, 5 Aug 1978, *G. Butler 591*, CANB7806509; Cook District, Daintree River National Park, Mossman Gorge, Rex Creek, 16°28'S, 145°19'E, 15 Jul 1994, *E.A. Brown 94/482 et al.*, NSW297090; Cook, Daintree National Park, Mossman Gorge, Rex Creek, upstream from swingbridge, 16°28'13"S, 145°19'42"E, 105 m, 24 Mar 2012, *M.A.M. Renner 6282, V.C. Linis & E.A. Brown*, NSW896665; Cook District, on trees, near Fallons, Cairns, Oct 1890, *C.J. Wild*, BRI-AQ722874 as *Radula javanica*; Cook District, Smithfield, 2 Jul 1890, (?)*C.J. Wild*, ex herb. C.J. Wild, BRI-AQ722872 as *Radula javanica*; Cook, Wooroonooran National Park, Bellenden Ker Range, North Babinda Creek, Goldfields track, on tree trunk 10 cm dbh, 65 m, 17°20'8"S, 145°51'59"E, 3 Apr 2012, *M.A.M. Renner 6486, V.C. Linis, E.A. Brown*, NSW897201; 6 km W of Babinda, The Boulders, 17°21'S, 145°53'E, 80 m, 3 Dec 1990, *J.A. Curnow 3737*, NSW389069 as *Radula buccinifera*, BRI; Mt. Bartle Frere, 14 Jul 2005, *M.A.M. Renner 2020 & E.A. Brown*, NSW867312; ibid, *M.A.M. Renner 2021 & E.A. Brown*, NSW897314; North Kennedy, Tully Gorge State Forest, tributary of the Tully River, 17°46'S, 145°35'E, 220 m, 1 Aug 1995, *E.A. Brown 95/129 et al.*, NSW390388; North Kennedy, Tully Gorge, 17°46'S, 145°34'E, 2 Jul 2005, *E.A.Brown & M.A.M. Renner 1822*, NSW869299; Byfield, N of Emu Park, 7 Apr 1949, *O. Selling* S-B178020; ibid, 1 Jun 1949, *O. Selling*, S-B89670 as *Radula buccinifera* det M.-L. So; Cook, Wooroonooran National Park, Bellenden Ker Range, North Babinda Creek, Goldfields track, 17°20'08"S, 145°51'59"E, 65 m, 03 Apr 2012, *M.A.M. Renner 6489, V.C. Linis & E.A. Brown*, NSW897206; Cook, Daintree National Park, Mossman Gorge, Rex Creek, 16°28'11"S, 145°19'37"E, 105 m, 24 Mar 2012, *M.A.M. Renner 6288, V.C. Linis & E.A. Brown*, NSW896672; Cook, Daintree National Park, Mossman Gorge, Rex Creek, 16°28'11"S, 145°19'37"E, 105 m, 24 Mar 2012, *M.A.M. Renner 6296, V.C. Linis & E.A. Brown*, NSW896685; Cook, Wooroonooran National Park, Babinda Stream, Goldfields track, 17°19'54"S, 145°51'52"E, 75 m, 03 Apr 2012, *M.A.M. Renner 6497, V.C. Linis & E.A. Brown*, NSW909664; 18 km SSW of Cardwell, Broadwater Forest Park, 18°25'S, 145°56'E, 60 m, 30 Nov 1990, *J.A. Curnow 3608*, NSW389070 as *Radula buccinifera* (dups BRI-AQ807558, CANB9408694); ibid, *J.A. Curnow 3610*, NSW389071 as *Radula buccinifera*; 18 km SSW of Cardwell, Broadwater Forest Park, 18°25'S, 145°56'E, 65 m, 30 Nov 1990, *H. Streimann 45379*, CANB90133854; Kennedy South, Eungella National Park, Finch Hatton Gorge, 21°04'S, 148°38'E, 225 m, 22 Jun 2005, *M.A.M. Renner 1534 & E.A. Brown* NSW866081.

### Radula notabilis

M.A.M.Renner sp. nov.

http://species-id.net/wiki/Radula_notabilis

Figs 22 –24

#### Type.

Australia: Queensland: Cook, Wooroonooran National Park, tributary of Babinda Stream 30 m above junction with Babinda Stream, forming conspicuous patches of pendant, procumbent, brown-green shoots on trunk of *Ficus* 26 cm dbh rooted in streambed, 85 m, 17°19'59"S, 145°51'40"E, 3 Apr 2012, *M.A.M. Renner 6506, V.C. Linis, & E.A. Brown*. (holotype: NSW909501; isotypes: BRI, CANB, F).

#### Diagnosis.

*Radula notabilis* can be distinguished from all members of the *Radula buccinifera* and *Radula ventricosa* species complexes by its habit, forming loosely interwoven mats of irregularly and infrequently pseudodichotomously branched shoots on tree trunks, branches and twigs, by its stem anatomy, with free external wall heavily and continuously thickened and brown pigmented, internal tangential radial wall irregularly continuously thickened by fusion of nodular trigones, tan-pigmented, medulla walls unthickened but with large nodular trigones at cell angles, unpigmented, by its trapeziform leaf lobules with exterior and interior margins parallel, and by its leaf-lobe margins crenulated due to medial thickening of external cell wall. The female bracts are relatively small in stature, asymmetrical, tightly imbricate, oblong-obovate, with the larger lobe 665–720 μm long by 440–475 μm wide, smaller lobe 620-650 μm long by 350–370 μm wide, the female bract lobules are rectangular, one half the lobe area, the apex is obtuse to broadly acute, keel arched, and the bract lobe and lobule margins are crenulate. The perianths are conical, flared at mouth, and have repand labia.

#### Description.

[From NSW909500, NSW909501 and BRI-AQ722865] Forming loosely interwoven mats on tree trunks, branches and twigs, either tightly adherent on or hanging from substrate. Live plants nitid brown-green, fading to brown in herbarium. Shoot systems irregularly and often infrequently branched, female plants predominantly pseudodichotomous due to production of pairs of subfloral innovations below gynoecia. Shoot systems monomorphic, 1.4–2.0 mm wide and up to 40 mm long, branches initially smaller in stature than parent shoot but attaining similar stature by fourth of fifth pair of leaves. Older shoot sectors retaining leaf-lobes, though older leaf lobes may partially fragment on some shoots. Stems 135–160 µm diameter, with cortical cells in a single tier of 14–26 rows; outer half brown-pigmented, inner half tan-pigmented; external free cortical cell wall heavily and continuously thickened, radial longitudinal cortical walls thin or slightly thickened, inner tangential walls heavily and more or less continuously thickened by fusion coarse nodular trigones; medullar cells in 15–32 rows, with coarse nodular trigones, lacking thickenings between trigones, occasionally with heavily and continuous thickenings, all unpigmented or faintly yellow pigmented. Cortical cells on dorsal stem surface arranged in straight longitudinal rows on young and mature shoot sectors. Leaf insertion not reaching dorsal stem mid-line, leaving one or two dorsal cortical cell rows leaf-free; leaf insertion not attaining the ventral stem mid-line, leaving two ventral cortical cell rows leaf-free. Leaf lobes rotund-elliptic to elliptic-oblong, 950–1145 µm long by 610–820 μm wide, contiguous to imbricate, not falcate, acroscopic base not sharply deflexed away from stem, plane, not interlocking over the dorsal stem surface, stem visible between leaf lobes in dorsal view; lobe margins irregular and crenulate, the interior lobe margin sometimes minutely auriculate, not or only just reaching the opposite stem margin, antical margin shallowly curved, becoming substraight in larger leaf lobes, sharply curved through nearly 90° in exterior quarter, exterior margin shallowly curved or straight, sharply curved through nearly 90° in postical quarter, postical margin straight; angle between postical lobe margin and keel c. 135°. Lobules rhombiform when small, transitional as stature increases to trapeziform with exterior and interior margins nearly parallel, one sixth to one fifth the lobe area, 370–735 µm long by 260–480 μm wide, keel straight in rhombiform lobules, arched in trapeziform lobules, angle between keel and stem 135°, keel gradually turning through 90°, keel apex and postical lobe margin flush, interior lobule margin free for one quarter to one third its length, free portion weakly ampliate on rhomboid lobules to moderately ampliate on trapeziform lobules, extending at most half way across the ventral stem surface, acroscopic margin S-shaped to curved, apical portion inclined toward stem in smaller lobules, transitional to perpendicular to stem axis in larger lobules, apex acute in rhomboid lobules transitional to obtuse in trapeziform lobules, free exterior margin straight to shallowly curved, occasionally with a small knee above the lobe-lobule junction, margins plane, crenulate; lobe-lobule junction antical to the acroscopic end of stem insertion, lobule attached to stem along 0.66–0.75 of the interior margin, stem insertion more or less linear, gently curved at acroscopic and basiscopic ends, not revolute; a single papilla present at the lobule apex and another two papilla situated on the interior lobule margin above the stem insertion. Leaf lobe cells rounded-oblong, not arranged in rows, unequally sized, 16–26 µm long by 11–19 μm wide, thin walled with triangular trigones, medial wall thickenings absent; cells of lobe margin smaller than those of leaf middle, quadrate to rectangular, 9–15 µm long and wide, interior cell walls evenly and continuously thickened, exterior cell wall thickened differentially at midwall, causing exterior margin to be crenulated, cell lumen not bulging medially; leaf lobe cell surface unornamented, smooth. Oil-bodies not known. Asexual reproduction possibly by caducous leaf lobes but sporadic, older shoot sectors usually retaining most or all of their leaf-lobes, with fragmenting leaf-lobes tearing into several pieces, fragmentation scars jagged, irregular, typically leaving part of basiscopic leaf margin attached beyond keel, shoot primordia forming as irregular buds on leaf lobe after leaf fragmentation. Dioicous. Androecia on indeterminate branches that continue vegetative, androecial bracts in 4–8 pairs, lobules epistatic, keel deeply curved, bucket-like, free apical portion triangular, apex obtuse, moderately deflexed, lobes rounded, not caducous, antheridia not seen. Gynoecia terminal on leading shoots and branches, subtended by one or two full sized subfloral innovations that are again fertile, where a single subfloral innovation is present, a ‘resting’ shoot primordium occurs in place of the second subfloral innovation; archegonia 140–170 µm tall, up to 16–18 per gynoecium on a small raised disc of tissue, encompassed by the protoperianth, archegonia neck eight cell columns; female bracts in one pair, asymmetrical, tightly imbricate, oblong-obovate, larger lobe 665–720 μm long by 440–475 μm wide, smaller lobe 620–650 μm long by 350–370 μm wide, lobules rectangular, one half the lobe area, apex obtuse to broadly acute, keel arched, margins crenulate, insertion interlocking dorsally but not ventrally, insertion equitant. Perianths 2800–3800 µm long, conical and flared at mouth, mouth irregularly repand 880–950 µm wide. Perianth walls unistratose above, with bistratose bands extending up to half way up perianth, increasing in width toward base, becoming confluent, basal perianth walls progressively increasing in thickness, 2–3 stratose. Long stem perigynium present, 5-6 stratose, external cell wall thickened and brown-pigmented, internal walls unthickened and unpigmented. Calyptral perigynium present, base of calyptra bistratose at base, unistratose above, unfertilised archegonia elevated on surface of calyptra.

**Figure 22.**
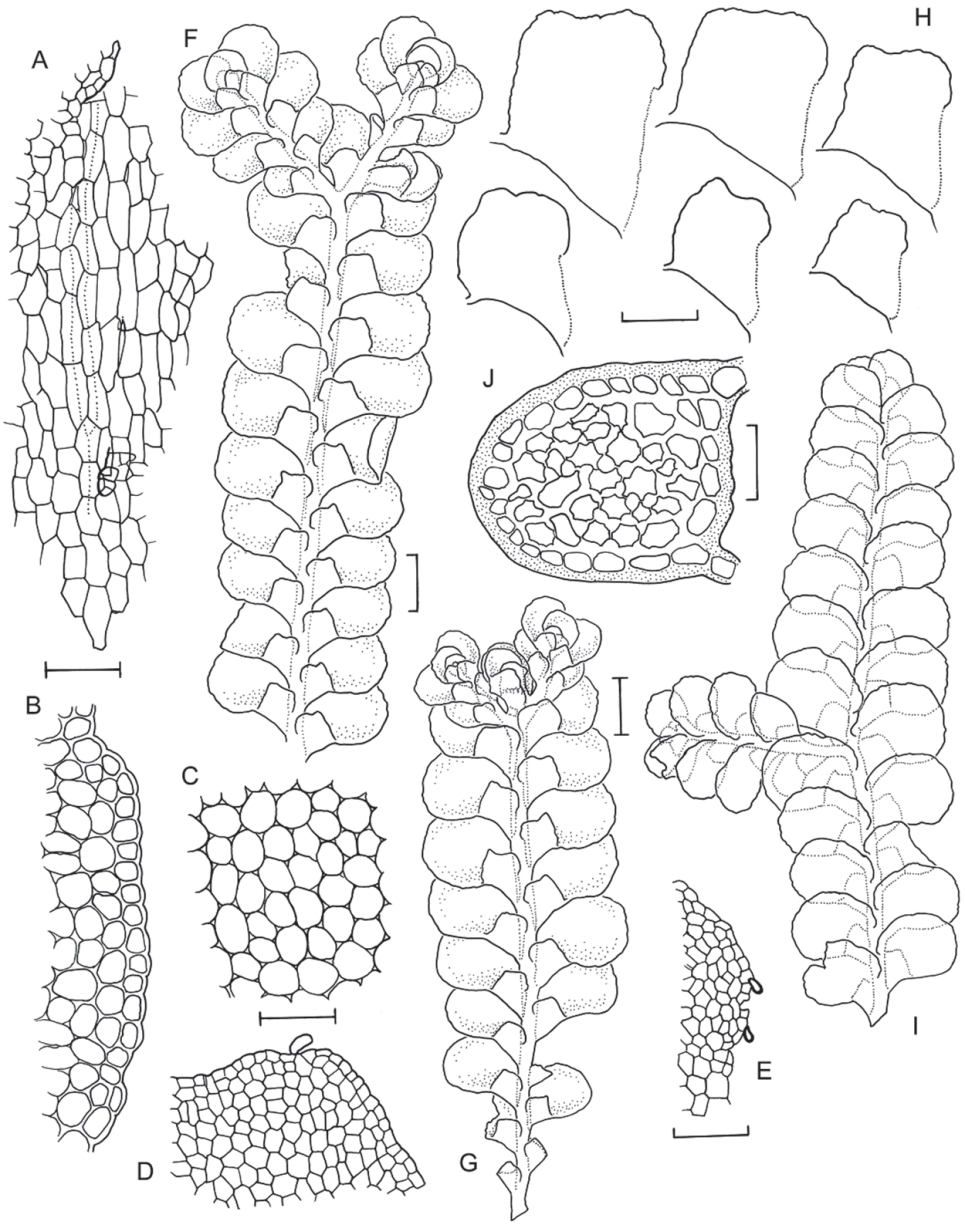
*Radula notabilis* Line drawings 1. **A** Dorsal stem surface showing leaf insertion not attaining dorsal stem mid-line leaving two cell rows leaf-free **B** Cellular detail of leaf-lobe margin **C** Cellular detail of medial leaf-lobe cells **D** Detail of lobule apex **E** Detail of lobule interior free margin **F** Ventral view of shoot, note pseudodichotomous branch **G** Ventral view of gynoecium bearing shoot **H** Five lobules showing variation in size and shape **I** Dorsal view of shoot. Scale bars: **B–C, J**: 40 µm. **A, D–E**: 60 µm, **H** 240 µm. **F, G, I**: 600 µm. **A–F, H–I** from BRI-AQ722865. **G** from NSW909500.

**Figure 23.**
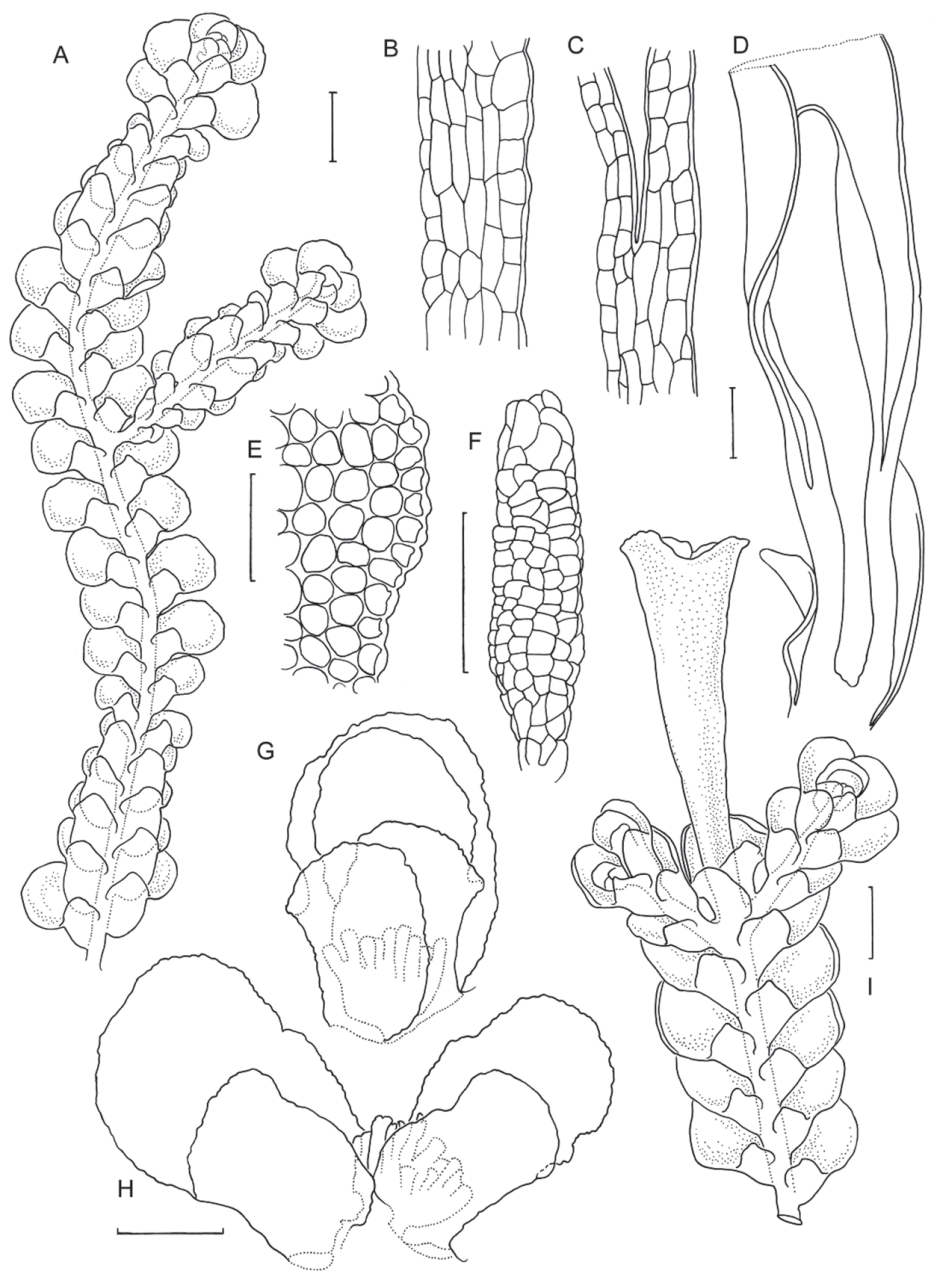
*Radula notabilis* line drawings 2. **A** Ventral view of male shoot **B** Cellular detail of stem perigynium wall **C** Cellular detail of junction between stem perigynium, perianth wall, (at right) and calyptral perigynium (at left) **D** Longitudinal section of perianth **E** Cellular detail of perianth mouth **F** Archegonium **G** Female bracts in situ **H** Female bracts flattened **I** Perianth bearing shoot **J** Transverse section of stem from primary shoot. Scale bars: **A, I**: 600 µm. **B, C, F**: 60 µm. **E, J**: 40 µm. **D, G, H**: 240 µm. All from NSW909500.

**Figure 24.**
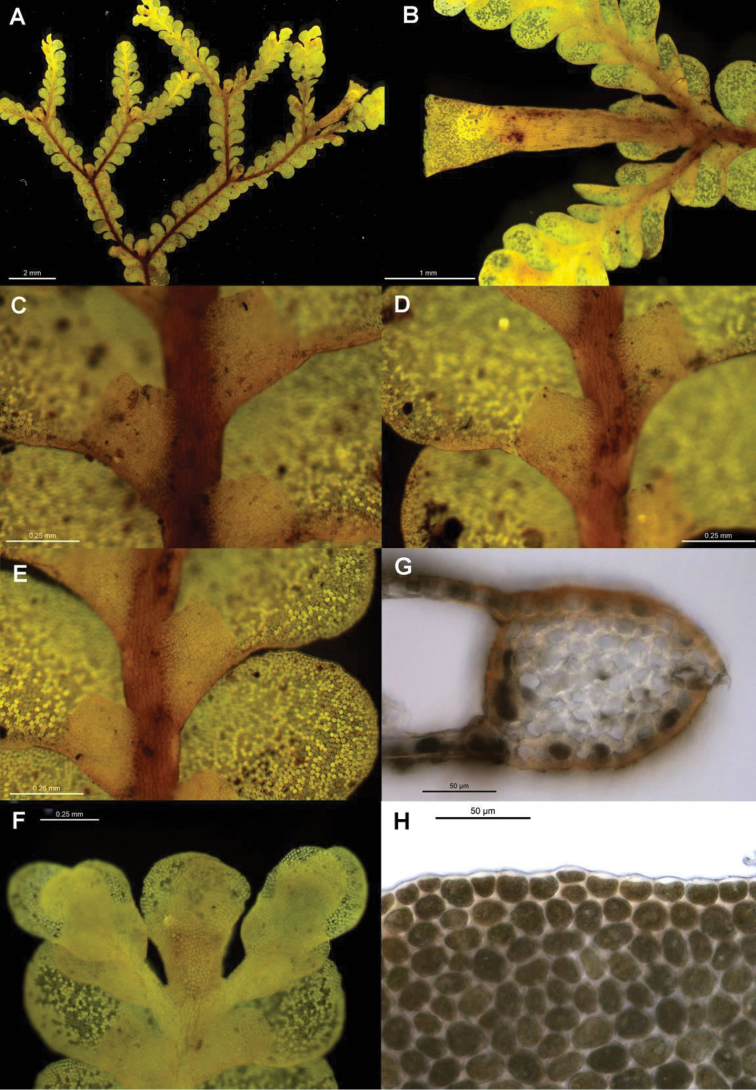
*Radula notabilis* pictures. **A** Ventral view of shoot **B** Mature perianth **C–E** Ventral view of lobules on primary shoots **F** Gynoecium **G** Transverse sections of stems from primary shoot **H** Leaf-lobe marginal cells. All from NSW896664.

#### Etymology.

From Latin *notabilis*: notable, for trumpet shaped perianths with a flared repand mouth that are distinctive among both the *Radula buccinifera* species complex, and Australasian *Radula*.

#### Distribution and ecology.

*Radula notabilis* is endemic to the Wet Tropics of north-eastern Queensland, where it is a common epiphyte in riparian rainforest in the tropical lowlands, from the edge of the coastal plain to approximately 300 m asl. *Radula notabilis* is rarely found far from running freshwater, and is usually encountered on tree trunks and branches over or adjacent to watercourses where itoften forms closely adherent mats or pendant-procumbent wefts on bark, and does not often inhabit dense multi-species epiphytic turfs.

#### Variation.

Beyond variation in shoot stature and associated size related changes in lobule shape, *Radula notabilis* is morphologically consistent across individuals.

#### Recognition.

*Radula notabilis* can be recognized by the combination of its infrequently branched monomorphic shoot systems, its slightly nitid appearance when fresh, the stem being visible between the leaves in dorsal view, the leaves being held in plane with the stem rather than being obliquely patent or dorsally assurgent, the presence of a dorsal leaf-free strip one or two cells wide; the trapeziform lobules with an obtuse apex; the perianths having a long tubular stem perigynium and walls that flare abruptly to the mouth, whose labia are undulate-repand and often partially inrolled.

*Radula buccinifera* is the only named species to have been confused with *Radula notabilis*. The two species are similar in size and colour, the presence of a dorsal leaf-free strip, gross lobule and perianth morphology. However, several character differences are accessible via critical examination. Lobules are quadrate to rhombic when small and large with typically S-shaped antical margin and obtuse to acute apex with and keel curved to straight or arched in *Radula notabilis* (Fig. 22H) vs rhombiform when small, transitional to trapeziform when large, with a straight antical margin and obtuse apex and straight to arched keel in *Radula buccinifera* (Fig. 11B). The stem anatomy of *Radula notabilis* has coarse nodular trigones on cortical and medulla cell walls vs small triangular trigones in cortical and medulla walls in *Radula buccinifera*. The perianth mouth is repand, inrolled and lobed in *Radula notabilis*. vs plane and entire in *Radula buccinifera*. In the field colour differences are sometimes apparent, with *Radula notabilis* being nitid brown-green whereas *Radula buccinifera* may be milky yellow-green through dull brown-green to mid-green, but colour is not always a reliable indicator of identity. Geography also provides a good clue to identity in that *Radula buccinifera* does not occur in the Wet Tropics Bioregion of north-east Queensland.

#### Remarks.

*Radula notabilis* is one of three elements present in the type specimen of *Radula mittenii* in herb. Mitten.

#### Specimens examined.

Australia: Queensland: Cook District, Babinda Creek, ca 1000 ft, 20 July 1983, *M.L. Hicks 11639*, BRI-AQ722865; 12 km W of Innisfail, Cooroo Lands Road, Waraker Creek, 12°32'S, 145°55'E, 80 m, 28 Jun 1984, *H. Streimann 30030*, CANB8408385; Mission Beach, 17°53'S, 146°06'E, Nov 1963, *D. McVean 26370*, CANB734330; Cook, Daintree National Park, Mossman Gorge, Rex Creek, upstream from swingbridge, 16°28'13"S, 145°19'42"E, 105 m, 24 Mar 2012, *M.A.M. Renner 6275, V.C. Linis & E.A. Brown*, NSW896419; Cook, Wooroonooran National Park, Bellenden Ker Range, North Babinda Creek, Goldfields track, 17°20'08"S, 145°51'59"E, 65 m, 03 Apr 2012, *M.A.M. Renner 6487, V.C. Linis & E.A. Brown*, NSW897204; Cook, Wooroonooran National Park, tributary of Babinda Stream 30 metres above junction with Babinda Stream, 17°19'59"S, 145°51'40"E, 85 m, 03 Apr 2011, *M.A.M. Renner 6504, V.C. Linis & E.A. Brown*, NSW909497; ibid, *M.A.M. Renner 6505, V.C. Linis & E.A. Brown*, NSW909500; ibid, *M.A.M. Renner 6506, V.C. Linis & E.A. Brown*, NSW909501; ibid, *M.A.M. Renner 6507, V.C. Linis & E.A. Brown*, NSW909502.

### Radula pugioniformis

M.A.M.Renner sp. nov.

http://species-id.net/wiki/Radula_pugioniformis

Figs 25 –27

#### Diagnosis.

*Radula pugioniformis* is outwardly similar to *Radula buccinifera*, but can distinguished by the presence of three female bracts in association with the gynoecium, the trullate to pugioniform lobules, the stem anatomy, where the cortical cell walls are heavily and continuously thickened which partially constricts the cell lumen, and the medulla cell walls are also continuously and heavily thickened, somewhat more so at cell junctions.

#### Type.

Australia: New South Wales: Central Tablelands, Small gully near Wonga Falls on Lamonds Creek, Barren Ground Nature Reserve, 34°41'S, 150°43'E, 500 m, 22 April 1992, *R.G. Coveny 16096 & P.D. Hind*, (holotype: Element 1 within NSW770504, with a portion separated in a subpacket).

#### Description.

[From NSW770504] Forming loosely interwoven mats of adherent shoots on soil and rock; live plants unknown, brown in herbarium; shoot systems monomorphic, 1.0–1.5 mm wide and up to 40 mm long, irregularly branched, though female plants predominantly pseudodichotomous due to production of pairs of subfloral innovations below gynoecia, branches initially smaller in stature than parent shoot but attaining similar stature to parent shoot by second or third pair of leaves; older shoot sectors retaining leaf-lobes. Stems 110–140 µm diameter, with cortical cells in a single tier of 19–25 rows, cortical cell walls yellow-brown pigmented, all walls heavily and continuously thickened, partially constricting individual cell lumen; medullar cells in 12–17 rows, medulla cell walls yellow-pigmented, continuously and heavily thickened, somewhat more so at cell junctions; cortical cells on dorsal stem surface arranged in straight longitudinal row on young and mature shoot sectors. Leaf insertion not reaching dorsal stem mid-line, leaving one to three dorsal cortical cell rows leaf-free; leaf insertion not attaining the ventral stem mid-line, leaving four or five ventral cortical cell row leaf-free. Leaf lobes ovate, 620–860 µm long by 500–700 μm wide, imbricate, weakly falcate or not, acroscopic base plane, not sharply deflexed away from stem, not interlocking over the dorsal stem surface, stem visible between leaf lobes in dorsal view, margins entire, the interior lobe margin weakly ampliate, not auriculate, not or only just reaching the opposite stem margin, more or less straight in larger lobes, sometimes with a single triangular tooth near the stem insertion, more often in smaller lobes, antical margin continuously curved, exterior margin curved, postical margin straight or slightly curved; angle between postical lobe margin and keel 100–135°. Lobules rhombic to trullate, one eighth to one seventh the lobe area, 240–485 µm long by 185–310 μm wide, keel slightly curved or straight, angle between keel and stem 100–135°, keel apex and postical lobe margin weakly notched, inner lobule margin free for one half to two thirds its length, free portion not ampliate, not extending across stem beyond insertion line, acroscopic margin straight to weakly curved, apex narrowly rounded to acute, free exterior margin straight, occasionally with a small knee above the lobe-lobule junction, margins plane, entire; lobe-lobule junction postical to the acroscopic end of stem insertion, attached to stem along 0.33–0.5 of the interior margin, stem insertion gently curved its entire length, not revolute; lobule apex bearing a single papilla, no other papilla, or papilla scars, observed on the interior lobule margin. Leaf lobe cells hexagonal-oblong, not arranged in rows, unequally sized, 11–21 µm long by 10–12 μm wide, walls moderately and continuously thickened. Cells of lobe margin smaller than those of leaf middle, quadrate to rectangular, 6–12 µm long by 6–10 µm wide, interior cell walls evenly and continuously thickened, exterior cell wall unthickened. Leaf lobe cell surface unornamented, smooth. Oil-bodies not known. Asexual reproduction absent. Dioicous. Androecia on short lateral branches or terminal on leading shoots, either terminating following androecia production, or continuing vegetative growth; antheridial bracts in 3–4 pairs; lobules epistatic, keel deeply curved, bucket-like, free apical portion triangular, apex acute, plane, lobes rounded, not caducous; antheridia not seen. Gynoecia terminal on leading shoots and branches, subtended by one or two full sized subfloral innovations that are again fertile, more often subtended by a single subfloral innovations on branches with a ‘resting’ shoot primordium in place of the second subfloral innovation. Archegonia 175–190 µm tall, archegonia neck five or six cell columns, 12–14 per gynoecium on a small raised disc of tissue, encompassed by the protoperianth, with several large single celled or stalked (on 2 or 3 cells) papillae scattered among gynoecia. Female bracts three, symmetrical, imbricate, obovate-falcate, lobe 1140–1255 μm long by 670–795 μm wide, lobules triangular, one half the lobe area, 585–930 μm long by 340-645 μm wide apex acute, keel arched, margins entire, insertion interlocking dorsally and ventrally, insertion equitant. Perianths and sporophytes not known.

**Figure 25.**
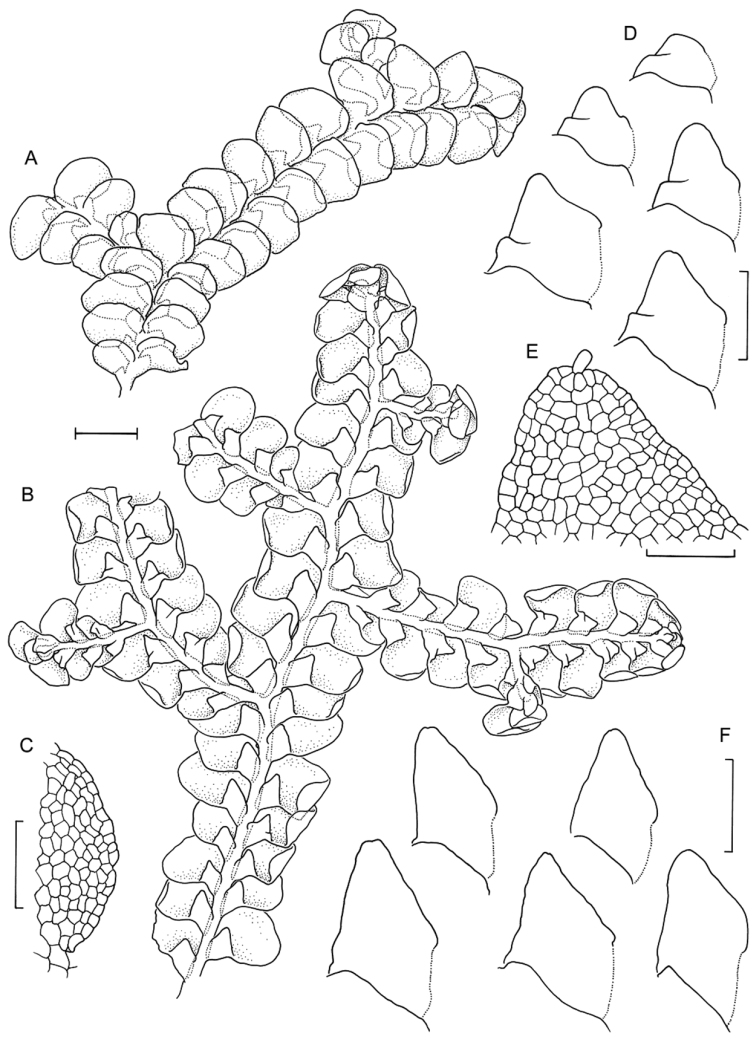
*Radula pugioniformis* line drawings 1. **A** Dorsal view of shoot **B** Ventral view of shoot **C** Detail of lobule interior free margin **D** Five lobules from secondary shoots showing variation in size and shape **E** Detail of lobule apex **F** Five lobules from primary shoots showing variation in size and shape. Scale bars: **A–B**: 600 µm. **C, E**: 60 µm. **D, F**: 240 µm. All from NSW770504.

**Figure 26.**
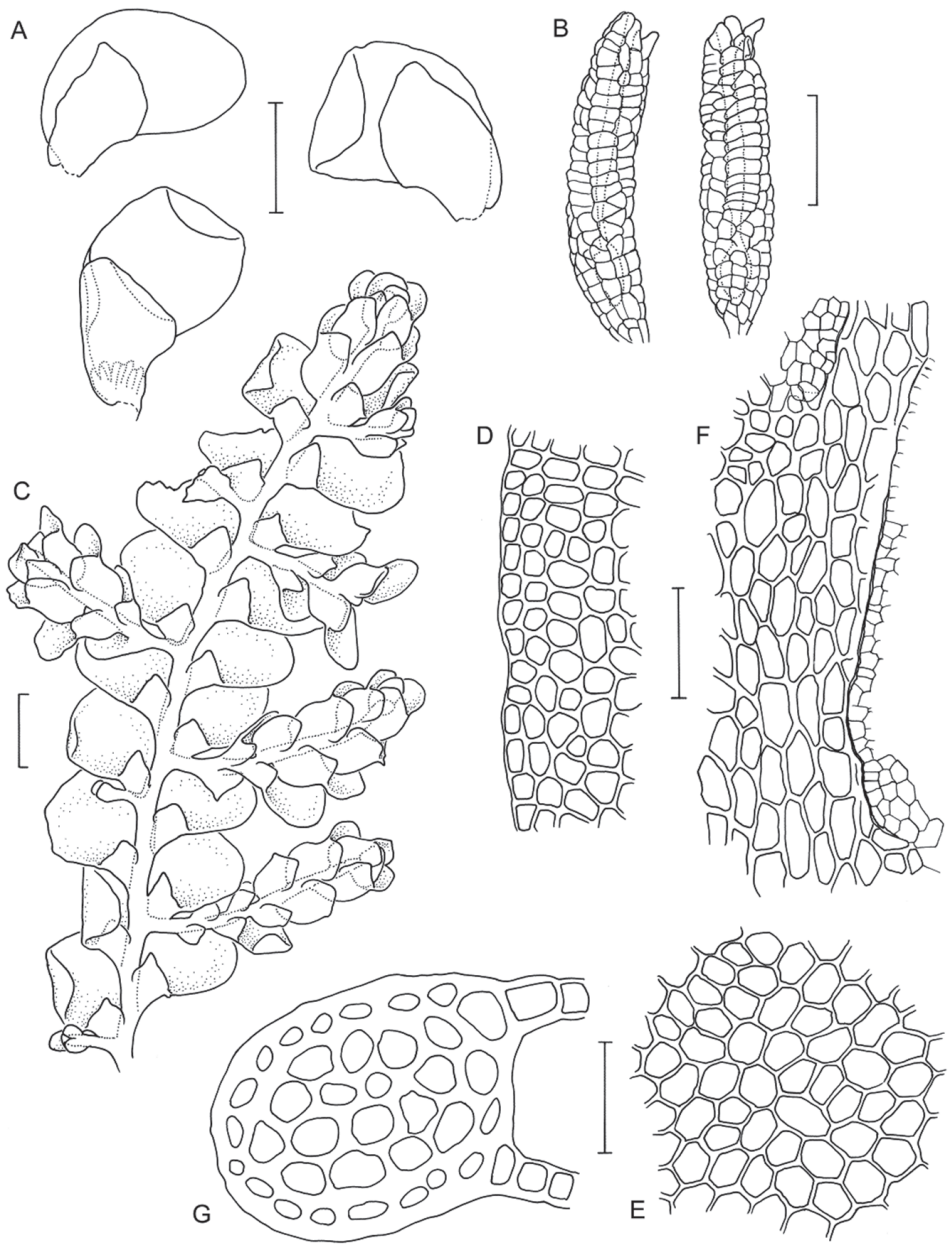
*Radula pugioniformis* line drawings 2. **A** Three female bracts, outer, middle and innermost clockwise from top left **B** Archegonia **C** Ventral view of male shoot **D** Cellular detail of leaf-lobe marginal cells **E** Cellular detail of leaf-lobe marginal cells **F** Dorsal stem surface showing leaf insertion lines not attaining the dorsal stem mid-line, leaving two dorsal leaf-free strip two cell rows wide **G** Transverse section of stem from primary shoot. Scale bars: **A, C**: 600 µm. **B, F**: 60 µm. **D, E, G**: 40 µm. All from NSW770504.

**Figure 27.**
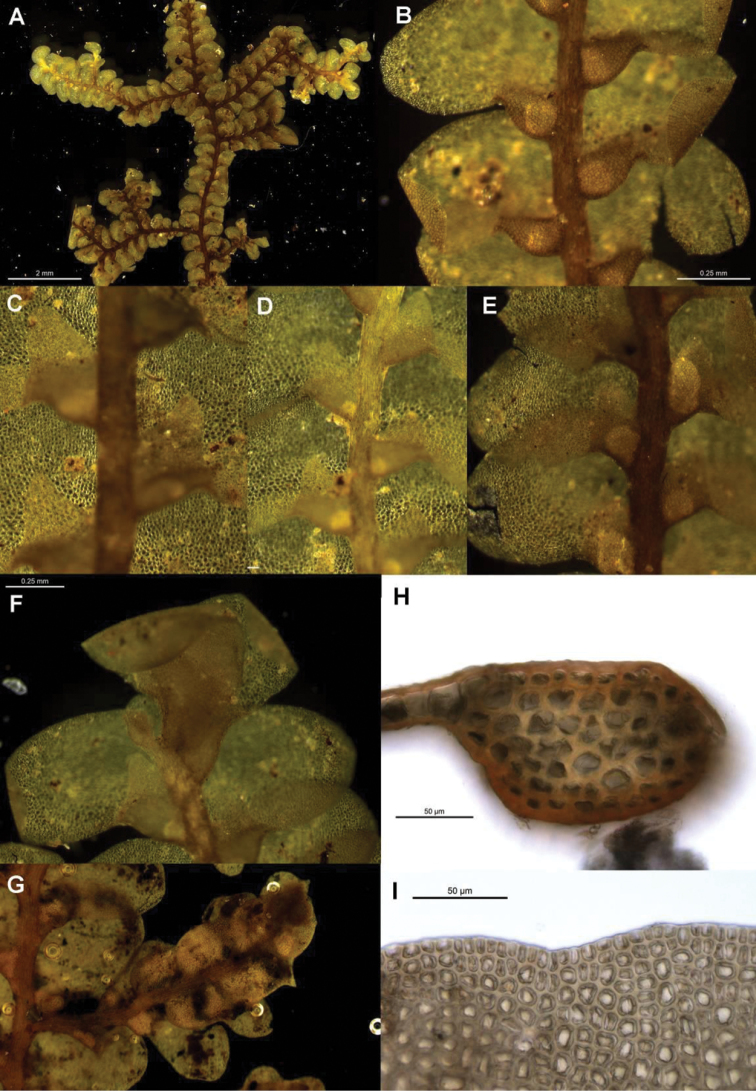
*Radula pugioniformis* pictures. **A** Ventral view of shoot. B-E: Ventral view of lobules on primary shoots **F** Gynoecium **G** Androecium **H** Transverse sections of stems from primary shoot **I** Leaf-lobe marginal cells. All from NSW770504.

#### Etymology.

From Latin *Pugioniformis*, with the form of a dagger, in reference to the resemblance of the lobules to broadly triangular iron daggers adopted by several ancient cultures, including in the Roman Empire as the ‘Pugio’.

#### Distribution and ecology.

*Radula pugioniformis* is known from a range of localities in the Central and Southern Tablelands of New South Wales. The two specimens with details on substrate indicate *Radula pugioniformis* grows on wet mud and rocks in and around streams or other watersources, in gullies or on steep slopes. *Radula pugioniformis* has been collected growing with *Radula buccinifera*, *Acrophyllum dentatum*, *Thuidium furfurosum*, and *Bryum* sp. on soil, and with *Lejeunea* sp. on rock.

#### Variation.

Within individuals, shoot stature varies, and this is correlated with changes in lobule shape, which tend to be shorter on smaller shoots.

#### Recognition.

*Radula pugioniformis* is outwardly similar to *Radula buccinifera*, and inhabits a subset of microhabitats occupied by that species. Differentiating these two species is best achieved on the basis of hydrated, and preferably slide mounted material. The most accessible character by which *Radula pugioniformis* and *Radula buccinifera* differ is in shape of the leaf lobules. In *Radula pugioniformis* the lobules are conspicuously trullate, the antical margin is straight to curved, and slopes steeply toward the stem at 45–70°, such that the lobule apex lies well above the antical end of the stem insertion. In *Radula buccinifera* the lobules are quadrate to rhombic, the lobule apex lies at variably between the same level as, or slightly above, the uppermost point of the ampliate portion of the lobule margin. Between these two points the lobule margin varies from straight to S-shaped in situ (straight when flattened), but when S-Shaped there is a pronounced medial curve. The slope of the antical margin varies between sloping downward toward the stem at up to 45° and remaining level.

Other diagnostic differences between *Radula pugioniformis* and *Radula buccinifera* can be found in the stem anatomy. In *Radula pugioniformis* the cortical cell walls are heavily and continuously thickened, which partially constricts the cell lumen, and the medulla cell walls are continuously and heavily thickened, somewhat more so at cell junctions. In *Radula buccinifera* the free external cortical cell wall is differentially thickened, but all other cortical and medulla cell walls bear triangular trigones at most, and are otherwise unthickened.

*Radula pugioniformis* is similar to *Radula iwatsukiana* K.Yamada from New Caledonia, and may be related to this species. However, the type of *Radula iwatsukiana* was not available for study due to CITES restrictions, and interpretation of morphology via descriptions and illustrations is fallible. The size of female bracts in both absolute terms and relative to vegetative leaves differs in *Radula pugioniformis* from that described and illustrated by Yamada (1985), the illustrations also suggest a sharp distinction between marginal and medial cells in *Radula iwatsukiana* that does not occur in *Radula pugioniformis*. If Yamada’s illustration is representative of *Radula iwatsukiana*, there are also differences in the relative sizes of cortical and medulla cells in the stem; in *Radula iwatsukiana* these are approximately the same area in transverse section, while in *Radula pugioniformis* the cortical cells are half to one quarter the area of the medulla cells. They are also far more numerous.

#### Remarks.

Female bract number suggests this species belongs to subg. *Odontoradula*, not subg. *Metaradula*. Although apparently not closely related to species of the *Radula buccinifera* complex, *Radula pugioniformis* is included here because it has been misidentified as *Radula buccinifera*.

#### Specimens examined.

New South Wales, Southern Tablelands, Tumbarumba District, November 1900, *W. Forsyth*, NSW764133; ibid, H228, NSW; Southern Tablelands, slopes of Mt Buddawang, near Mongarlowe, 28 October 1965, *L.G. Adams 1427*, NSW764186.

### Radula strangulata

Hook.f. et Taylor London Journal of Botany 5: 377. 1846.

http://species-id.net/wiki/Radula_strangulata

Figs 28 –32

Radula strangulata Type: New Zealand: *J.D. Hooker*, 1840, ex herb Taylor (holotype: FH00258850! isotypes: ex herb. Lehmann S-B43503! S-B43504! S-B43505!).
Radula levieri Steph. Species Hepaticarum. 4: 227. 1910.
Radula strangulata Type: New Zealand: damp forest, Westland, Kelly’s Range, Perry Ridge, 1903, leg. *T.W.N. Beckett 298* ex Hb. Levier No. 4612 in Hb. Steph. (lectotype (designated by Castle 1967, p. 74): G00067466!)
Radula silvosa E.A.Hodgs. et K.W.Allison in Hodgson, Transactions and Proceedings of the Royal Society of New Zealand 74: 286. 1944.
Radula strangulata Type: New Zealand: Te Tiki Station, Wairoa ca. 1500 ft, No.24766 P.R.B. Herb. (holotype: MPN!)
Radula parviretis E.A.Hodgs. Transactions of the Royal Society of New Zealand. Botany 3: 87. 1965.
Radula strangulata Type: New Zealand: Rakiura [Stewart Island]: landing for Tin Range, Port Pegasus, 10 Jan 1949, *W. Martin*, herb. Hodgson 9746. (holotype: MPN!)

#### Description.

[From CHR579214] Forming interwoven mats of shoots, glaucous yellow-green to brown-green in life, brown in herbarium; shoot systems regularly pinnately branched in male plants and sterile female plants, but pseudodichotomous in fertile female plants due to production of pairs of subfloral innovations below gynoecia, monomorphic, 1.0–2.0 mm wide and up to 40 mm long, branches initially smaller in stature than parent shoot and attaining similar stature by third to fifth pair of leaves; older shoot sectors retaining leaf-lobes. Stems 115–175 µm diameter, with cortical cells in a single tier of 16–25 rows, cell walls yellow-brown to brown pigmented, external free cortical cell wall heavily and continuously thickened, radial longitudinal walls thin, inner tangential walls thin or continuously thickened; medulla cells in 18–35 rows, cell walls yellow-brown pigmented, sometimes deepening to brown pigmented on old shoot sectors, cell walls with small triangular trigones, walls between trigones lacking thickenings. Cortical cells on dorsal stem surface arranged in straight longitudinal rows on young and mature shoot sectors. Leaf insertion not reaching dorsal stem mid-line, leaving four or five dorsal cortical cell rows leaf-free; leaf insertion not attaining the ventral stem mid-line, leaving two or three ventral cortical cell rows leaf-free. Leaf lobes elliptic-ovate, 550–1050 µm long by 400–830 μm wide, remote to contiguous, not falcate, acroscopic base not sharply deflexed away from stem, flat, lying in plane with stem, not interlocking over the dorsal stem surface, stem visible between leaf lobes in dorsal view; margins irregularly but minutely repand, minutely crenulate, the interior lobe margin not or at most shallowly ampliate, hardly covering the dorsal stem surface and not reaching the opposite stem margin, antical margin curved, exterior margin curved through nearly 100°, postical margin straight; angle between postical lobe margin and keel c. 135°. Lobules quadrate when small to oblong, one twelfth to one sixth the lobe area, 270–490 µm long by 140–270 μm wide; keel straight to shallowly arched, angle between keel and stem 135–150°, keel turning through 90° at keel-lobe junction, keel apex and postical lobe margin flush; interior lobule margin free for one fifth to one quarter its length, free portion not ampliate in small stature lobules to moderately ampliate on large lobules, extending at most half way across the ventral stem surface; acroscopic margin S-shaped, apical portion perpendicular to stem; apex obtuse to apiculate; free exterior margin straight, margins plane, crenulated; lobe-lobule junction well postical to the acroscopic end of stem insertion; attached to stem along 0.75 to 0.8 of the interior margin, stem insertion more or less straight, not curved at acroscopic or basiscopic ends, not revolute; lobule apex bearing a single papilla, with another two papilla situated on the interior lobule margin above the stem insertion. Leaf lobe cells rounded-oblong, not arranged in rows, unequally sized, 10–25 µm long by 9–19 μm wide, thin walled with small triangular trigones, medial wall thickenings absent; cells of lobe margin smaller than those of leaf middle, quadrate to rectangular, 9–15 µm long and wide, interior and exterior cell walls not differential thickened, cell lumen bulging medially; leaf lobe cell surface unornamented, smooth. Oil-bodies two or three per cell, ellipsoidal, filling cell lumen, light-brown, surface granular, internally homogeneous or with a hyaline droplet. Asexual reproduction usually absent, however two specimens have been observed producing bud-like shoot primordia from leaf lobe margins. Dioicous. Androecia on indeterminate branches that continue vegetative or reproductive growth, androecial bracts in 4-∞ pairs, lobules epistatic, keel deeply curved, bucket-like, free apical portion triangular, apex obtuse, moderately deflexed, lobes rounded, not caducous, antheridia not seen. Gynoecia terminal on leading shoots, subtended by two full subfloral innovations that are usually full-sized and again fertile; archegonia 130–155 µm tall, archegonia neck five or six cell columns, 6–8 per gynoecium on a small disc of tissue, not encompassed by the protoperianth; female bracts in one pair, slightly asymmetrical, tightly imbricate, elliptic-ovate, weakly falcate, lobe 840–1015 μm long by 460–545 μm wide, margins entire; lobules rhomboid to trullate, one quarter to one half the lobe area, apex acute to acuminate, keel straight to arched, margins entire; bract insertion lines interlocking dorsally and ventrally, insertion equitant. Perianths 3200–3800 µm long and 840–980 µm at mouth, mouth entire to irregularly lobed, perianth shape variable, either broadening from mouth to widest point at approximately one third to one half length above base, where 850–950 µm wide, then tapering to base, or tapering from mouth to base; perianth walls unistratose above, with bistratose collar 3 or 4 cell tiers high above the perianth-calyptra junction; long stem perigynium present, 5-6 stratose, cell walls not thickened or pigmented, perianth-calyptra fusion elevated above female bracts on 9–15 tiers of cells; calyptral perigynium present, base of calyptra bistratose, unistratose above, unfertilised archegonia elevated on surface of calyptra.

**Figure 28.**
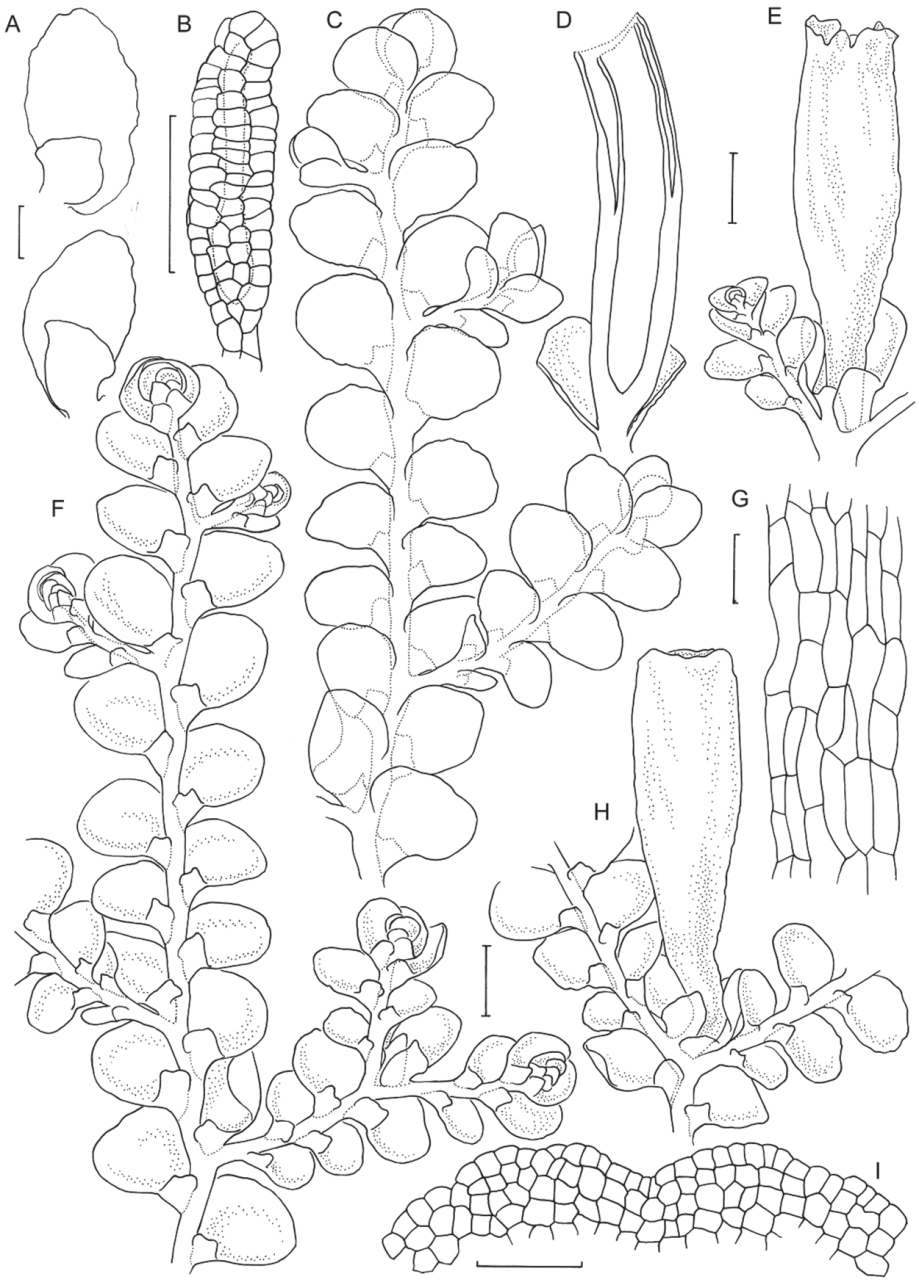
*Radula strangulata* line drawings 1. **A** Female bracts **B** Archegonium **C** Dorsal view of shoot **D** Perianth transverse section **E** Perianth **F** Ventral view of shoot **G** Cellular detail of stem perigynium wall **H** Perianth **I** Detail of perianth mouth. Scale bars: **A, D**: 240 µm. **B, G**: 60 µm, **I**: 40 µm. **C, E, F, H**: 600 µm. All from CHR579214.

**Figure 29.**
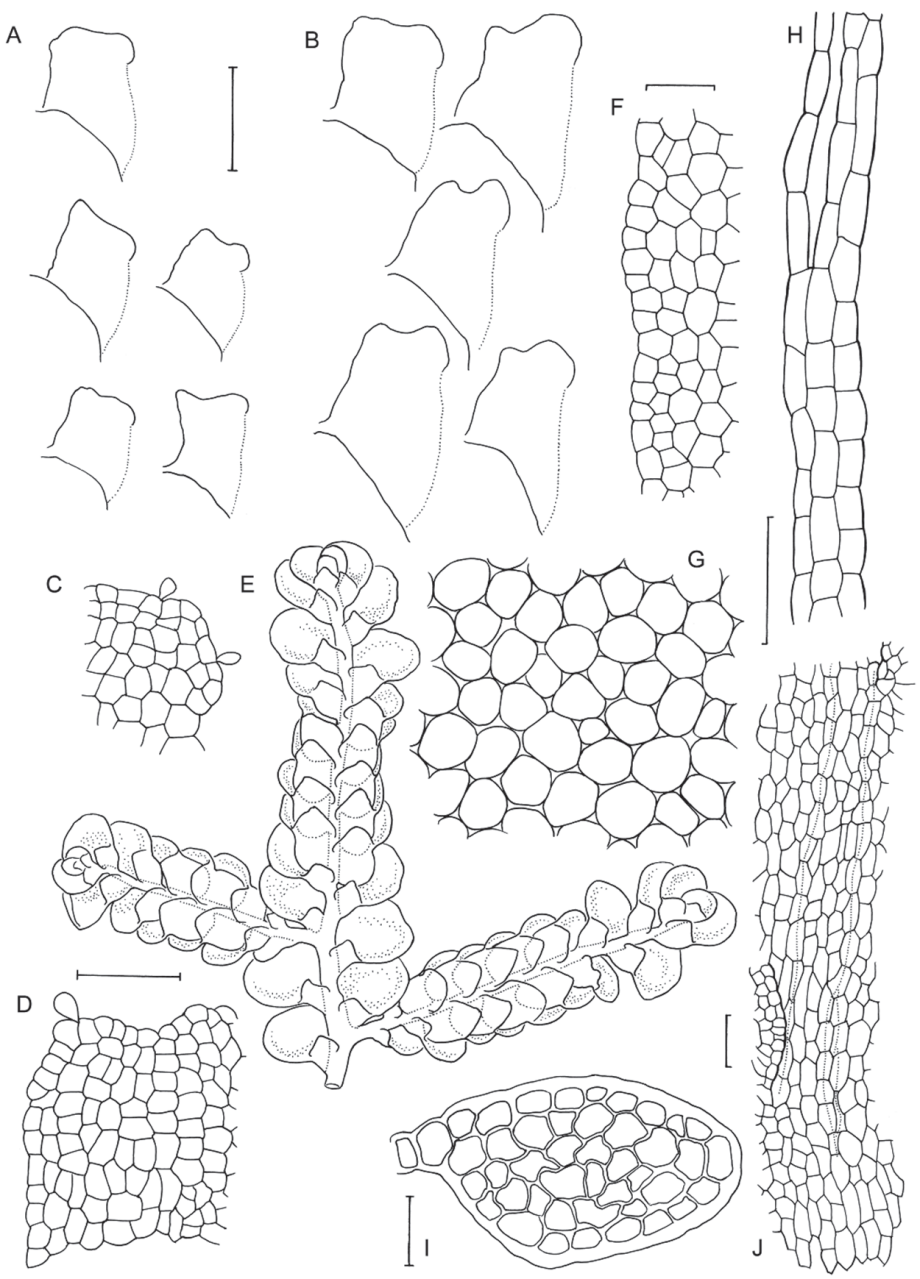
*Radula strangulata* line drawings 2. **A** Five lobules from secondary shoot, showing variation in size and shape **B** Five lobules from primary shoot showing variation in size and shape **C** Detail of interior free lobule margin **D** Detail of lobule apex **E** Ventral view of male shoot **F** Detail of leaf-lobe marginal cells **G** Detail of leaf-lobe medial cells **H** Cellular detail of junction between stem perigynium, perianth wall, (at right) and calyptral perigynium (at left) **I** Transverse section of stem from primary shoot **J** Dorsal stem surface showing leaf insertion lines not attaining the dorsal stem mid-line, leaving a dorsal leaf-free strip four or five cell rows wide. Scale bars: E: 600 µm. **A–B**: 240 µm. **C–D, J**: 60 µm. **F, G, H, I**: 40 µm. **E** from NSW896412. All others from CHR579214.

**Figure 30.**
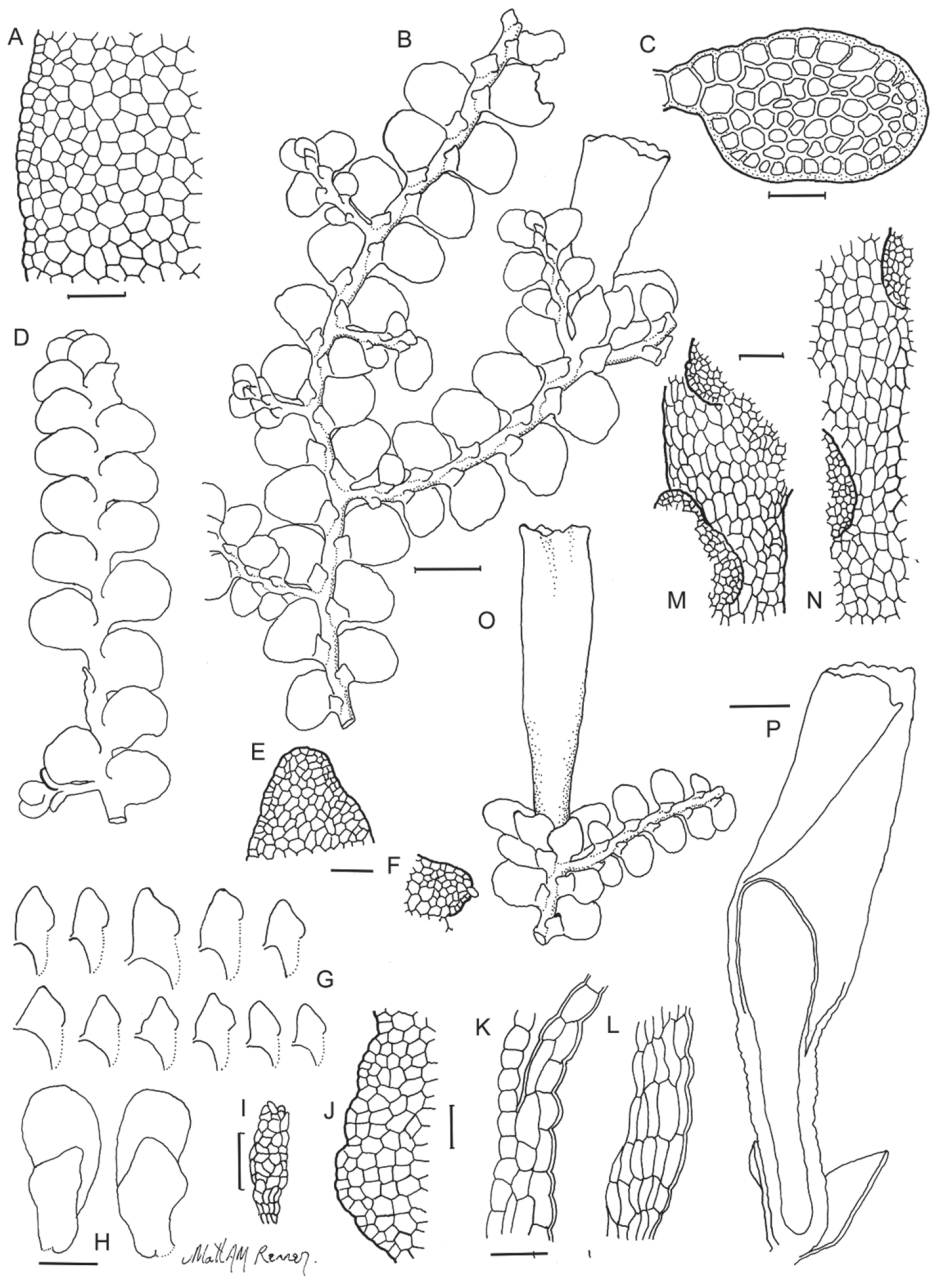
*Radula strangulata* line drawings 3. **A** Cellular detail of leaf-lobe margin **B** Ventral view of female shoot **C** Transverse section of stem from primary shoot **D** Dorsal view of shoot **E** Cellular detail of lobule apes **F** Cellular detail of lobule base **G** A rangeof lobules **H** Female bracts **I** Archegonium **J** Cellular detail of the perianth mouth **K** Cellular detail of junction between stem perigynium, perianth wall (at right), and calyptral perigynium (at left) **L** Cellular detail of junction between stem perigynium, perianth wall, (at right) and calyptral perigynium (at left) **M** Ventral stem surface **N** Dorsal stem surface showing leaf insertion lines not attaining the dorsal stem mid-line, leaving a dorsal leaf-free strip four or five cell rows wide **O** Perianth **P** Perianth transverse section. Scale bars **A, C, J–L**: 40 µm. **B, D, O**: 600 µm. **E–F, I, M, N**: 60 µm. **G–H, P**: 240 µm. All from NSW875811.

**Figure 31.**
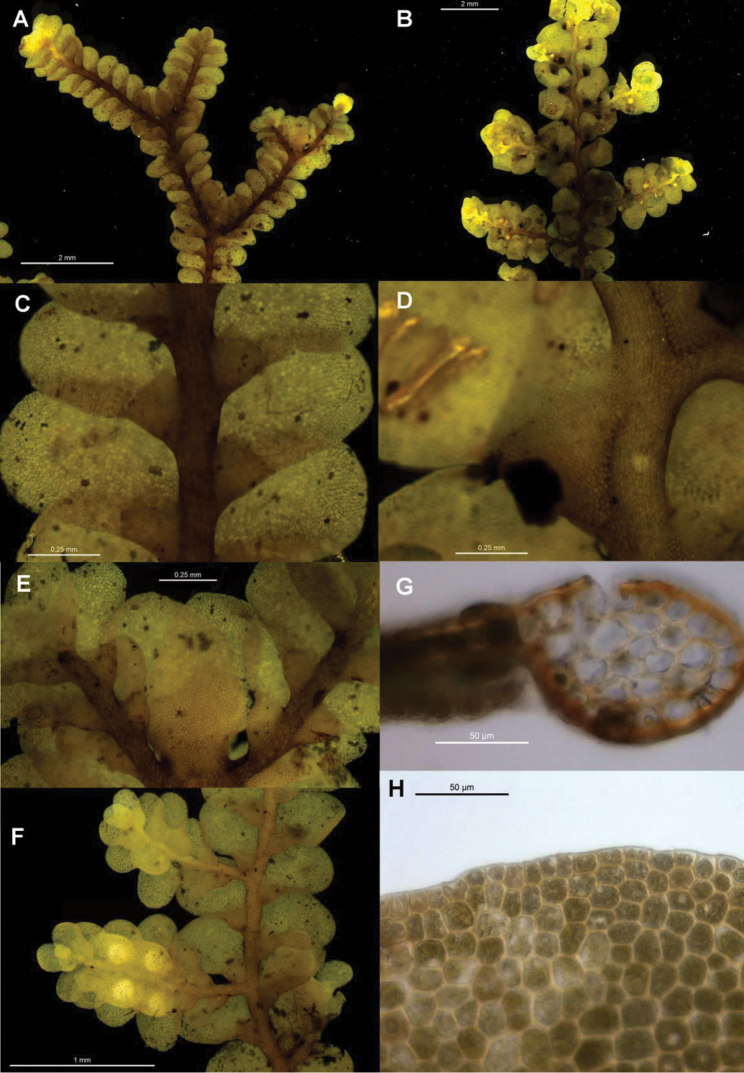
*Radula strangulata* Pictures 1. **A–B** Ventral view of shoots showing variation in size, leaf-lobe spacing and colour between individuals **C–D** Ventral view of lobules on primary shoots showing variation in size and shape between individuals **E** Gynoecium **F** Androecium **G** Transverse sections of stems from primary shoot **H** Leaf-lobe marginal cells. **B, D**: NSW909416, **F** NSW896405, others: NSW970841.

**Figure 32.**
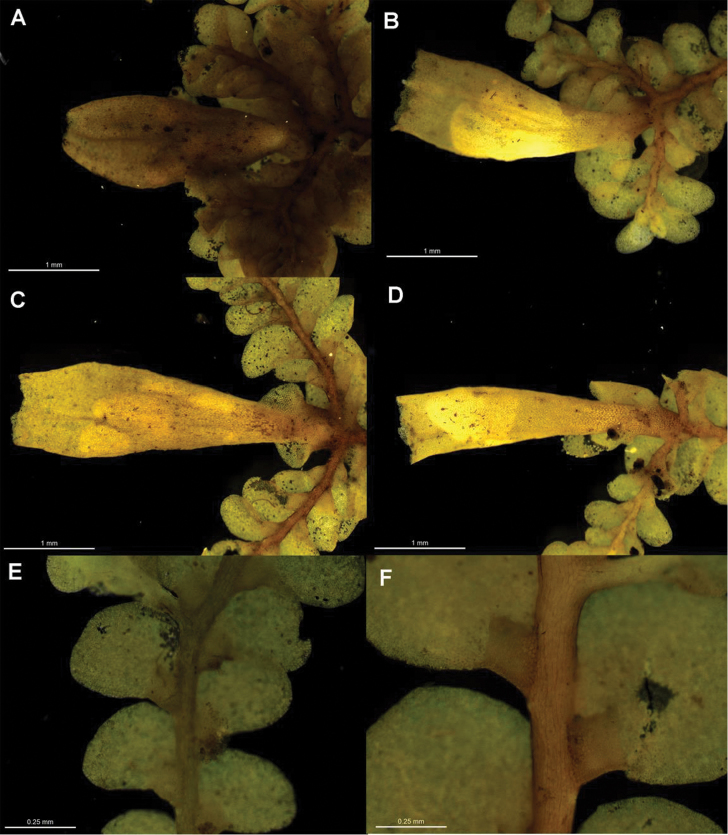
*Radula strangulata* Pictures 2. **A–D** Perianths showing variation in size and shape associated with development **A** youngest **D** oldest and mature **E–F** ventral view of lobules on primary shoots illustrating more inter-individual variation in size and shape of lobules and leaf-lobes **E** NSW896405 **F** NSW895357. Others NSW970841.

#### Etymology.

Strangulating, in reference to the entwining growth about a shoot of *Weymouthia cochlearifolia* exhibited by the type specimen.

#### Distribution and ecology.

*Radula strangulata* is widely distributed throughout the New Zealand Botanical Region, from the Kermadec Islands in the north, throughout the North, South, and Stewart Islands, south to the Auckland and Campbell Islands, and east to the Chatham Islands. *Radula strangulata* occupies an elevational range from sea level to c. 800 mencompassing lowland to montane habitats, including coastal scrub, mature and regenerating lowland podocarp-broadleaf forest and beech forest on both sides of the main axial ranges in North and South Islands. *Radula strangulata* also has a broad ecological amplitude, occupying a range of microsites from tree trunks and bases, exposed tree roots on the forest floor, to rotting logs, exposed clay on forest banks, dripping rocks adjacent to waterfalls, and on rocks within stream beds, sometimes under running water. In hyperhumid locations *Radula strangulata* may grow epiphyllously on fern fronds. This is the only *Radula* species in New Zealand commonly encountered growing aquatically, typically submerged on basaltic boulders within cool, clear, fast flowing streams. It is also the only *Radula* species encountered in suburban areas where it is opportunistic in a range of man-made habitats in suitably moist sites, for instance the surfaces of rotting wooden roof tiles.

*Radula strangulata* is the most commonly collected species of *Radula* in New Zealand, and despite this accessibility bias, is probably the most common species of *Radula* in New Zealand.

When growing on naked bark *Radula strangulata* forms tightly adherent mats, and usually grows admixed with *Radula allisonii*. *Radula strangulata* also grows in epiphytic turfs with *Radula plicata*, *Radula demissa*, *Archilejeunea olivacea*, various *Cheilolejeunea* species, *Plagiochila* spp. In terrestrial habitats and on rotting logs, *Radula strangulata* occurs with a wide variety of bryophytes, including the mosses *Pendulothecium punctatum*, *Echinodium umbrosum*, *Catharomnion ciliatum*, *Fissidens tenellus* var. *australiensis*, and the liverworts *Heteroscyphus* spp., *Chiloscyphus* spp., *Saccogynidium* and many other species. On rocks and tree roots on the forest floor with *Pendulothecium oblongifolium*, *Pendulothecium punctatum*, *Acromastigum colensoanum*, *Chiloscyphus muricatus*, *Radula marginata*.

#### Variation.

*Radula strangulata* exhibits a broad amplitude of morphological variation, as might be expected from the diverse array of habitats occupied by this species. This, in conjunction with the relatively deep phylogenetic divergences between groups of individuals suggests *Radula strangulata*, as circumscribed here, could well be a complex of weakly morphologically differentiated sister species. Variation in gametophyte morphology appears at least partly correlated with moisture regime of the occupied microsite. Plants growing in dry habitats and microsites have contiguous to imbricate leaf lobes, small rhomboid lobules, and are glaucous green. The origin of this glaucous colouration is not known, however it is not due to cell surface ornamentation. The type material of *Radula silvosa* is typical of plants growing in dry sites. Plants growing at the wet end of the moisture spectrum have remote leaf lobes, large rectangular lobules with a well developed ampliate interior margin when large, and are dark green. The type material of *Radula levieri* is typical of plants from wet sites.

**Figure 33.**
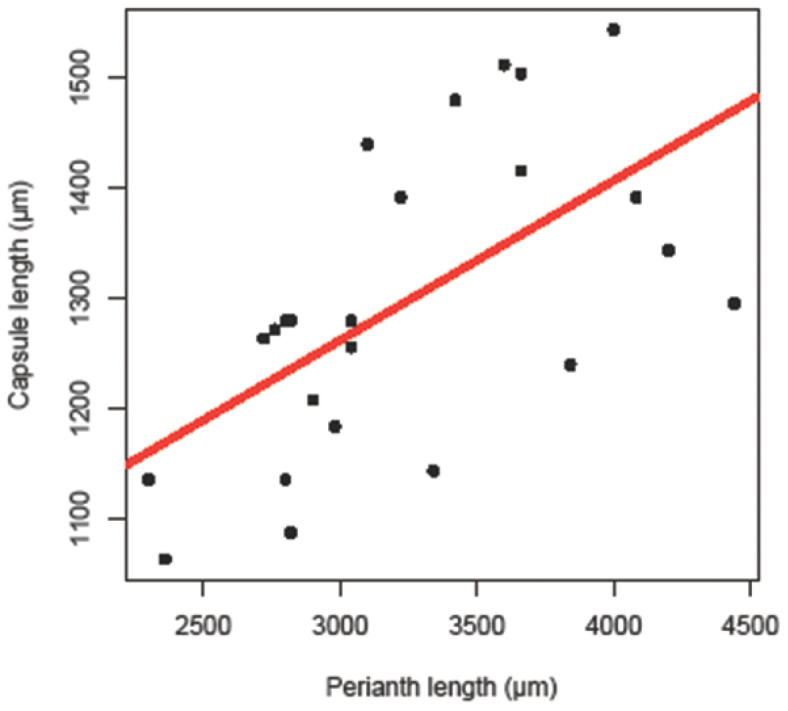
Scatterplot of perianth vs capsule valve length in three individuals of *Radula strangulata* from New Zealand. The red line shows the regression slope from the general linear model explaining the relationship between the two variables.

Part of the justification presented by Hodgson (1944) for the recognition of *Radula silvosa* was that the perianths abruptly inflated above a tubular base, as opposed to gradually narrowed from mouth to base in *Radula levieri* (a species she accepted). The former feature is apparent in juvenile perianths, but is lost as the stem perigynium subtending the perianthium grows and matures prior to eruption of the sporophyte from the calyptra. All perianths we have seen wherein the sporophyte has ruptured are gradually tapered from apex to base. In addition to developmental changes in perianth shape, perianth length at maturity varies, and this variation is positively correlated with capsule valve length (*R*^2^=0.368, *F*_(1, 22)_=12.81, *P*=0.0017) (Fig. 33).

#### Recognition.

Despite the variability exhibited by *Radula strangulata* this species is relatively easy to recognize. *Radula strangulata* inhabits forest interiors, frequently in microsites on or close to the forest floor, and often in relative shade. The other species of the *Radula buccinifera* complex with which *Radula strangulata* could be confused on morphological grounds occupy quite different microsites. *Radula australiana* inhabits subalpine and alpine habitats typically dominated by shrubland, tussockland or grassland, and almost always above tree-line. Like *Radula strangulata*, *Radula demissa* is a forest inhabitant, but is typically an epiphyte on tree trunks, branches, twigs and occasionally leaves, in reasonable light. When fresh, plants of *Radula strangulata* growing in relatively dry microsites have a distinct glaucous bloom. This is a distinctive character that, in combination with microsite can facilitate identification in the field. However, not all plants are glaucous, and microsite differences from other members of the complex are not absolute.

The most accessible morphological character by which *Radula strangulata* differs from *Radula demissa* is the orientation of the leaf lobes. In *Radula strangulata* the leaves lay in plane and alongside the stem, such that the dorsal stem surface is at least partially visible from above. In *Radula demissa* the leaves are obliquely-patent, and spread upward away from the stem and overlap one another across the dorsal stem surface, such that the dorsal stem surface is not usually visible from above.

If this character proves ambiguous, lobule shape is a source of diagnostic differences. *Radula strangulata* has lobules with a straight keel, and the carinal region is a weakly inflated mound set back from the keel. In contrast, *Radula demissa* typically has a curved keel, and the carinal region is strongly inflated along the length of the keel. *Radula strangulata* expresses a range of lobule morphologies within a single population, from small rhomboidal lobules to large rectangular lobules. In rhomboidal lobules the apex lies close to the stem, and the exterior margin is noticeably inclined toward the stem and the exterior margin is straight. In *Radula demissa*, lobule morphology is more conservative, with the smallest lobules being approximately quadrate such that the apex is closer to the keel apex, and the exterior margin is not noticeably inclined toward the stem. The exterior margin of *Radula demissa* is curved. Another useful difference is found in the junction of the lobule and the lobe. In *Radula strangulata* this junction forms a simple angle of c. 135°, and the postical lobe margin then continues perpendicular to the stem in a straight line. In *Radula demissa* this junction forms a notch, and the postical margin is weakly to strongly falcate, and curved. Diagnostic differences between *Radula strangulata* and *Radula demissa* can also be derived by counting the number of rows of dorsal cortical cells that are not crossed by the leaf insertion lines. In *Radula strangulata* 2–5 rows are leaf-free, whereas no rows are leaf free in *Radula demissa*.

*Radula strangulata* has been confused with two other *Radula*, *Radula australiana* and *Radula buccinifera*. As noted above *Radula australiana* is primarily an alpine species. Lobule shape provides the best morphological differences between *Radula strangulata* and *Radula australiana*. Whereas the lobules in *Radula strangulata* are rhomboid to longitudinally rectangular, in *Radula australiana* they are more or less quadrate. Lobules in *Radula australiana* are typically larger in comparison to the lobe size than in *Radula strangulata*, and may be up to one quarter the lobe area. The lobule keel and its junction with the leaf lobe also differentiate these two species. In *Radula strangulata* the keel is straight, and forms an angle of c. 135° at the junction with the lobe, the postical lobe margin then continues perpendicular to the stem in a straight line. In *Radula australiana* the keel is curved, and continues evenly into the rounded outline of the leaf without forming a notch or angle at the junction. Diagnostic differences between *Radula strangulata* and *Radula australiana* can again be derived by counting the number of rows of dorsal cortical cells that are not crossed by the leaf insertion lines. In *Radula strangulata* 2–5 rows are leaf-free, whereas no rows are leaf free in *Radula australiana*.

As far as is known, *Radula buccinifera* does not occur in New Zealand. However, it is always worth checking unusual plants against either of these species, particularly as the vagrant occurrence of *Radula buccinifera* in New Zealand cannot be ruled out. The reverse is also true, and some aquatic forms of *Radula buccinifera* are difficult to differentiate from *Radula strangulata*, and both species occur in Tasmania and south-east Australia. For guidance on separating *Radula strangulata* and *Radula buccinifera*, see the recognition section of *Radula buccinifera*.

**Remarks.** The variation in morphology exhibited by *Radula strangulata* seems to have encouraged the description of several species that we retain in synonymy. The type of *Radula strangulata* consists of small male plants on *Weymouthia cochlearifolia*, the lobules on this individualrepresent only a part of the range of variation expressed by the species. The other end of lobule shape variation, having heavily ampliate lobule base extending over the ventral stem surface, is found in the type specimen of *Radula levieri*. This specimen also has remote leaves and in these two characteristics corresponds to morphotypes associated with microsites that are permanently saturated. The type of *Radula silvosa* possesses lobules of the same shape range as exhibited by the type of *Radula strangulata*, however the plants are larger and female. Hodgson’s (1944) application of names is explained by her stated belief that the type of *Radula strangulata* was the male plant of *Radula levieri*. The type of *Radula parviretis* exhibits variable leaf size on single shoots, reddish pigments, variable leaf cell size, and grows on peat. Variation in cell size within leaves is not atypical of *Radula strangulata*. The collections made on peat may reflect occupancy of a rarely explored habitat. *Radula parviretis* is known from only two specimens, both from Port Pegasus on Rakiura (Stewart) Island. Reddish pigments are not known in other specimens of this species, and have not been observed in the field.

The plants illustrated for *Radula buccinifera* and *Radula silvosa* in Allison and Child (1975) are actually both *Radula strangulata*. The material illustrated for *Radula buccinifera* corresponds to a hygromorphic phenotype whereas the material illustrated for *Radula silvosa* corresponds with plants occupying drier microsites. Both drawings illustrate clearly how the dorsal stem surface is visible between the leaves, however as described above the perianth differences illustrated for these two species are misleading.

Hodgson (1944) identified a monoicous specimen of *Radula silvosa* collected by N. Kemble Walsch. This specimen (CHR587344) contains a packet labeled ‘perianth showing monoecious [sic]’ containing three shoots two of which are male and one of which is female. If the female shoot was ever attached to either of the male shoots, it was not so attached at the time of this investigation. Although the apex of one of the male shoots is missing, the sequence of leaves suggests that the female shoot does not comprise that apex, as the male shoot ends with an entire right leaf, and the female shoot begins with a fragment of the same. Furthermore, the remainder of the specimen consists of shoots that are either male, or female, or sterile. There is no definite evidence that this specimen is not a mixture of a male and a female individuals, a common occurrence for this, and other dioicous *Radula* species.

The specimen of *Radula strangulata* collected by J.D. Hooker in 1840 held in herb. Taylor (FH) can be regarded as the holotype, as it is cited in the protologue, is the only collection in herb. Taylor, and there are no additional collections in herb. Hooker derived from the Erebus & Terror voyage in either FH or BM. Duplicates of this specimen in S can be considered isotypes.

#### Specimens examined.

New Zealand: Kermadec Islands: Raoul Island, Ravine 8, Hebe Site, 29°14'0"S, 177°56'0"W, 147 m, 9 May 2009, *P.J. de Lange K373 & D.C. Havell*, AK313880; North Island: Te Paki Ecological Region and District, Te Paki, Tomokanga, Tomokanga Stream, 34°25'S, 172°57'E, 60 m, 22 Oct 2009, *P.J. de Lange 8503*, AK309079;Te Paki Ecological Region and District, Te Paki, Radar Bush, 34°28'03"S, 172°51'15"E, 160 m, 19 Sep 2011, *P.J. de Lange NC14 & M.A.M. Renner*, NSW970841; North Auckland, Waitakere Ranges, Cascade track, mid reaches of Cascade Stream, 36°53'37"S, 174°31'08"E, 124 m, 24 Feb 2012, *M.A.M. Renner 6265*, NSW896405; Hauraki Gulf, Rakitu (Arid) Island, Reserve Valley, 36°7'S, 175°30'E, 100 m, 4 Jan 1981, *E.K. Cameron 3*, AK312200; Coromandel Ecological Region, Colville Ecological District, Te Moehau, Moehau camp, track from Hope Stream, 36°33'S, 175°24'E, 580 m, 24 Nov 1971, *J.E. Braggins*, AK282576;Tainui Ecological Region, Raglan Ecological District, Te Akamu Waimai-Waikorea Road, 37°38'S, 174°49'E, 20 m, 17 Feb 2009, *P.J. de Lange 7813*, AK304757; *Colenso 2161*, ex herb. Hooker, NY01178967, as *Radula buccinata* Taylor; New Zealand, *Stephenson*, NY0118968 *p.p.*; New Zealand, *Hutton & Kirk*, NY01178969; New Zealand, *J.D. Hooker*, NY01178970; Auckland Ecological Region, Waitakere Ecological District, Waitakere Range, Spraggs Bush, 36°55'S, 174°32'E, 360 m, 4 Jan 2002, *M.A.M. Renner 02/11*, AK280392; Tongariro Ecological Region and District, Whakapapa River at Dropshaft, 39°8'S, 175°28'E, 700 m, 23 May 1989, *J.E. Braggins 89/008B*, AK255891;North Island, Hawkes Bay, Morere Hotel, Morere, on treefern trunk in dense bush, 21 Aug 1964, *R.E. Hatcher 3*, F;Otari Reserve, Wellington, April 1932, *Miss N. Kemble Welch No. 3*, CHR587344; Otari Reserve, Wadestown, Wellington, 13 Apr 1969, *B.G. Hamlin 1055*, WELT-H000470; South Island: Marlborough, Pelorus River catchment, head of Elvy Stream, 41°18'52"S, 173°34'24"E, 270 m, 12 Feb 2012, *M.A.M. Renner 6082*, NSW895357; Nelson, The Grove Scenic Reserve, Golden Bay, Pohara, 40°50'53"S, 172°52'13"E, 55 m, 19 Feb 2012, *M.A.M. Renner 6259*, NSW896393; Westland Land District, Croesus Track, Barrytown, 150 m, 9 Jun 1999, *D. Glenny 7841*, CHR525056; Whataroa Ecological Region, Hokitika Ecological District, south of Lake Kaniere on SE side of Mt Upright and north of Styx River, on alpine fault, 42°52'S, 171°9'E, 130 m, 26 Nov 1995, *J.E. Braggins 95654C*, AK286375; Westland, Paparoa National Park, Fox River., 42°02'26"S, 171°23'58"E, 20 m, 18 Feb 2012, *M.A.M. Renner 6222*, NSW895673; Westland, Arthurs Pass, McGrath Stream, above road bridge, 42°55'44"S, 171°33'22"E, 810 m, 14 Feb 2012, *M.A.M. Renner 6092*, NSW895367;

Australia: New South Wales: North Coast, Dorrigo National Park, west of Coffs Harbour, 30°22'S, 152°48'E, 550 m, 14 Apr 2011, *M.A.M. Renner 5265*, NSW875811; Tasmania: West Coast, Waratah-Savage River Road, Arthur River catchment, unnamed stream, 41°27'52"S, 145°25'26"E, 490 m, 28 Jan 2012, *M.A.M. Renner 6016 & E.A. Brown*, NSW909416.

### Excluded from the Australian Flora

#### Radula farmeri

Pearson. Journal of the Linnean Society, Botany 46: 29. 1922

##### Type.

Ignambi on rocks, 3000 ft, New Caledonia, 30 Jul 1914, *R.H. Compton*. 1530 N.C. (holotype: BM000825023!).

The type specimens of *Radula farmeri* and *Radula novae-hollandiae* (S-B43474!) share the same suite of characteristics;the leaf-lobe apex is obtuse to subacute, the lobules are longitudinally rectangular with a prominent notch in their apex within which sits the lobule papilla, the lobule marginal cells are irregularly crenulate, and the two specimens are also of equivalent size. The similarity between the types suggest the only substantive difference between *Radula farmeri* and *Radula novae-hollandiae* might be the perianth mouth, which is fimbriate in the former and entire in the latter. We have only Pearsons (1922) observations on this, as the type specimen of *Radula farmeri* in BM today bears no perianths.

##### Australian specimens of *Radula farmeri*.

Australia: Norfolk Island: Mt Pitt Road, Mount Pitt Reserve, 230 m, 29°1.5'S, 167°56.25'E, 2 Dec 1984, *H. Streimann 31867*, CANB650456, is *Radula* subg. *Odontoradula* sp. indet.; Mount Pitt Reserve, Filmy Fern Trail, off Selwyn Pine Road, 29°01'S, 167°58'E, 130 m, 3 Dec 1984, *H. Streimann 32084A*, CANB650459 is *Radula anisotoma* mixed with *Radula* subg. *Odontoradula* sp. indet.; Mount Pitt Reserve, Filmy Fern Trail, off Selwyn Pine Road, 29°1.3'S, 167°57.6'E, 130 m, 3 Dec 1984, *H. Streimann 32078* (CANB650457, NICH, NY, EGR, H) is *Radula anisotoma*.

##### Remarks.

*Radula farmeri* was recorded for Australia by So (2006). The specimens CANB650456 and CANB650457 are sterile, and while specimen CANB650459 is fertile the perianth mouth is entire. As identification of *Radula farmeri* is dependent on observation of perianths with fimbriate mouths; her determinations are not substantiated by the specimens, and this species should be excluded from the Australian flora.

## Supplementary Material

XML Treatment for Radula anisotoma

XML Treatment for Radula australiana

XML Treatment for Radula buccinifera

XML Treatment for Radula demissa

XML Treatment for Radula imposita

XML Treatment for Radula mittenii

XML Treatment for Radula notabilis

XML Treatment for Radula pugioniformis

XML Treatment for Radula strangulata

XML Treatment for Radula farmeri
